# Light-Induced Synthesis and Radiotheranostic Treatment of Gastric Cancer with ^161^Tb-Labeled Monoclonal Antibodies

**DOI:** 10.1021/jacsau.5c00219

**Published:** 2025-05-22

**Authors:** Patrick A. Cieslik, Dominik Roth, Eda Nisli, Jonas Genz, Cesare Berton, Pascal V. Grundler, Colin C. Hillhouse, Anzhelika N. Moiseeva, Mirja Nolff, Henrik Braband, Nicholas P. van der Meulen, Jason P. Holland

**Affiliations:** † Department of Chemistry, 27217 University of Zurich, Winterthurerstrasse 190, CH-8057 Zurich, Switzerland; ‡ 28498 Paul Scherrer Institute PSI, Forschungsstrasse 111, CH-5232 Villigen, Switzerland; § Klinik für Kleintierchirurgie, Vetsuisse-Fakultät, University of Zurich, Winterthurerstrasse 260, CH-8057 Zurich, Switzerland

**Keywords:** radiochemistry, photochemistry, radioimmunotherapy, terbium-161, gastric cancer, hepatocyte growth-factor receptor (c-MET), onartuzumab

## Abstract

Radiolabeled monoclonal antibodies (mAbs) form a major branch of nuclear medicine and are used in the development of tracers for both diagnostic imaging and molecularly targeted radio­(immuno)­therapy (RIT). Since treatment options for many types of late-stage cancers are limited and these diseases become refractory to classic chemotherapy, new tools are required to improve patient outcomes. The high tumor uptake and specificity of mAbs, coupled with increased therapeutic range of energetic β^–^-emitting radionuclides, offers a potential solution to overcome traditional problems associated with poor tissue penetration of antibody-drug conjugates, chemotherapeutic resistance, and off-target accumulation, which can lead to adverse responses. The challenge is to develop efficient and reliable chemical methods that provide simultaneous selectivity and high stabilization of the radiometal via complexation chemistry, with rapid access to new bioconjugate bonds on protein that avoid the loss of bioactivity. Here, we designed a new octadentate bispidine-chelating system, functionalized with a light-responsive tetrazole unit, and demonstrated the chemoselective derivatization of sulfhydryl groups introduced on the protein surface. High radiolabeling and bioconjugation yields of ^161^Tb-onartuzumab—an engineered mAb fragment targeting the human hepatocyte growth-factor receptor (c-MET; a characteristic biomarker found in clinical samples of several diseases, including gastric adenocarcinomas)—were obtained under ambient conditions after 5 min of light-induced coupling. Comprehensive biochemical and animal experiments including cellular binding assays, noninvasive γ-ray imaging, biodistribution studies, and pharmacokinetic measurements established the viability of using ^161^Tb-onartuzumab to target c-MET expression *in vivo*. Subsequent RIT studies in MKN-45 xenograft models demonstrated that the ^161^Tb-onartuzumab radiotracer formed by photoradiosynthesis permitted low-dose therapy studies that led to efficient targeting and treatment of tumor models. Collectively, the new complexation and chemoselective photoconjugation chemistries overcome some of the limitations in traditional labeling approaches. Photoradiosynthesis represents an excellent platform for building future antibody-based radiotracers for applications in diagnostic and therapeutic medicine.

## Graphical abstract

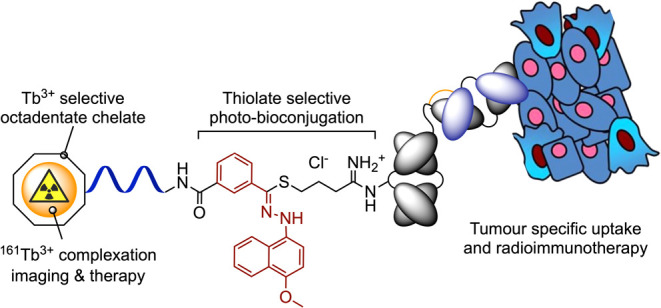

## Introduction

Since the advent of monoclonal antibodies (mAbs) by Milstein and Köhler in 1975,^1^ researchers and clinicians have sought to adapt their exquisite specificity and affinity for an increasingly diverse array of applications in biochemistry and medicine.^2^ In nuclear medicine, radiolabeled mAbs, and related antibody fragments, functionalized with different radionuclides, are a cornerstone of diagnostic imaging using positron emission tomography (PET)^3,4^ and of molecularly targeted radioimmunotherapy (RIT).^5–8^ RIT can target and destroy cancer cells via tumor-specific delivery of a cytotoxic therapeutic radionuclide payload.^9–11^ For applications in RIT, clinical-grade sources of radionuclides that emit high-energy beta-particles (β^–^, e.g., ^90^Y, ^131^I, ^161^Tb, ^177^Lu or ^186/188^Re, etc.) or alpha-particles (α, e.g., ^211^At, ^225^Ac, etc.) are chemically coupled to biologically active vectors, including tumor-specific peptides, mAbs, and related high-affinity engineered proteins, to create new classes of radiotherapeutic drugs that have the potential to overcome resistance mechanisms that often develop when using classic chemotherapeutic strategies. The peptide-based agent Pluvicto (^177^Lu-PSMA-617) is an archetype example of a recent clinical success story for molecularly targeted radiotherapy of advanced prostate cancer patients, for whom surgery was not an option, and where their disease was refractory to front-line chemotherapy options.^12^ Despite the proven success of using molecularly targeted radiation to treat patients with cancer, further work is required to develop new chelates, linkers, and bioconjugation methods to fine-tune the pharmacokinetics of the radiopharmaceuticals *in vivo*, to maximize target uptake in the tumor, while simultaneously reducing off-target accumulation in background organs, which can lead to dose-limiting toxicity and adverse responses.

^161^Tb is a promising radionuclide for the potential use in RIT.^13^ ^161^Tb shows similar decay properties to the well-established widely used ^177^Lu, where both radionuclides disintegrate via β-particle emission.^14–16^ ^161^Tb (*t*_1/2_ = 6.95 d, *E*_mean_(β^–^) = 154 keV, *E*_max_(β^–^) = 593.0 keV, total β-particle intensity *I*(β^–^) = 100%, and mean β-particle dose *D*_mean_(β^–^) = 0.156 MeV/Bq-s)^14,15^ has a slightly longer half-life than ^177^Lu (*t*_1/2_ = 6.647 d, *E*_mean_(β^–^) = 133.7 keV, *E*_max_(β^–^) = 496.8 keV, total β-particle intensity *I*(β^–^) = 100%, and mean β-particle dose *D*_mean_(β^–^) = 0.1336 MeV/Bq-s),^11,18^ but crucially, the decay of ^161^Tb releases more energy than ^177^Lu with *Q*-values of 593.0 keV *versus* 496.8 keV, respectively. Compared with ^177^Lu, the extra energy released during ^161^Tb decay increases the total dose by a factor of 1.36, with contributions from β-particles, Auger electrons, conversion electrons (CE) elevated by factors of 1.18, 9.63, and 2.95, respectively.^19–22^ Preclinical studies in tumor-bearing mice showed a more effective delay in tumor growth when using ^161^Tb- compared with ^177^Lu-based radioligands.^14,23,24^ However, experimental studies using ^161^Tb for RIT remain rare, and one limitation is the lack of suitable synthetic routes to access stable mAb-based radiotracers in high molar activity when using lanthanoids.

Our group recently established alternative methods for producing radiolabeled mAbs by using light-induced bioconjugation processes. These photoradiosynthesis methods enable rapid and straightforward access to radiolabeled mAbs using a variety of photoactivatable groups, such as aryl azides (ArN_3_) and tetrazoles.^25–32^ Notably, tetrazoles have been used in photolabeling reactions since 1967 ^33^ but were more recently explored as potential bioorthogonal reagents for “photoclick” chemistry.^34,35^ Detailed mechanistic studies^36,37^ and original literature^38^ disproved the biorthogonality of photoinduced reactions between tetrazoles and proteins, and nowadays, experimental studies have demonstrated reactivity with a wide range of reagents with reports of high selectivity toward amino,^39^ carboxyl,^40,41^ thiol,^42^ alkene, and alkyne^43^ groups, depending on the conditions employed. Our own photoradiosynthesis experiments with a novel [^89^Zr]­ZrDFO-tetrazole complex for light-initiated labeling of trastuzumab also confirmed that direct protein functionalization occurs but requires slightly basic conditions (pH around 7.5–9), pointing toward reactivity with amines.^26^ Further validation studies of photoradiolabeled tracers have been conducted with a variety of ligand structures, antibodies, and radionuclides, including ^64^Cu,^30^ ^68^Ga,^26,45,46^ ^89^Zr,^25,47^ and ^177^Lu.^48^ However, to advance this technology for applications in RIT, further improvements in isolated radiochemical yields and molar activities are essential.

In this study, we report the design, synthesis, and preclinical evaluation of a new photoactivatable complex to access ^161^Tb-labeled mAbs. Specifically, we used a bispidine-based^48–59^ scaffold to create an octadentate ligand system optimized for stable complexation of Tb^3+^ ions. The ligand was functionalized with a light-responsive tetrazole group to facilitate chemoselective labeling of free sulfhydryl residues introduced on to the proteins via the use of Traut’s reagent. After optimizing the radiochemistry for labeling the chelate with ^161^Tb, photoradiosynthesis was used to label onartuzumab (MetMAb; Genentech Inc. [Roche Group])—a humanized, one-armed monovalent antihuman c-MET antibody designed to bind to the extracellular domain of c-MET, blocking its activation by the hepatocyte growth factor (also known as scatter factor).^60^ Onartuzumab has a molecular weight of 99.16 kDa and a reported dissociation constant, *K*_d_, of 1.2 nM for binding of biotinylated-onartuzumab to wild-type human c-MET (*k*_on_ of 1.1 × 10^5^ M^–1^ s^–1^ and *k*_off_ of 1.9 × 10^–4^ s^–1^). The radiolabeled mAb, ^161^Tb-**12**-onartuzumab, was then studied through a comprehensive set of cellular binding experiments *in vitro*, animal imaging, biodistribution, and pharmacokinetic measurements, as well as radiotherapy studies in mouse models bearing the c-MET-positive and overexpressing MKN-45 gastric adenocarcinoma xenograft model. Experimental results demonstrated the potential of using bispidine chelate designs as well as light-induced bioconjugation methods to create viable ^161^Tb-based radiotracers for RIT.

## Methods

Full details on the methods, materials, synthesis, and characterization of all compounds are presented in the Supporting Information. Experimental NMR spectra and high-resolution electrospray ionization mass spectrometry (HR-ESI-MS) analyses are given in Figures S1–S21. Radiochemical and chromatographic data are also presented in Figures S22 and S23. Additional imaging data are presented in Figures S24 and S25, with further analysis of the biodistribution data shown in Figures S26 and S27, as well as in Table S1. Data on the measured tumor sizes are shown in Figure S28, and variations in animal weights are given in Figure S29. Additional data from ultrasound analysis of tumors are presented in Figures S30 and S31. Histological data are presented in Figures S32 and S33. Data from blood sample analysis are provided in Figure S34. Data associated with the dosimetric modeling are presented in Figure S35 and Table S2.

## Results and Discussion

### Synthesis of the Photoactivatable Chelate **12**

The synthesis of tetrazole-functionalized bispidine (**12**, Scheme 1) began with 1,5-dimethyl-3,7-diazabicyclo[3.3.1]­nonan-9-ol (compound **5**), which was prepared in four steps in accordance with a previously reported method.^54^ Selective *N*-alkylation of compound **5** with *N*-Boc-2-bromoethylamine afforded compound **6** in good yield, which then underwent *O*-alkylation with potassium *tert*-butoxide and methyl bromoacetic acid to give intermediate mixture **7**. Despite multiple purification attempts to isolate compound **7**, impurities persisted, and the sample was subjected to methyl ester saponification under aqueous basic conditions to yield the bifunctional bispidine scaffold (compound **8**). In parallel, a tetrazole-propylamine (Tz-propyl-NH_2_, compound **9**) was synthesized via a two-step procedure starting from the carboxylic acid derivative Tz-CO_2_H (Supporting Information, Figure S1), which was accessed via our reported methods.^26^ Amide coupling of Tz-CO_2_H with *mono*-*N*-Boc-propyl-1,3-diamine, followed by Boc-deprotection with trifluoroacetic acid (TFA) in dichloromethane, afforded compound **9** in a 55% yield over two steps. Next, the bispidine scaffold (compound **8**) was derivatized with the tetrazole reagent (compound **9**) via 1-ethyl-3-(3-dimethylaminopropyl)­carbodiimide (EDC)-mediated amide coupling in dimethylformamide (DMF), followed by Boc-deprotection *in situ* with TFA, to produce compound **10**. Subsequent alkylation of the two pendent primary amines with methyl bromoacetic acid gave compound **11**, from which the free carboxylic acid groups were revealed by saponification of the methyl esters under basic conditions to afford the photoactivatable octadentate chelate **12** (Bisp-Tz) with high purity (>95% measured by reverse-phase high-performance liquid chromatography [HPLC]). Full characterization data associated with the compounds presented in Scheme 1 are given in the Supporting Information, Figures S2–S17.

**Scheme 1:**
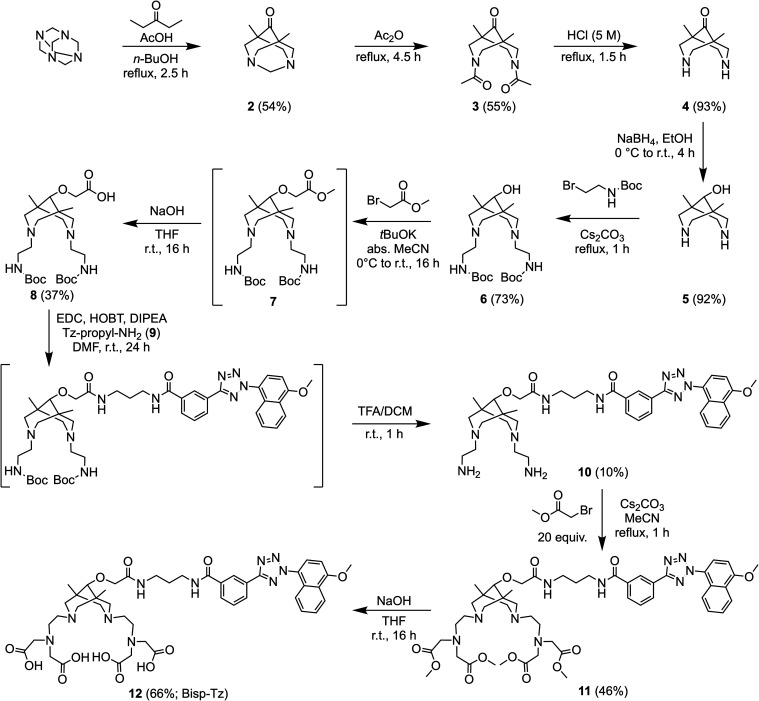
Synthetic Route to the Photoactivatable Octadentate Chelate **12** (Bisp-Tz); ^161^Tb-Radiochemistry

Before developing the photoradiosynthesis of ^161^Tb-antibodies, we optimized the complexation chemistry and radiolabeling conditions to form the monoanionic complex ^161^Tb-**12** ^–^. The nonradioactive complex ^nat^Tb-**12** ^–^ was prepared under conditions that are applicable to radiosynthesis with ^161^TbCl_3_, by incubating a solution of compound **12** in aqueous NaOAc (0.1 M, pH 5.5) with TbCl_3_ (1.2 equiv) at 40 °C for 5 min. The ^nat^Tb-**12** ^–^ formed quantitatively and was characterized by high-resolution electrospray ionization mass spectrometry (HR-ESI-MS; positive and negative ion modes) and analytical reverse-phase HPLC (see the Supporting Information, Figures S18–S22). The radiosynthesis of ^161^Tb-**12** ^–^ was accomplished under similar conditions by adding ^161^TbCl_3_ (499 MBq in 0.05 M HCl­(aq.)) to a solution containing compound **12** (5 nmol in 0.1 M NaOAc, pH 5.5), which was then stirred at 40 °C for 15 min. Quantitative radiolabeling, to give ^161^Tb-**12** ^–^ in a radiochemical purity of >99%, was observed as measured by radioactivity detection using instant thin-layer chromatography (radio-iTLC) and separately by reverse-phase high-performance liquid chromatography coupled with serial electronic absorption and radioactivity detection methods (radio-HPLC; Supporting Information, Figure S22). In both radio-iTLC and radio-HPLC, ^161^Tb-citrate was used as a control to distinguish the presence of unbound ‘free’ ^161^Tb^3+^ ions *versus* the desired complex ^161^Tb-**12** ^–^. Co-elution of the ^161^Tb-**12** ^–^ complex with a peak observed in the electronic absorption HPLC trace (254 nm) of the authenticated sample of ^nat^Tb-**12** ^–^ confirmed the identity of the radiolabeled complex. Rapid complexation to form the ^nat^Tb-**12** ^–^ or ^161^Tb-**12** ^–^ complexes quantitatively under mild conditions confirmed that the octadentate bispidine ligand is a rare example of a stable and highly effective chelate for complexation of Tb^3+^ ions, which holds potential in radiotracer design.

### Density Functional Theory Studies

The structure and bonding in a simplified model of ^nat^Tb-**12** ^–^ were investigated by using density functional theory (DFT) combined with Natural Bond Orbital (NBO) analysis implemented in Gaussian16 rev C.01. (Figure 1). Full computation details are provided in the methods section of the Supporting Information. Calculations employed the ωB97XD^61^ exchange-correlation functionals combined with the all-electron x2c-TZVPall-2c basis set^62^ (obtained from the Basis Set Exchange;^63^ https://www.basissetexchange.org) and a water continuum model. Geometry analysis showed minimal deviations in the bond lengths between the Tb^3+^ center and the nitrogen donors (mean bond length ± one standard deviation (SD), *r*_ave_(Tb–N) = 2.581 ± 0.038 Å) and the oxygen donor atoms (*r*_ave_(Tb–O) = 2.334 ± 0.040 Å). As anticipated, stronger electrostatic interactions between the Tb^3+^ cation and the carboxylate donors reduced the Tb–O bond lengths when compared with Tb–N distances. The optimized structure suggests that approximately equal contributions to the bonding and stabilizing interactions are formed between Tb^3+^ and the nitrogen and oxygen donor atom sets. This conclusion was supported by both the computed charges (based on natural population analysis, NPA) and from NBO analysis of the total (α + β) energies associated with ligand-to-metal stabilizing interactions (Figure 1). NBO analysis indicated that donation from nitrogen-based orbitals accounted for 43.2% while oxygen-based orbital interactions constituted 56.8% of the ligand-to-metal interactions. The donor atoms in ligand **12** create a well-balanced, almost spherically symmetric environment around the Tb^3+^ cation, which likely improves the thermodynamic stability of the complex. These calculations are in line with previous conclusions drawn from DFT studies on Lu-bispidine complexes, which indicated that adding four oxygen donor atoms to the octadentate coordination environment would likely increase the stability of lanthanoid ion complexes.^48^

**Figure 1:**
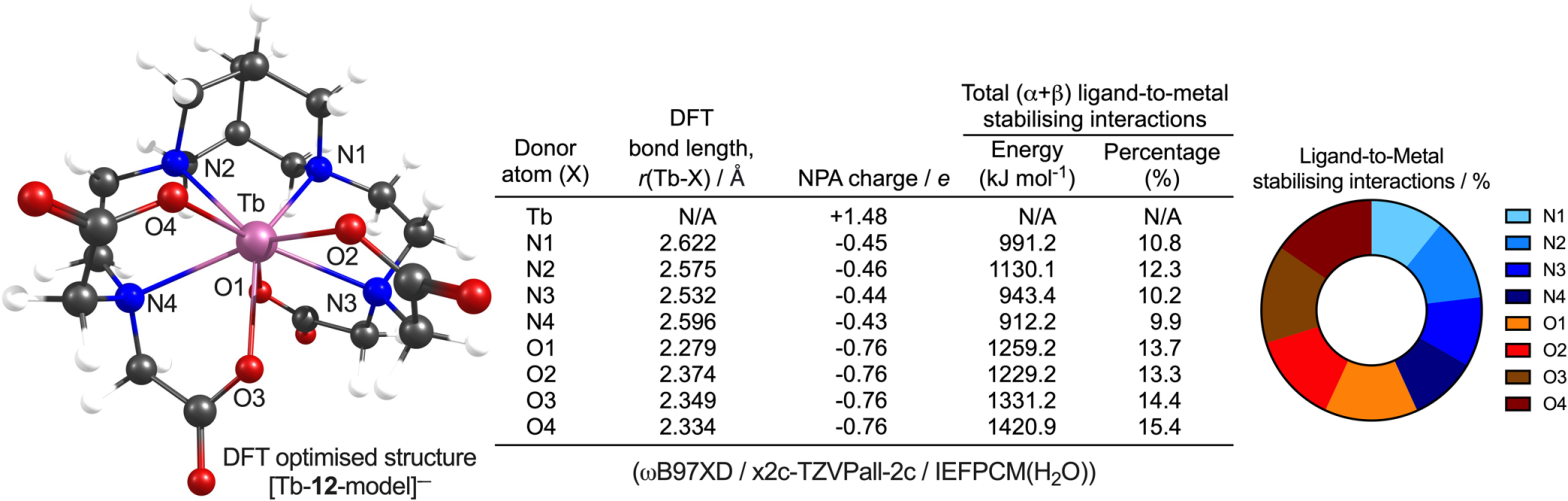
DFT optimized structure and natural bond orbital (NBO) analysis of simplified complex [Tb-**12**-model]^**–**^. Note, the calculated structure of [Tb-**12**-model]^−^ was simplified from that of the experimental structure by replacing the methyl and ether groups on the bispidine backbone with H atoms.

### Photoradiosynthesis of ^161^Tb-**12**-Onartuzumab

The photoradiosynthesis of ^161^Tb-**12**-onartuzumab was accomplished via the procedure outlined in Scheme 2. First, an aliquot of onartuzumab (formulated as the clinical-grade pharmaceutical MetMAb; Genentech/Roche)—an engineered monovalent monoclonal antibody^60^ that binds specifically to the human hepatocyte growth-factor receptor (c-MET)—was functionalized with free thiolate groups (Scheme 2A). The thiolated-onartuzumab (Thio-mAb) was prepared by the chemoselective reaction of primary ε-NH_2_ amine side-chain groups from surface-exposed lysine residues with Traut’s reagent (2-iminothiolane, 2 equiv, pH8, 20 min at room temperature).^64^ Successful derivatization of the protein with sulfhydryl groups was confirmed by using Ellman’s assay (see Supporting Information for details), which revealed an average ratio of 1.13 sulfhydryl groups per protein.^65^ It is important to prepare the Thio-mAb fresh and use it rapidly to reduce the potential formation of protein aggregates that can arise from disulfide bridge formation under oxidative conditions. In parallel, ^161^Tb-**12** ^–^ was prepared and characterized as described in the radiochemistry section above (Supporting Information Figures S20–S22). Then, ^161^Tb-**12**-onartuzumab was prepared by transferring an aliquot of ^161^Tb-**12** ^–^ to a clear, transparent glass vial containing the Thio-mAb solution. The initial stoichiometric ratio between compound **12** and mAb was set at 1:1 (Scheme 2B). After mixing the reagents and checking the pH, the solution was then irradiated with 365 nm UV-light for 5 min with gentle stirring (<100 rpm) at room temperature to give a crude sample of the desired compound ^161^Tb-**12**-onartuzumab (^161^Tb-**12**-mAb). The crude reaction mixture also contained ^161^Tb-radiolabeled small-molecule hydrolysis byproducts from photolysis of ^161^Tb-**12** ^–^. After the photoconjugation reaction, the crude mixture was quenched with iodoacetamide (8 equiv with respect to the mAb) for 30 min at room temperature and in the dark, to cap any free thiolate groups that remained on the protein and reduce protein aggregation. A small aliquot of the crude reaction mixture was retained for subsequent chromatographic characterization. The remainder of the crude mixture was purified by manual size-exclusion chromatography (SEC) using a PD-10 gel filtration column, followed by concentration of the high-molecular-weight protein fraction by using centrifugal spin filtration (30 kDa cutoff).

**Scheme 2:**
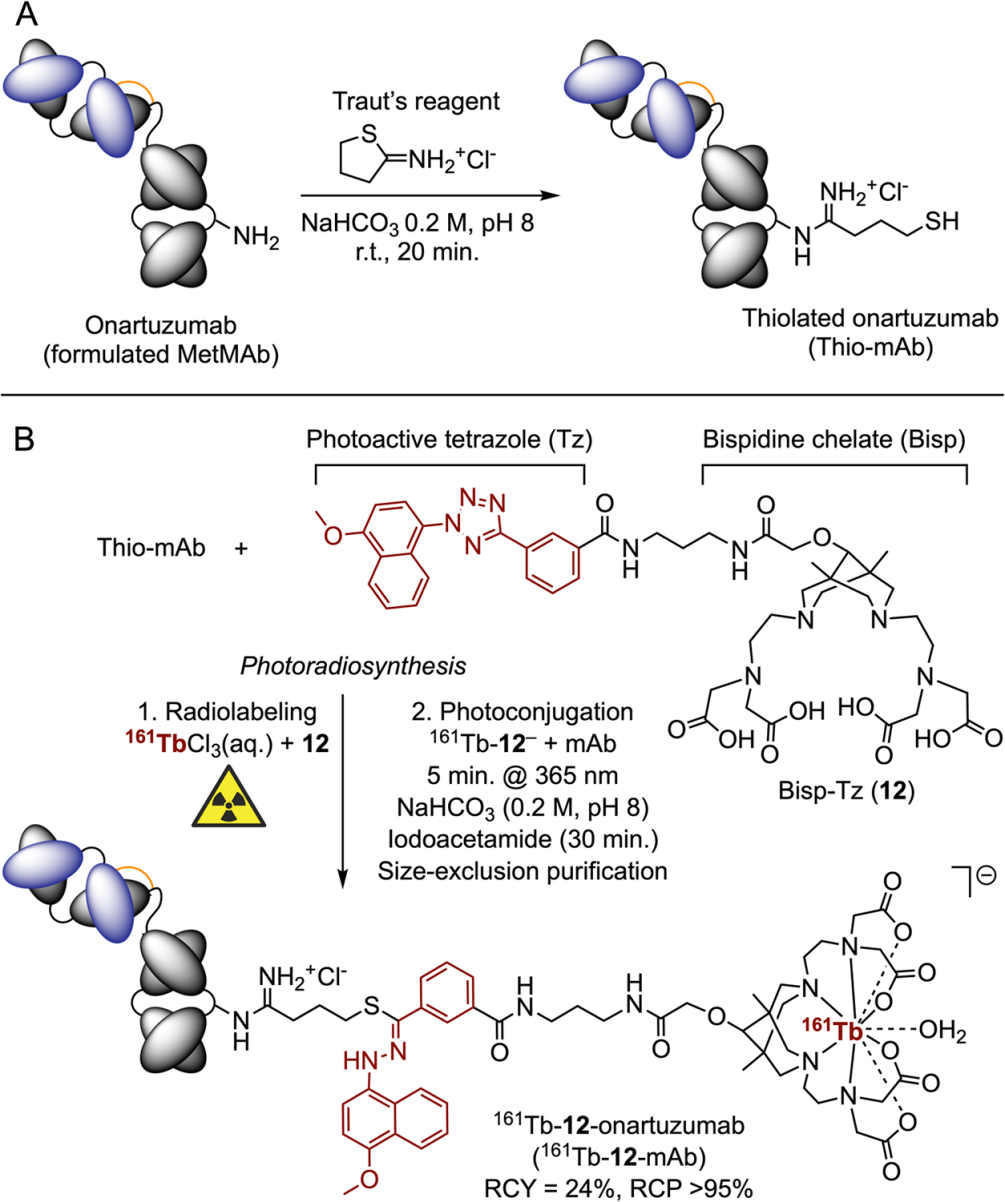
Photoradiosynthesis of ^161^Tb-**12**-Onartuzumab (^161^Tb-**12**-mAb)^a^

a (A) Introduction of thiol-groups to onartuzumab by using 2-iminothiolane to prepare thiolated-onartuzumab (Thio-mAb). (B) Multiple-component photoradiosynthesis of ^161^Tb-**12**-mAb starting from ^161^TbCl_3_, the photoactivatable chelate **12**, and a freshly prepared aliquot of the Thio-mAb in an initial chelate-to-mAb ratio of 1:1. After photolysis, the crude reaction was quenched with iodoacetamide, and the radiolabelled protein ^161^Tb-**12**-mAb was purified from radioactive small-molecule byproducts by a combination of size-exclusion methods, including PD-10 gel-column filtration and centrifugal spin filtration.

Aliquots of the crude and purified samples of ^161^Tb-**12**-mAb were analyzed by using three different chromatographic methods, including radio-iTLC (Figure 2A), manual size-exclusion chromatography (SEC) employing PD-10 gel filtration columns (Figure 2B), and HPLC coupled to a size-exclusion column (radio-SEC-HPLC; Figure 2C). Chromatographic data indicated that quantitative radiolabeling of **12** with ^161^Tb^3+^ ions occurred (Figure 2A, comparison of black and red traces), and that after the photoradiosynthesis step, the activity remained bound to the chelate with no free ^161^Tb^3+^ ions present in solution of the crude and purified samples of ^161^Tb-**12**-mAb (Figure 2A, green and orange traces, respectively). Chromatographic profiles obtained from manual analytical PD-10 analysis revealed that ^161^Tb-**12**-mAb was formed with a radiochemical conversion (RCC) of 27–30% in the crude reaction mixture (Figure 2B, green trace), which was separable from both unreacted ^161^Tb-**12** ^–^ and the majority (>95%) of the radioactive photolytic byproducts formed from control photolysis of ^161^Tb-**12** ^–^ in the absence of protein (Figure 2B, red and blue traces, respectively). After preparative PD-10 purification, the ^161^Tb-**12**-mAb had an RCP of >75% (Figure 2B, orange trace, prior to spin centrifugation). Analytical data obtained from the radio-SEC-HPLC measurements were consistent with the manual PD-10 data and revealed that ^161^Tb-**12**-mAb formed with a crude RCC of ∼27% (Figure 2C, green trace), and that the purified product was obtained with a final RCP > 95% after spin centrifugation. Overall, the purified ^161^Tb-**12**-mAb product was isolated with a decay-corrected radiochemical yield (RCY) of 23.5 ± 0.5% and a final molar activity of 11.2 MBq nmol^–1^ of protein. Note that the theoretical maximum molar activity for a ^161^Tb radiotracer with one Tb atom is 695.1 MBq nmol^–1^, and therefore, the obtained molar activity of 11.2 MBq nmol^–1^ gives an excellent isotopic dilution factor of only 62, confirming the high quality of the ^161^Tb-radiochemistry for mAb labeling.

**Figure 2:**
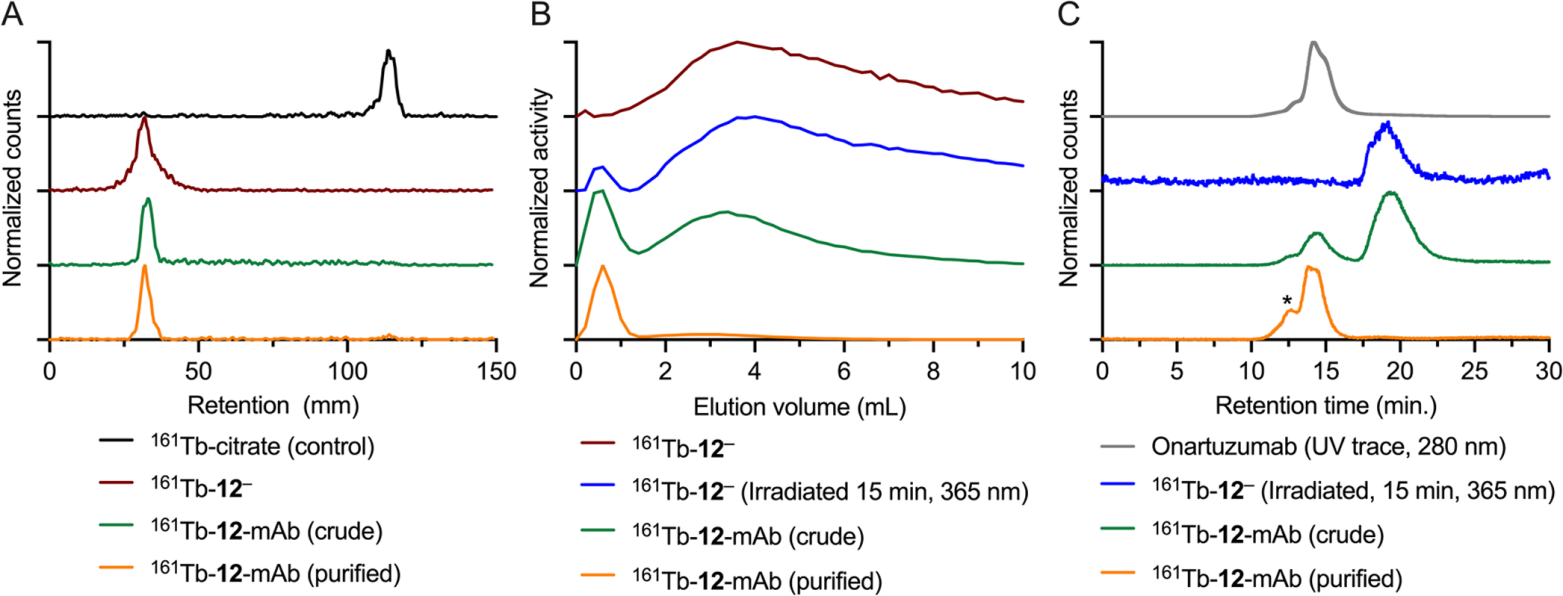
Chromatographic characterization of ^161^Tb-**12**-onartuzumab prepared by photoradiosynthesis. (A) Radio-iTLC chromatograms (eluted with aqueous disodium citrate, 0.1 M, pH 5.5) of ^161^Tb-**12** ^–^ (red trace), the crude mixture after irradiation for 15 min at 365 nm (green trace), the purified (orange trace) sample of ^161^Tb-**12**-mAb, and the control compound ^161^Tb­(citrate) (black trace). (B) Manual analytical PD-10 SEC elution profiles of ^161^Tb-**12**-mAb showing the crude (green trace) and purified samples (orange trace, radiochemical purity (RCP) > 95%), the nonirradiated dark control reaction (red trace) of ^161^Tb-**12** ^–^, and the irradiated radiolabeled complex in the absence of antibody (blue trace). (C) Radio-SEC-HPLC chromatograms of the crude (green trace) and purified (orange trace) samples of ^161^Tb-**12**-mAb, the small-molecule fraction (blue trace) collected by PD-10 SEC, showing the radioactivity channel and the electronic absorption trace at 280 nm of onartuzumab (gray trace). The asterisk (*) indicates the retention time of aggregated protein, which elutes before the main peak corresponding to monomeric ^161^Tb-**12**-mAb.

Tetrazole photochemistry is known to proceed via the formation of a reactive nitrile imine, which acts as either an electrophile for attack by biologically relevant nucleophiles or as a 1,3-dipolar reagent in [3 + 2]­cycloaddition photoclick reactions with unsaturated alkenes or alkynes.^26,31,35,66–69^ Separate control photoradiosynthesis experiments performed with ^161^Tb-**12** ^–^ reacting with either nonfunctionalized onartuzumab (fully formulated as the pharmaceutical MetMAb) or Thio-mAb revealed a 1.6–1.8-fold increase in the protein conjugation yield after introducing sulfhydryl groups (Supporting Information Figure S23). Notably, the nonfunctionalized onartuzumab does not present any free cysteine residues, and protein conjugation with the nitrile imine is expected to be mediated primarily via reaction with lysine residues (of which there are 66 lysines in the protein sequence, please see the Supporting Information for full protein sequence).^26,31^ Therefore, data from this control reaction point toward a chemoselective preference for the reactivity of the nitrile imine with sulfhydryl groups on protein.

### Stability Studies

Isolated samples of purified ^161^Tb-**12**-onartuzumab were incubated in human serum and in excess EDTA (pH7.4, 20 mM, 10^6^-fold excess) at 37 °C for up to 72 h (Figure 3A). The radiochemical stability, measured as the retention of ^161^Tb activity within the protein fraction versus small molecular components, was assessed by SEC-HPLC. After an initial decrease in RCP on protein by ∼20% at 48 h, ^161^Tb-**12**-mAb remained stable over 72 h in human serum. In contrast, competition with excess EDTA resulted in loss of the ^161^Tb activity from the protein fraction, with ∼70 and ∼80% of the radioactivity associated with the small-molecule fraction by 48 and 72 h, respectively. These EDTA ligand challenge experiments suggest that the bispidine chelate is kinetically labile in the presence of a powerful competitor, and some improvements in chelate design may be necessary, but we note that such high molar excesses of multidentate ligands are unlikely to occur *in vivo*. In addition, the high stability in the more biologically relevant human serum assay supports a further evaluation of ^161^Tb-**12**-mAb *in vivo*.

**Figure 3:**
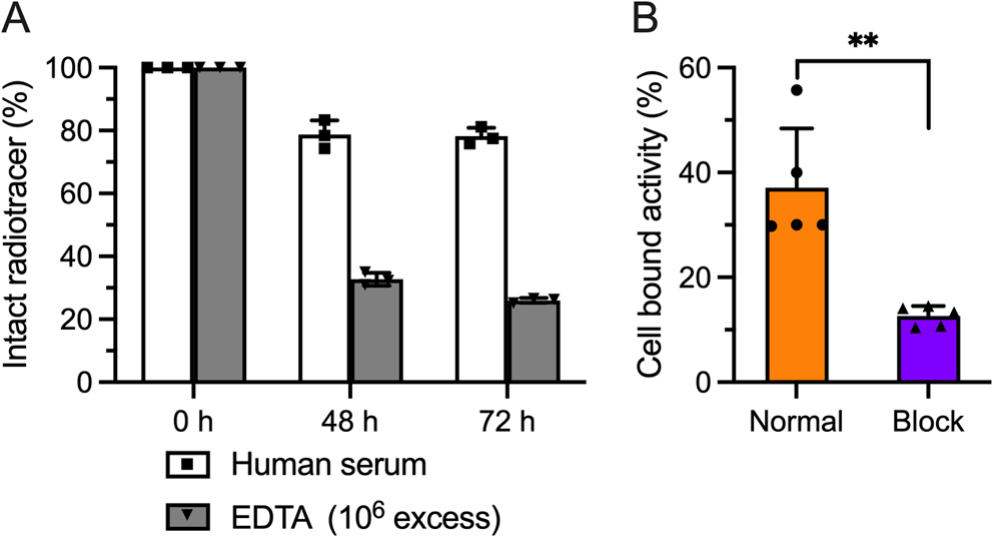
Stability assays and cellular binding of ^161^Tb-**12**-onartuzumab *in vitro*. (A) Radiotracer challenge experiments showing the stability of ^161^Tb-**12**-mAb in human serum (white bars, square data points) and a 10^6^ excess of EDTA (20 mM, pH7.4); (gray bars, inverted triangle data points) between 0 and 72 h (*n* = 3 independent replicates per group and per time point). (B) Cellular binding assays showing the percentage of bound activity (%) *versus* a standardized amount of activity drawn for the normal (orange bar, circle data points) and blocking (purple bar, triangle data points) doses of ^161^Tb-**12**-onartuzumab. Incubation of 1 × 10^6^ MKN-45 cells for 4 h at 37 °C (*n* = 5 independent replicates per group); purified radiotracer was added to each well to reach a final concentration of 5 ng mL^–1^ (note for blocking studies a total mass of 60 μg mAb per tube was used); error bars represent one standard deviation (SD) about the mean. Student’s *t*-test: (**) *P*-value < 0.01.

### Cellular Binding and Immunoreactivity

Binding and specificity of ^161^Tb-**12**-onartuzumab to the target protein were evaluated *in vitro* by using cellular association assays with c-MET-overexpressing MKN-45 gastric adenocarcinoma cells (Figure 3B). Competitive inhibition (blocking) studies were performed by using a reduced molar activity formulation of the radiotracer (prepared with an excess of nonradiolabeled antibody) to saturate the receptors. Experiments showed that ^161^Tb-**12**-mAb displayed specific binding to the c-MET receptor with an immunoreactive fraction of >37% (normal, *n* = 5), and >65% reduction in cellular uptake observed between the normal and blocking groups. These data confirmed that ^161^Tb-**12**-mAb remained biologically active and suitable for further use *in vivo*.

### Planar γ-Ray Scintigraphy Imaging

Next, we evaluated the tumor uptake and binding specificity in animal models of human gastric adenocarcinoma. Subcutaneous (s.c.) xenografts were developed on the right flank of female athymic nude mice by inoculation with 2 × 10^6^ MKN-45 cells in a 1:1 v/v mixture of growth media and Matrigel.^71^ After tumors established, animals were randomized into two groups (*n* = 5 mice/group), and individual animals were administered either a normal dose (*A*_m_ = ∼11.2 MBq nmol^–1^, 0.736–0.851 MBq) of activity (6.52–7.53 μg of protein, 0.066–0.076 nmol in 150 μL sterile PBS) or a blocking dose (*A*_m_(block) = ∼0.092 MBq nmol^–1^, 0.841–0.951 MBq (906.5–1025.0 μg of protein/mouse, 9.14–10.34 nmol in 150 μL sterile PBS) of ^161^Tb-**12**-onartuzumab via intravenous (i.v.) tail-vein injection (*t* = 0 h). Planar two-dimensional γ-ray scintigraphy imaging was performed over a period of 21 days at 0, 24, 72, 120, 168, 240, and 336 h postradiotracer administration (Supporting Information Figures S24 and S25). Data in Figure 4A show representative images of a mouse in the normal group recorded at 24, 72, and 168 h, alongside an image of a mouse in the blocking group at 168 h. The images were corrected for radionuclide decay and analyzed by using ImageJ to determine the accumulated activity fraction in the tumor, expressed as injected dose per cm^2^ (region-of-interest, [ROI] units in %ID cm^–2^; Figure 4B). ROI analysis of the tumor region indicated specific uptake at all time points, with decay-corrected peak accumulation of 5.17 ± 1.16%ID cm^–2^ for the normal group compared with 1.26 ± 0.38%ID cm^–2^ for the blocking group at 24 h (*P*-value < 0.001). These imaging data demonstrated the specificity of ^161^Tb-**12**-onartuzumab for the c-MET expression in the MKN-45 xenografts, which is consistent with biodistribution data recorded in this study (*vide infra*) and with previous reports using onartuzumab-based radiotracers in this model.^46,47,72–74^

**Figure 4:**
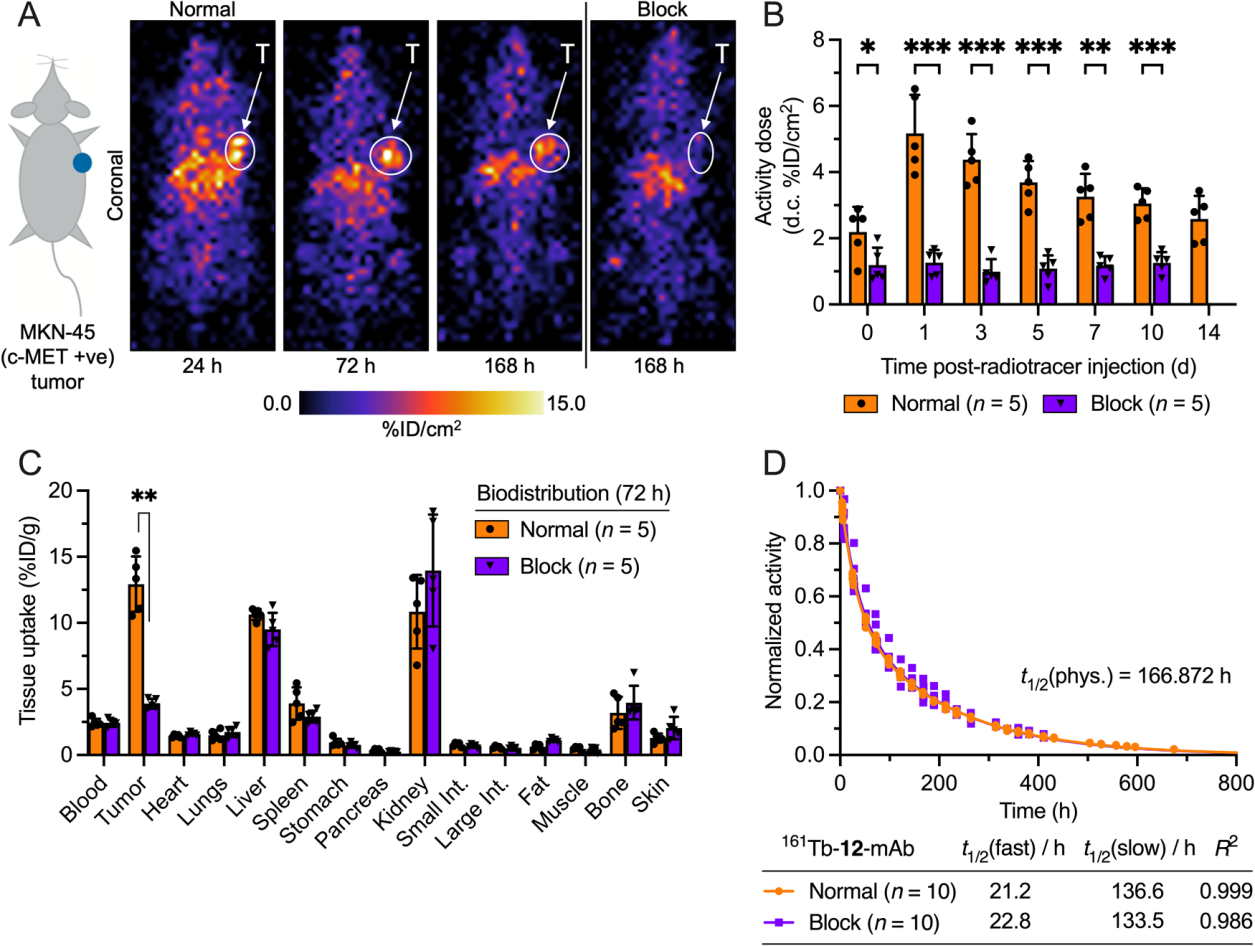
Molecular imaging, biodistribution, and pharmacokinetic data on ^161^Tb-**12**-onartuzumab in MKN-45 tumor-bearing mice. (A) Selected γ-ray scintigraphy images of a mouse from the normal group at 24 h, 72, and 168 h, and the block group at 168 h, after administration of ^161^Tb-**12**-onartuzumab. (B) Quantification of ROIs drawn around decay-corrected images showing tumor-associated activity in the normal (orange) and blocking (purple) groups (%ID cm^–2^). (C) Bar charts showing the full *ex vivo* biodistribution profile of ^161^Tb-**12**-onartuzumab (in units of %ID g^–1^) measured in MKN-45 tumor-bearing mice for the normal (orange) and blocking groups (purple) at 72 h postradiotracer administration. Bars show the arithmetic mean ± 1 standard deviation. (D) Plot of the normalized whole-body activity retained in the animals against time, after administration of ^161^Tb-**12**-onartuzumab in the normal group (orange) and the blocking group (purple). Curves show the two-phase exponential fit of the data with regression coefficients given in the table. Student’s *t*-test: (*) *P*-value < 0.05, (**) *P*-value < 0.01, and (***) *P*-value < 0.001.

### Biodistribution Studies

After the temporal imaging studies, two cohorts of animals (normal and blocking groups, *n* = 5 mice/group) were euthanised by terminal exsanguination under anesthesia at 72 h postadministration ^161^Tb-**12**-onartuzumab. Biodistribution studies were performed *ex vivo*, collecting 15 tissues, including tumors, to quantify accumulated ^161^Tb activity (Figure 4C; Supporting Information Table S1, and Figures S26 and S27). For animals in the normal group, accumulation of ^161^Tb-**12**-onartuzumab in the tumor reached 12.93 ± 2.09 %ID g^–1^. Comparison of tumor-associated activity between the normal group and the blocking group (3.89 ± 0.37 %ID g^–1^) confirmed the specific uptake and binding of ^161^Tb-**12**-onartuzumab (*P*-value < 0.01). Accumulation in the liver and spleen was also observed and is likely due to a small component of protein aggregation, resulting from the introduction of sulfhydryl groups during the preparation of the Thio-mAb and the photoradiosynthesis steps. Notably, kidney uptake was lower than previously observed in other radiolabeled onartuzumab conjugates, particularly in the blocking group, whereby a dose-dependent accumulation has been observed.^46,47,73,74^ This reduced kidney binding may be attributed to the nature of the bioconjugation bond formed during the light-induced activation of the tetrazole unit but could equally be the result of alteration of surface-bound lysine residues during the thiol-capping process using iodoacetamide. Further experiments are required to elucidate the effect of using iodoacetamide as a quenching reagent.

### Effective Half-Life Measurement

During the animal studies, whole-body activity was measured in each mouse by using a dose calibrator, and the data acquired were used to determine the effective (*t*_1/2_(eff)/h) half-life of the radiotracer *in vivo* (Figure 4D). Excretion profiles were obtained separately for the normal and blocking groups (*n* = 10 mice/group). In both cases, data from the normal and blocking groups were fitted with a two-phase decay, producing two elimination rate constants assigned to fast and slow processes. Data from the normal group of animals indicated that ^161^Tb-**12**-onartuzumab had a *t*_1/2_(fast) half-life of 21.2 h and a *t*_1/2_(slow) value of 136.6 h (regression coefficient, *R* ^2^ = 0.999). No difference was observed in the measured half-lives of the fast and slow excretion processes between the normal and blocking groups. These data were consistent with this observation confirmed the data observed in the biodistribution studies, supporting the conclusion that the pharmacokinetic profile of ^161^Tb-**12**-onartuzumab is the same for the normal and blocking groups in this animal model.

### Molecularly Targeted Radioimmunotherapy with ^161^Tb-**12**-Onartuzumab

Having established the pharmacokinetic profile and tumor-specificity of ^161^Tb-**12**-onartuzumab, we next evaluated the therapeutic potential of this radiotracer to inhibit tumor growth *in vivo*. Three groups of mice were monitored for up to 28 days postradiotracer administration (*n* = 10 mice/group, Figure 5). After randomizing the animals into groups, mice assigned to the normal treatment group received a single therapeutic dose of ^161^Tb-**12**-onartuzumab (*t* = 0 h) via intravenous tail-vein administration (*A*_m_(normal) = ∼11.2 MBq nmol^–1^, 3.17–3.68 MBq of activity) (mean activity = 3.50 ± 0.134 MBq, equivalent to 112.9 MBq mg^–1^, 28.1–32.6 μg of protein, and 0.283–0.328 nmol in 150 μL sterile PBS). Animals in the blocking treatment group received a low molar activity formulation (*A*_m_(block) = ∼0.355 ± 0.004 MBq nmol^–1^, mean activity = 3.69 ± 0.04 MBq, equivalent to 3.58 ± 0.04 MBq mg^–1^, 1032.3–1033.4 μg of protein, and 10.41–10.42 nmol in 150 μL sterile PBS). In addition, the third control group received no treatment. Animals were monitored daily throughout the experiment, and tumor size was measured with Vernier calipers (Supporting Information, Figure S28). No significant changes in animal behavior or weight were observed during the study (Supporting Information, Figure S29).

**Figure 5:**
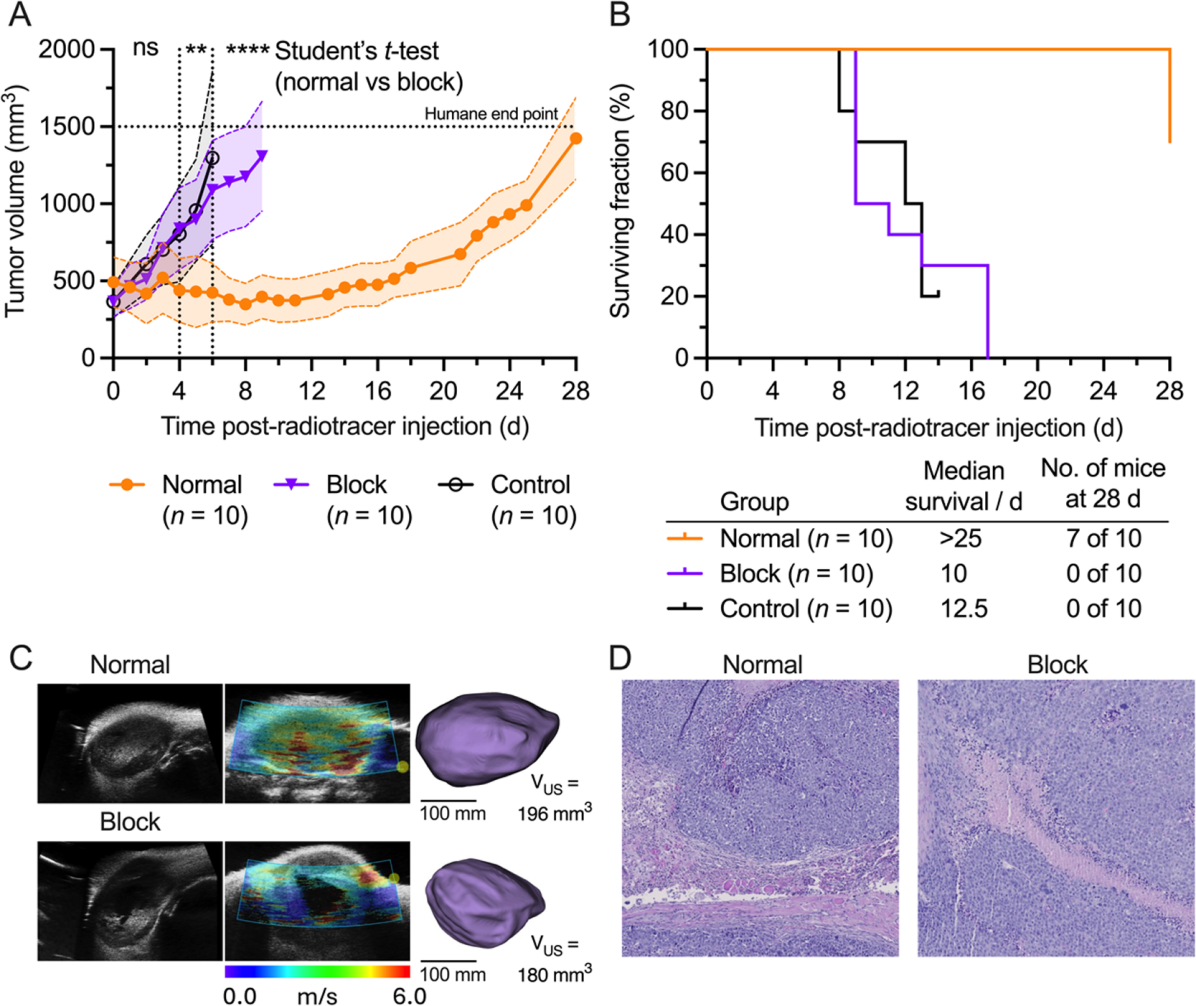
Assessment of the molecularly targeted radiotherapeutic efficacy of ^161^Tb-**12**-onartuzumab in mice-bearing MKN-45 gastric adenocarcinoma xenografts. (A) Tumor growth curves as measured by external Vernier callipers in the normal (orange circles, *n* = 10), block (purple squares, *n* = 10), and nontreated control (black circles, *n* = 10) cohorts. Tumor volume was calculated as ellipsoids from measurements of the length and orthogonal width of tumors. Student’s *t*-test: (**) *P*-value < 0.01; (****) *P*-value < 0.0001. (B) Kaplan–Meier plot showing the overall survival of animals in the normal (orange), blocking (purple), and nontreated control (black) groups. (C) Selected ultrasound images of normal (top) and blocking (bottom) mice showing the tumor at the 72 h time point in B-mode (left), ARF-mode (middle), and a three-dimensional (3D)-render of the tumor (right). (D) Representative microscopy images showing histological data from stained tumor slices (H&E stain) from the normal (left) and blocking (right) mice after reaching the humane end-point (set at a tumor volume >1500 mm^3^).

The therapy studies commenced with initial tumor volumes of 493 ± 162, 366 ± 96, and 184 ± 58 mm^3^ for the normal, blocking, and nontreated groups (*n* = 10 mice/group), respectively (Figure 5A). Measured tumor volumes showed a significant difference between the arithmetic mean for the normal group, with either the blocking or control groups at 4 days postradiotracer administration (*P*-value < 0.01). No significant difference was observed between the block and nontreated control groups. Tumors in the normal group regressed until 11 days postradiotracer injection, at which point tumor sizes stabilized at 374 ± 137 mm^3^. However, starting at 12 days postinjection, tumor regrowth occurred in all normal group mice. At this point, only ∼15% of the administered activity remained in the body, which appears to be insufficient to maintain arrest of tumor growth (for further discussion, see the section on dosimetry, *vide infra*). The blocking and nontreated control groups exhibited similar tumor growth patterns. Importantly, no differences were observed in mouse weight during the studies (Supporting Information Figure S29), indicating that the therapeutic doses (both activity dose and chemical dose) of ^161^Tb-**12**-onartuzumab were well-tolerated in animals of the normal and blocking groups.

A Kaplan–Meier survival plot is presented in Figure 5B. By day 8, two animals in the nontreated group had reached the humane end-point based on tumor volume, whereas for the blocking group, all animals remained in the study until day 9, when 50% of animals reached the end-point and were euthanised. In contrast, all animals in the normal treated group survived until the final day of the experiment (day 28) when three animals reached the end-point, giving an overall survival rate of 70%. In contrast, data from animals in the blocking and nontreated control groups gave median survival of 10 and 12.5 days, respectively (no significant difference). Statistical comparison of the normal group survival *versus* either the blocking group or nontreated control group showed a significant difference (*P*-value < 0.0001, in both comparison).

Tumor volume in the normal and blocking therapy groups was also assessed by using ultrasound (US) imaging (B-mode) at 0, 24, and 72 h time points, and at 336 h for the normal group (Figure 5C, Supporting Information, Figures S30 and S31). Although tumor volumes measured by ultrasound imaging displayed a markedly reduced size compared with the caliper measurements (a phenomenon associated with more accurate determination of the tumor margins by image analysis, which eliminated the contribution from skin and other experimental errors associated with user-dependent irreproducible errors), no significant differences in tumor volumes were observed between the normal and blocking groups during the first 72 h. These US imaging data are consistent with the observations made by using the external caliper.

Ultrasound, acoustic radiation force (ARF) elastography imaging was also performed at the 72 h time point to assess tissue stiffness (Figure 5C). Analysis of these elastography data indicated a tendency toward increased tissue stiffness in tumors of the normal group compared with the blocking group, as shown by regions of faster sound transmission. However, quantitative analysis of these data should be treated with caution since tumors from several animals in the blocking group displayed pronounced necrotic cores, which were not observed in tumors from the normal cohort. These morphological differences appear at an early time point (by day ∼3–4) when physical discrimination of the therapeutic response of tumors in the normal and blocking groups could not be established by tumor size measurements alone. Ultrasound elastography is potentially a valuable tool for monitoring the efficacy of chemotherapeutic or molecular targeted radionuclide therapies, but more studies are required to standardize the imaging protocols and determine the quantitative accuracy of the method.

Finally, after groups of animals reached the humane end-point, biodistribution studies were performed (Supporting Information Table S1) and tissue samples were removed for analysis by using both histological staining (Figure 5D, and Supporting Information Figures S32 and S33) and blood cytometry (Supporting Information Figure S34). Histological (hematoxylin and eosin; H&E) analysis revealed that the tumors of the normal group that rebounded after treatment (excised at the end of study at day 28) were morphologically similar to the tumors that developed at the end-point in the blocking group (excised from a subcohort of animals [*n* = 4] that were euthanised on day 17). Staining also revealed no significant differences in kidney tissues (the expected dose-limiting background organ for onartuzumab-based radioimmunotherapy) between the normal and blocking groups (Supporting Information Figure S33). Interestingly, hematology analysis measuring 23 different clinical (veterinary practice) parameters revealed no significant differences for any measurement taken between animals in the normal and blocking groups. These data are consistent with observations on mouse behavior and weight, providing additional convincing evidence that ^161^Tb-**12**-onartuzumab is well-tolerated and that the radiation exposure to background tissues in these experiments is below the threshold required to induce adverse physiological responses.

Collectively, the experimental data from these therapy studies support the conclusion that ^161^Tb-**12**-onartuzumab is a safe and effective treatment option for arresting gastric adenocarcinoma growth with low-dose administration.

### Radiotracer Dosimetry Analysis

From the imaging and biodistribution data obtained from the pilot and therapy studies (*vide supra*), combined with the known administered doses of ^161^Tb-**12**-onartuzumab in the normal and blocking groups, it is possible to estimate the radiation dose received by the tumor and critical background organs including the liver, kidneys, spleen, and bone (Figure 6, and Supporting Information Figure S35, and Table S2). Modeling of the radiotracer pharmacokinetics and dosimetry was performed by using our open-source online platform DoseItRight^©^ (https://doseitright.streamlit.app/).^22^

**Figure 6:**
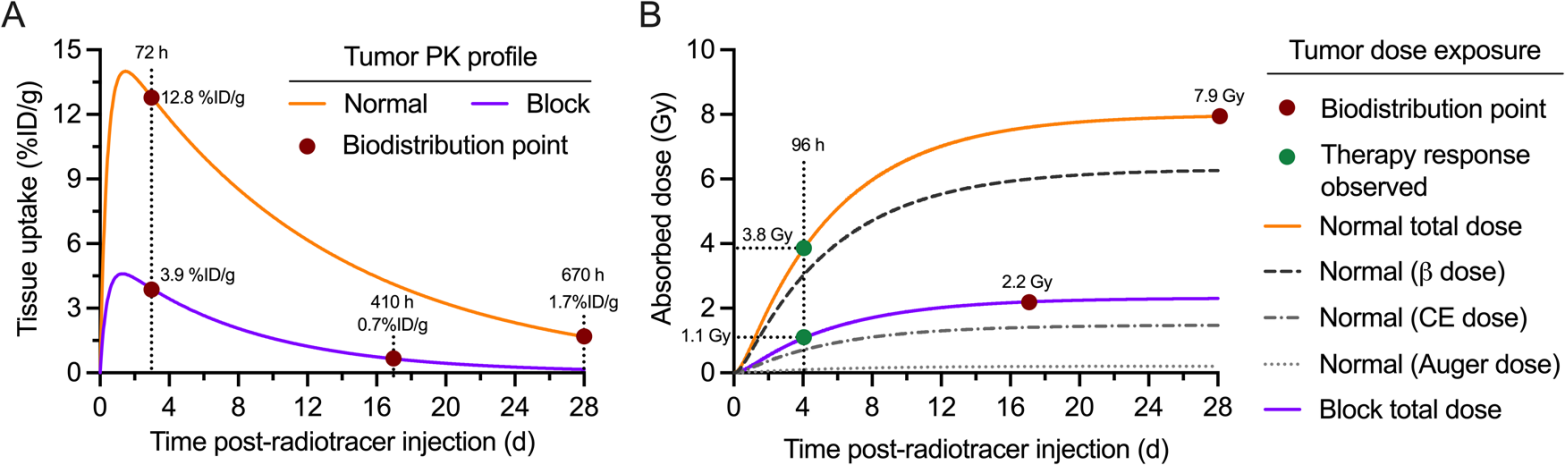
Pharmacokinetic profiles and modeled tumor absorbed dose for the normal and block group. (A) Calculated pharmacokinetic profile of ^161^Tb-**12**-onartuzumab uptake and washout in the MKN-45 xenograft (tumor) based on biodistribution and imaging data obtained from the normal (orange) and blocking (purple) groups. Red dots represent biodistribution time points (Supporting Information, Table S1). (B) Calculated absorbed dose for tumors in the normal (orange) and blocking (purple) groups. The first indications of therapy response, seen as statistically significant differences in tumor volume between the normal and blocking groups, were noted on day 4 (green points). The β-decay (black dashed line) of ^161^Tb decay is accompanied by emission of high-energy conversion electrons (CE; gray dotted and dashed line) and Auger electrons (gray dotted line), which contribute a total of 18.5% and 2.6% of the total radiation absorbed dose, respectively. Full parameters associated with the dosimetry modeling are given in the Supporting Information, Table S2.

First, the biphasic pharmacokinetic profile in tumor tissue was modeled by adjusting the values of the half-lives associated with tissue uptake (*t*_1/2_(in)/h) and elimination (*t*_1/2_(out)/h). Peak tumor uptake (in units of %ID g^–1^) was set based on a combination of data from the image analysis, which showed higher uptake at 24 h *versus* 72 h by a factor of 1.18, and from the biodistribution studies, which provided accurate quantification of the tissue uptake data at various time points for the two groups (indicated by the red dots in Figure 6A,B). The parameters were adjusted to ensure that the kinetic profile fit the observed experimental data. The experimental observations and modeled pharmacokinetic data are also consistent with our experience using onartuzumab in the development of many different radiotracers featuring alternative radiometal ions, chelates, linkers, and bioconjugate bonds.^46,47,73,74^ The half-life for tumor uptake of ^161^Tb-**12**-onartuzumab was approximately *t*_1/2_(in) ∼7 h, whereas the washout gave a value of *t*_1/2_(out) ∼205 h. Similar modeling data were observed for the blocking group with *t*_1/2_(in) ∼7 h, and *t*_1/2_(out) ∼230 h.

Based on this pharmacokinetic profile, the total absorbed dose *versus* time was calculated (Figure 6B). Notably, at 4 days postradiotracer administration, therapeutic effects were evident in the tumor volume analysis (Figure 5A). At this time point, the dosimetry modeling indicated that tumors in the normal group had already received a total of 3.8 Gy (Gray = J kg^–1^), whereas tumors from the blocking group, which failed to respond to treatment, only received 1.1 Gy. Of this total dose, 78.9% is associated with the emission of β-particles, 18.5% from high-energy conversion electrons (CE), and 2.6% from Auger electrons.^75^ By the humane end-point for the longest surviving animals in the blocking group, the tumor-associated adsorbed dose reached a maximum of 2.2 Gy (410 h; 17 days), whereas for animals in the normal group, the value was 7.9 Gy (670 h; 28 days). As expected, the primary dose to the normal tumors originates from β^–^-particles (*D*(β^–^) = 6.2 Gy, Supporting Information, Table S2), but CEs (*D*(CE) = 1.5 Gy) and Auger electrons (*D*(Auger) = 0.2 Gy) also contribute significantly.

Interestingly, at the end-point for animals in the normal group (670 h), background tissues also received high total doses (*D*(total)) that were comparable to the total dose received by the tumor (*D*(total) values for the liver, kidneys, and spleen were modeled at 7.7, 6.7, and 3.3 Gy, respectively; Supporting Information Table S2 and Figure S35). Since no adverse responses were observed in animal behavior, weight, or histological measures and blood analysis, it is believed that these doses to background organs do not negatively impact the well-being of the animals. Additionally, the total dose delivered to the tumor in the normal group at 410 h *versus* 670 h is 7.6 and 7.9 Gy, respectively, supporting the observation of tumor regrowth, as the delivered dose is insufficient to prevent recurrence.

From the empirical evidence of tumor growth and animal survival, it appears that the threshold radiation dose required for the onset of a physiological response of the tumor to the molecularly targeted radiation lies somewhere above 1.1 Gy but below 3.8 Gy. The MKN-45 gastric adenocarcinoma tumors appear to be highly sensitive to radiation exposure but a limitation of these experiments and modeling data is that it is not possible to discern the relative cytotoxic (or cytostatic) potential of the different contributors from ^161^Tb decay (β-particles, CE’s, and Auger electrons). Given that these particle emissions have very different ranges in tissue, their relative contribution to the observed cytotoxicity (or cytostasis) is likely to involve considerations of microdosimetry at the subcellular level. Optimizing the therapeutic response and elucidating the mechanisms of cell death and cell stasis associated with tumor exposure to ^161^Tb will require a more detailed analysis of radiotracer internalization and organelle sequestration of the radionuclide *versus* time.

## Conclusions

The experimental data from the chemical, radiochemical, and biological studies *in vivo* illustrate the potential of using bispidine-based chelates coupled to photoactivatable units to create viable radiolabeled antibodies for applications in diagnostic imaging and RIT. The light-induced conjugation process using tetrazoles was shown to be chemoselective for sulfhydryl groups, resulting in an efficient radiosynthesis of ^161^Tb-onartuzumab with excellent radiochemical yields and molar activities that facilitate therapeutic studies. Treatment of the MKN-45 gastric adenocarcinoma xenograft model with ^161^Tb-onartuzumab at low administered doses led to a pronounced therapy response that was detected within the first 4 days postradiotracer administration, as observed by external tumor volumetric measurements and supported by noninvasive ultrasound imaging, elastography, and tissue histology. Collectively, the experimental data suggest that with further development, the photoradiosynthesis approach used here can provide a new radiochemistry platform to improve the translation of ^161^Tb-radioimmunotherapeutics to the clinic. Specific advantages and limitations of using photochemical methods to radiolabel mAbs, and other biologically active proteins, have been discussed elsewhere,^28,45,77^ but one of the key advances is the ability to automate the chemistry for future clinical translation.^25^ We continue to work on the development of light-induced bioconjugation approaches to label mAbs, with high yields, shorter reaction times, and with improved radiotracer pharmacokinetics and reduced background tissue dosimetry.^22^

## Supplementary Material



## Notes

Full details of the experimental techniques employed, the synthetic procedures, and complete characterization data of all new compounds; additional details on the radiosynthesis, *in vitro* experiments, cellular binding studies, animal experimentation, imaging, and dosimetry calculations (PDF)
