# Supplementary material for: Light-Induced Synthesis and Radiotheranostic Treatment of Gastric Cancer with 161Tb-Labeled Monoclonal Antibodies
Source: JACS Au. 2025 May 22;5(6):2606–18. doi: 10.1021/jacsau.5c00219 (PMC12188480; doi:10.1021/jacsau.5c00219)
Supplement: Supplementary file 1 [file au5c00219_si_001.pdf]

Supplemental Information

Light-induced synthesis and radiotheranostic treatment of gastric cancer with ^{161}Tb-labelled monoclonal antibodies

Patrick A. Cieslik[1], Dominik Roth[1], Eda Nisli[1], Jonas Genz,[1] Cesare Berton,[1] Pascal V. Grundler[2], Colin C. Hillhouse[2], Anzhelika N. Moiseeva[2], Mirja Nolff,[3] Henrik Braband[1], Nicholas P. van der Meulen[2], and Jason P. Holland[1*]

[1] University of Zurich, Department of Chemistry, Winterthurerstrasse 190, CH-8057 Zurich, Switzerland.

[2] Paul Scherrer Institute PSI, Forschungsstrasse 111, CH-5232 Villigen, Switzerland.

[3] Klinik für Kleintierchirurgie, Vetsuisse-Fakultät, University of Zurich, Winterthurerstrasse 260, CH-8057 Zurich, Switzerland

*** Corresponding Author:**
Prof. Dr Jason P. Holland
ORCID: orcid.org/0000-0002-0066-219X
Tel: +41-44-63-53990
E-mail: jason.holland@chem.uzh.ch
Website: www.hollandlab.org
X (Twitter): @HollandLab_

First Author:
Dr Patrick Arthur Cieslik
ORCID: orcid.org/0000-0002-9709-3711
E-mail: patrick.cieslik@chem.uzh.ch

Table of Contents

Methods *5*

Chemicals and solvents 5

NMR spectroscopy 5

Mass spectrometry 5

High-performance liquid chromatography 5

Size-exclusion chromatography (SEC) 6

Density functional theory calculations 6

Radioactivity 7

Quantification of radioactivity 8

Ellman's assay 8

Photochemistry 9

Photograph S1. Image and schematic of the experimental photochemical set-up. 9

Cell culture 9

Cell binding studies 10

Animals and xenograft models 10

Planar γ-ray scintigraphy imaging 10

Ultrasound imaging 11

Biodistribution studies (*ex vivo*) 11

Therapeutic studies 11

Effective half-life measurements 12

Image analysis 12

Tissue dosimetry 12

Haematology analysis 13

Tissue processing & staining 13

Statistical analysis 14

Onartuzumab protein sequence 14

Synthesis and Characterisation *16*

Synthesis of compound 2 16

Synthesis of compound 3 16

Synthesis of compound 4 16

Synthesis of compound 5 17

Synthesis of compound 6 17

Synthesis of compound 8 17

Figure S1. Synthesis of Tetrazole-propyl-NH_2 (9) from Tz-CO_2H. 18

Synthesis of compound 9 18

Synthesis of compound 10 19

Synthesis of compound 11 19

Synthesis of compound 12 20

Figure S2. ^{1}H-NMR spectrum of compound 2 ($CDCl_3$, 400 MHz). 20

Figure S3. $^{13}C\{^1H\}$-NMR spectrum of compound 2 ($CDCl_3$, 101 MHz). 21
Figure S4. ^1H-NMR spectrum of compound 3 ($CDCl_3$, 400 MHz). 21
Figure S5. $^{13}C\{^1H\}$-NMR spectrum of compound 3 ($CDCl_3$, 101 MHz). 22
Figure S6. ^1H-NMR spectrum of compound 4 ($CDCl_3$, 400 MHz). 22
Figure S7. $^{13}C\{^1H\}$-NMR spectrum of compound 4 ($CDCl_3$, 101 MHz). 23
Figure S8. ^1H-NMR spectrum of compound 6 (CD_3CN, 400 MHz). 23
Figure S9. $^{13}C\{^1H\}$-NMR spectrum of compound 6 (CD_3CN, 101 MHz). 24
Figure S10. ^1H-NMR spectrum of compound 8 ($CDCl_3$, 400 MHz). 24
Figure S11. $^{13}C\{^1H\}$-NMR spectrum of compound 8 ($CDCl_3$, 101 MHz). 25
Figure S12. ^1H-NMR spectrum of compound 9 (CD_3CN, 400 MHz). 25
Figure S13. $^{13}C\{^1H\}$-NMR spectrum of compound 9 (CD_3CN, 101 MHz). 26
Figure S14. ^1H-NMR spectrum of compound 10 (D_2O, 400 MHz). 26
Figure S15. $^{13}C\{^1H\}$-NMR spectrum of compound 10 (D_2O, 101 MHz). 27
Figure S16. ^1H-NMR spectrum of compound 11 (DMSO-d6, 400 MHz). 27
Figure S17. $^{13}C\{^1H\}$-NMR spectrum of compound 11 (DMSO-d6, 101 MHz). 28
Figure S18. ^1H-NMR spectrum of compound 12 (D_2O, 400 MHz). 28
Figure S19. Reverse-phase analytical HPLC chromatogram of 12. 29
Synthesis and characterisation of ^{nat}Tb-12$^-$ 29
Figure S20. Reverse-phase analytical HPLC chromatogram of ^{nat}Tb-12$^-$. 29
Figure S21. HRMS (ESI-) spectrum of ^{nat}Tb-12$^-$. 29
Radiosynthesis of ^{161}Tb-12$^-$ 30
Figure S22. Chromatographic data on the characterisation of ^{161}Tb-12$^-$. 30
Photoradiosynthesis of ^{161}Tb-12-onartuzumab for biological experiments 30
Figure S23. SEC-HPLC chromatograms showing the radiotrace of crude samples of ^{161}Tb-12$^-$ prepared by reaction with either fully formulated MetMAb (purple) or MetMAb previously treated with 2-iminothiolane (orange). 31
Animal studies and preparation of ^{161}Tb-12-onartuzumab doses for injection in mice 31
Figure S24. Decay-corrected γ-scintigraphy images of the normal therapeutic group receiving ^{161}Tb-12-onartuzumab acquired at different time-points. 32
Figure S25. Decay corrected γ-scintigraphy images of the block therapeutic group receiving ^{161}Tb-12-onartuzumab acquired at different time-points. 33
Figure S26. Tumour-to-tissue contrast ratios measured at 72 h post-radiotracer administration in MKN-45 tumour bearing mice for ^{161}Tb-12-onartuzumab in the normal (orange) and blocking groups (purple). 33
Figure S27. Plot of the tissue uptake (%ID/g) *versus* time from biodistribution studies performed at various time points on the therapy groups. 34
Table S1. *Ex vivo* biodistribution data measured after i.v. administration of ^{161}Tb-12-onartuzumab in female athymic nude mice bearing subcutaneous MKN-45 tumours. 35
Figure S28. Individual tumour size as accessed per calliper measurement in (A) normal, (B) blocked, and (C) control cohort alongside the individual mouse weight in the (D) normal, (E) block, and (F) control group. 36
Figure S29. Mean mouse weight in the normal (orange), block (purple), and control (black) groups until end-point criteria were reached (n = 10). 36

Figure S30. Representative ultrasound images of normal mice showing the tumour morphology at 72 h (top) and 504 h (bottom) time-points in B-mode (left), acoustic radiation force, ARF-mode (middle), and a 3D-render of the tumour (right). ... 37

Figure S31. Ultrasound volumetric measurements on tumours up to 72 h post-radiotracer administration. ... 37

Figure S32. Confocal microscopy pictures of H&E stained tumours after reaching the end-point of the normal (top, 670 h) and block (bottom, 410 h) groups. ... 38

Figure S33. Confocal microscopy pictures of H&E stained kidneys after reaching the end-point of the normal (top, 670 h) and block (bottom, 410 h) groups. ... 38

Figure S34. Blood analysis of the samples taken from the normal (orange, 670 h) and blocking (purple, 410 h) groups at the end-point of the respective groups. ... 39

Figure S35. Calculated pharmacological kinetic profile and absorbed doses based on biodistribution and imaging data and for the normal (orange) and block (purple) group in different tissue. ... 41

Table S2. Fitting parameters for *DoseItRight*© and calculated absorbed doses in the normal and the blocking group in different tissue. ... 42

References ... 43

Methods

Chemicals and solvents

Unless otherwise stated, all other chemicals were of reagent grade and purchased from Sigma Aldrich (St. Louis, MO), Merck (Darmstadt, Germany), Tokyo Chemical Industry (Eschborn, Germany), abcr (Karlsruhe, Germany) or CheMatech (Dijon, France). Water (>18.2 MΩ·cm at 25 °C, Milli-Q® Direct 8/16 System, Milipore, Molsheim, France). Solvents for reactions were of reagent grade, and where necessary, were dried over molecular sieves. Solvent evaporation was performed under reduced pressure by using a rotary evaporator (Rotavapor R-300, Büchi Labortechnik AG, Flawil, Switzerland).

NMR spectroscopy

^{1}H- and $^{13}C\{^{1}H\}$-NMR spectra were measured in deuterated solvents on a Bruker AV-400 (^{1}H: 400 MHz, ^{13}C: 100.6 MHz) or a Bruker AV-500 (^{1}H: 500 MHz, ^{13}C: 125.8 MHz) spectrometer. Chemical shifts (δ) are expressed in parts per million (ppm) relative to the resonance of the residual solvent peaks, for example, with CD_3CN δ_H = 1.94 ppm and δ_C = 1.32 ppm with respect to tetramethylsilane (TMS, δ_H and δ_C = 0.00 ppm). Coupling constants (*J*) are reported in Hz. Peak multiplicities are abbreviated as follows: *s* (singlet), *d* (doublet), *dd* (doublet of doublets), *t* (triplet), *q* (quartet), *m* (multiplet), and *b* (broadened). Two-dimensional NMR experiments including ^{1}H-^{1}H correlation spectroscopy (COSY), ^{13}C-^{1}H heteronuclear single quantum coherence (HSQC) and ^{13}C-^{1}H heteronuclear multiple bond correlation (HMBC) were performed to aid the assignment of the ^{1}H and $^{13}C\{^{1}H\}$ spectra.

Mass spectrometry

High-resolution electrospray ionisation mass spectra, HRMS (ESI) were measured by the mass spectrometry service at the Department of Chemistry, University of Zurich.

High-performance liquid chromatography

Analytical high-performance liquid chromatography (HPLC) experiments were performed by using Hitachi Chromaster Ultra Rs systems fitted with a reverse phase VP 250/4 Nucleodur C18 HTec (4 mm ID × 150 mm, 5µm, Macherey-Nagel, Düren, Germany) column. This system was also fitted to a FlowStar^2 LB 514 radioactivity detector (Berthold Technologies, Zug, Switzerland) equipped with a 30 µL gamma cell (BGO-X, Berthold Technologies) for analysing radiochemical reactions. For HPLC chromatogram Figure S18, solvent A = 18.2 MΩ·cm H_2O+0.1% formic acid (FA) and solvent B = MeCN, and the method used a flow-rate of 0.5 mL min^{-1} with a linear gradient of A: *t* = 0 min 5% B; *t* = 15 min 100% B. For chromatogram Figure S19, solvent A = 18.2 MΩ·cm H_2O+0.1% formic acid (FA) and solvent B = MeOH+0.1% formic acid (FA), and the method used a flow-rate of 0.5 mL min^{-1} with a linear gradient of A: *t* = 0 min 5% B; *t* = 15 min 100% B; t = 18 min 100% B.

Size-exclusion chromatography (SEC)

Two SEC methods were used. The first method used a size-exclusion column (BioRad Laboratories, ENrich SEC 650, 10±2 μm, 10 mm ID × 300 mm) connected to an automated HPLC device (Rigol L-3000, Contrec AG, Switzerland) equipped with a UV-Vis (absorption measured at 280 nm) and an in-line radioactivity detector (FlowStar^2 LB 514, Berthold Technologies, Zug, Switzerland). Isocratic elution with phosphate buffered saline (PBS, pH7.4) was used for all SEC-HPLC analyses (flow-rate = 1 mL min^{-1}). The second method used manual SEC employing a PD-10 desalting column (Sephadex G-25 resin, 85–260 μm, 14.5 mm ID × 50 mm, >30 kDa, GE Healthcare). For analytical procedures, manual PD-10 SEC columns were eluted with sterile PBS. A total of 50×200 μL fractions were collected up to a final elution volume of 10 mL. Manual SEC columns were also used for preparative purification and reformulation of radiolabelled protein samples (sterile PBS, pH7.4).

Density functional theory calculations

Calculations were conducted by using density functional theory (DFT) as implemented in the Gaussian16 Revision C.01 suite of *ab initio* quantum chemistry programs.[1] Normal self-consistent field (SCF) and geometry convergence criteria were employed throughout. The structure was optimised in solution phase by using a polarisable continuum model (PCM) without symmetry constraints. Calculations were performed by using the ωB97XD[2] exchange-correlation functional and the x2c-TZVPall-2c basis set[3] from the Basis Set Exchange[4] website (https://www.basissetexchange.org). Solvated phase calculations were implemented by using the SCRF keyword with default parameters and selecting water as the solvent (dielectric constant, ε = 78.3553). The choice of solvation model reflects the aqueous phase conditions employed in the synthesis and applications of the bioconjugate. Harmonic frequency analysis based on analytical second derivatives was used to characterise the optimised structure as a local minimum on the potential energy surface. Optimised structures were analysed by using Chemcraft (version 1.8, build 536b). NBO analysis was performed using the default parameters in Gaussian16.

Cartesian coordinates of the optimized model [Tb-**12**-model]$^-$

```
65       0.234868000      0.000490000     -0.273676000
7       -2.051024000     -1.277939000     -0.156865000
7        0.668849000     -2.484230000     -0.053909000
7        1.186183000      2.077597000      0.959571000
7       -1.647124000      1.697204000      0.181802000
6       -3.640430000      0.330714000      0.925870000
6       -3.032953000     -1.049776000      0.920805000
1       -3.844320000     -1.783456000      0.818242000
6       -2.572165000     -0.850178000     -1.467537000
1       -3.357168000     -1.542655000     -1.801102000
6       -3.192667000      0.558103000     -1.445380000
6       -2.283794000      1.754729000     -1.148972000
1       -1.494935000      1.874567000     -1.891478000
1       -2.916049000      2.650579000     -1.214777000
6       -2.627150000      1.415018000      1.248149000
1       -3.184154000      2.334704000      1.468318000
1       -2.085744000      1.136466000      2.150945000
6       -4.311155000      0.580526000     -0.414311000
1       -5.051363000     -0.195530000     -0.619380000
6       -1.775061000     -2.721216000     -0.089755000
1       -1.776678000     -2.994386000      0.965067000
1       -2.592514000     -3.285561000     -0.553813000
```

6	-0.469622000	-3.153583000	-0.688242000
6	-1.024696000	2.995297000	0.494306000
1	-0.623310000	3.444034000	-0.411385000
6	0.061237000	2.817614000	1.529014000
1	-4.823558000	1.544440000	-0.425984000
1	-3.600621000	0.723872000	-2.443910000
1	-4.375959000	0.348813000	1.732285000
1	-0.446062000	-2.930711000	-1.754437000
1	-0.384874000	-4.242597000	-0.581296000
1	-1.781586000	3.696543000	0.860278000
1	-0.320618000	2.245091000	2.373731000
1	0.381017000	3.795757000	1.908764000
1	-2.528436000	-1.240478000	1.867086000
1	-1.775657000	-0.917357000	-2.216460000
6	0.815693000	-2.813154000	1.366488000
1	0.495150000	-3.835581000	1.585474000
1	1.875473000	-2.750877000	1.618942000
6	0.121028000	-1.836355000	2.330173000
6	1.865502000	-2.798913000	-0.831942000
1	2.741577000	-2.424765000	-0.303893000
1	1.983783000	-3.876373000	-0.986372000
6	1.825609000	-2.078861000	-2.182027000
6	2.087660000	1.578337000	1.995874000
1	2.714732000	2.367978000	2.421593000
1	1.497883000	1.144045000	2.804537000
6	2.964611000	0.455818000	1.435457000
6	1.933078000	2.907061000	0.008281000
1	1.952385000	3.953803000	0.327050000
1	2.968821000	2.564044000	-0.022247000
6	1.430781000	2.817976000	-1.429236000
8	4.042847000	0.211458000	1.974137000
8	2.460408000	-0.179414000	0.455266000
8	-0.151277000	-2.229614000	3.461585000
8	-0.074624000	-0.659374000	1.885719000
8	2.525598000	-2.503727000	-3.099552000
8	1.076853000	-1.048701000	-2.229920000
8	1.726507000	3.713119000	-2.218179000
8	0.760578000	1.773797000	-1.696949000

Radioactivity

Caution: Terbium-161 ($t_{1/2}$ = 6.95 d, $E_{mean}(\beta^-)$ = 154 keV, $E_{max.}(\beta^-)$ = 593.0 keV, total β-particle intensity $I(\beta^-)$ = 100%, mean β-particle dose $D_{mean}(\beta^-)$ = 0.156 MeV/Bq-s) emits β^- particles and high-energy gamma rays. All operations must be performed by qualified personnel in an approved facility and following safety guidelines set forth by the local authorities, and the Nuclear Regulatory Commission (or equivalent authority in your jurisdiction; for example, EU directive 2013/59/EURATOM of 5 December 2013 laying down basic safety standards for protection against the dangers arising from exposure to ionising radiation). Experimental manipulations should first be practised with non-radioactive samples and researchers should follow the ALARA (As Low As Reasonable Achievable) protocols to minimise exposure to ionising radiation.

Terbium-161

^{161}Tb was produced *via* neutron irradiation of enriched $^{160}Gd_2O_3$ (97.9% enrichment, Trace Sciences International, USA), by using the ^{160}Gd(n,γ)^{161}Gd→^{161}Tb nuclear reaction, as described by Gracheva *et al.*[5] The target material was dissolved, before being picked up in dilute ammonium nitrate and introduced to a macroporous cation exchange resin. The desired radionuclide was separated from the target material and impurities using α-HIBA, after which it was concentrated on a small column containing extraction resin. The $^{161}Tb^{3+}$ final product was eluted in a small volume of 0.05 M HCl.

Unless otherwise stated, ^{161}Tb-radiolabelling reactions were performed at 40 °C in NaOAc buffer (0.1 M, pH5.5). Radioactive reactions were monitored by using radio-TLC. Glass-fibre TLC plates were developed by using monosodium citrate (100 mM, pH5.5) as the eluent, and were analysed on a radio-TLC detector (Scan-RAM Radio-TLC Scanner & Analyzer, LabLogic, Sheffield, UK). RCC was determined by integrating the background-subtracted data obtained by the radio-TLC plate reader and determining both the percentage of radiolabelled non-photolysed radiocomplex (retention factor, R_f = 0.1–0.2) and 'free' ^{161}Tb (R_f = 0.9–1.0; present in the analyses as ^{161}Tb(citrate)). Small-molecule ^{161}Tb-radiolabelled products were characterised by analytical radio-HPLC. ^{161}Tb-labelled protein samples were characterised by manual SEC and automatic SEC-HPLC methods.

Quantification of radioactivity

Fractions obtained from manual SEC and tissues collected from the animal experiments were measured on a gamma counter (HIDEX Automatic Gamma Counter, Hidex AMG, Turku, Finland) by using a counting time of 30 s, and an energy window between 40–550 keV for ^{161}Tb (49 keV, 57 keV and 75 keV emission). Appropriate background and decay corrections were applied throughout.

Activity measurements were performed by using a dose calibrator (ISOMED 2010 Activimeter, Nuklear-Medizintechnik Dresden GmbH, Germany).

The radiochemical purity (RCP) of labelled protein samples was determined by automated SEC-HPLC (see above).

Ellman's assay

The content of free sulfhydryl groups in the antibody was assessed using Ellman's reagent following reaction with 2-Iminothiolane, as described in the literature.[6] Initially, a calibration curve was established by reacting increasing concentrations of L-cysteine (ranging from 0.05 mM to 0.5 mM) with an excess of 5,5′-dithio-*bis*(2-nitrobenzoic acid) (Ellman's reagent, 10.09 mM, 50 µL) in a phosphate buffer (0.1 M, pH 8), with a total reaction volume of 500 µL. The absorbance at 412 nm was recorded after 15 minutes using a NanoDrop™ spectrophotometer. To determine the sulfhydryl group content of the sample, a freshly prepared aliquot of Thio-mAb (17.1 mg mL^{-1}, 4.38 µL) was mixed with phosphate buffer (0.1 M, pH 8, 4.62 µL), followed by the addition of Ellman's reagent (0.991 µL), to reach a total reaction volume of 10 µL. After 15 minutes of incubation, the sulfhydryl content was quantified by measuring the absorbance at 412 nm (Abs = 0.34). From the previously recorded calibration curve measured by using L-cysteine, the average number of sulfhydryl groups per protein was determined to be 1.13-to-1.

The calibration curve is given below, and the dashed horizonal line shows the readout from the Ellman's assay performed on the functionalised protein prior to radiolabelling.

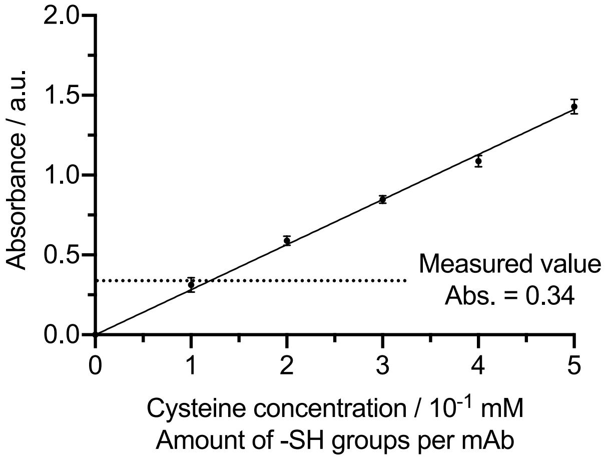

Photochemistry

Photochemical conjugation experiments were performed in transparent glass vials by using an ultra-violet light-emitting diode (LED; 365 nm). The LED intensity was adjusted by using a digital UV-LED controller (Opsytec Dr. Gröbel GmbH, Ettlingen, Germany), where 100% corresponded to a power of approximately 450 mW at 365 nm. LED intensity was measured by using a S470C Thermal Power Sensor Head Volume Absorber, 0.25–10.6 µm, 0.1 mW–5W, Ø15 mm. The LED had a maximum emission intensity at 364.5 nm. Photochemical reactions were stirred gently (stirring rate <100 rpm) to avoid potential mechanical damage to the protein. The temperature of all photochemical conjugation reactions was 23 ± 2 ºC. A photograph of the reaction set-up is shown below:

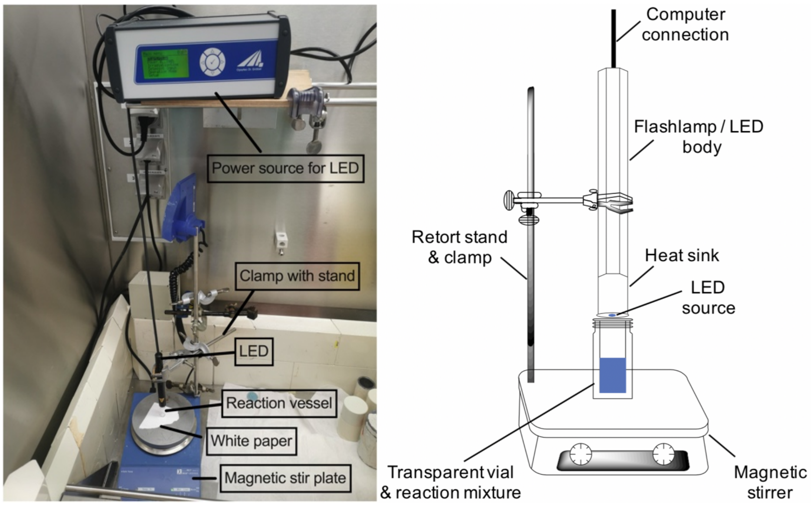

Photograph S1. Image and schematic of the experimental photochemical set-up.

Cell culture

The gastric cancerous cell line MKN-45 was obtained from the American Type Culture Collection [ATCC-HTB-77], Manassas, VA). Cells were cultured at 37 °C in a humidified 5% CO_2 atmosphere in RPMI media without phenol red supplemented with fetal bovine serum (FBS, 10% v/v, ThermoFisher

Scientific) and penicillin/streptomycin (P/S, 1% v/v of penicillin 10000 U mL^{-1} and streptomycin 10 mg mL^{-1}). Cells were grown by serial passage and were harvested using trypsin-EDTA solution (0.025%).

Cell binding studies

Cells were harvested and suspended in ice-cold sterile PBS (pH7.4). Then, approx. 1×10^6 MKN-45 cells were distributed in tubes and the purified radiotracer was added to each well to reach a final concentration of 5 ng mL^{-1} in quintuplicates and a total volume of 1 mL. To determine the extent of non-specific binding, a second series was prepared, and a specified quantity of non-radiolabelled sample was added to the cell suspension (60 μg per tube) in addition to the radiotracer. Cells were vigorously shaken at 25 °C for 4 h. The supernatant was collected separately for each tube and the cells washed three times with ice-cold PBS, which was also collected. Cell-associated radioactivity was measured with a gamma counter.

Animals and xenograft models

All experiments involving mice were conducted in accordance with an animal experimentation license approved by the Zurich Canton Veterinary Office, Switzerland (Jason P. Holland). Experimental procedures were planned and conducted in accordance with the ARRIVE 2.0 guidelines. Female athymic nude mice (Crl:NU(NCr)-*Foxn1^nu^*, 20–26 g, 4–6 weeks old) were obtained from Charles River Laboratories Inc. (Freiburg im Breisgau, Germany), and were allowed to acclimatise at the University of Zurich Laboratory Animal Services Centre vivarium for 1 week prior to experimentation. Mice were provided with food and water *ad libitum*. Tumours were induced on the right shoulder or flank by sub-cutaneous (s.c.) injection of 2×10^6 MKN-45 cells. The cells were injected in a 150 μL suspension of a 1:1 v/v mixture of growth medium and reconstituted basement membrane (Corning® Matrigel® Basement Membrane Matrix, obtained from VWR International). Tumours developed after a period of 6 days and the average volume of the MKN-45 tumours was 432 ± 136 mm^3 (n = 30 mice). Mice were randomised before the study.

Planar γ-ray scintigraphy imaging

Planar γ-ray scintigraphy imaging experiments were conducted on a γ-eye™ (BIOEMTECH, Athens, Greece). Approximately 3 minutes prior to recording each γ-ray image, mice were anesthetised by inhalation of between 2–4% isoflurane (Attane™, Piramal Critical Care, Inc, Bethlehem, PA, USA)/oxygen gas mixture and placed on the scanner bed in the prone position. γ-ray images were recorded at various time points between 1–336 h post-radiotracer administration. Anaesthesia was maintained by an experienced animal experimenter by controlling the isoflurane dose between 1.5–2.0% and monitoring respiration rate throughout the image acquisition. List-mode data were acquired for 10 min to 20 min for live-animal imaging. Detector signals were used to localise an event by employing the Anger logic.[7,8] System spatial resolution was measured with a ^{161}Tb capillary-source (40 mm long and a 1.1 mm inner diameter) placed at multiple distances (0 to 10 mm) from the detector

above the collimator. The Full Width at Half Maximum (FWHM) of the source profile was calculated by using a Gaussian fit. FWHM value (in mm) was calculated by multiplying the variance of the distribution σ with (2.35 + pixel size [1.45 mm × 1.45 mm]). Position mapping and uniformity correction are common practices for pixelated scintillator-based small field-of-view scintigraph systems. As a part of the γ-ray detector, position sensitive photomultiplier tubes (PSPMTs) often behave in a non-uniform manner. The correction methods involve position mapping, uniformity correction, and energy correction. By using the obtained raw flood image, a grid that maps each crystal pixel is determined. The counts in each crystal pixel are summed leading to the flood matrix, which corrects the intensity of the raw images. An energy window of ±20% is applied in each crystal pixel for energy correction. Attenuation correction is not applied. The γ-eye™ detector is linear for high count rates and activities up to 148 MBq, and therefore, at the activities applied in this study, dead-time count losses were not considered.

Ultrasound imaging

To assess tumour volumes and tissue stiffness, ultrasound imaging was conducted using a Prospect T1 system (Scintica Instrumentation Inc., London, Ontario, Canada) equipped with both a 40 MHz probe and a shear wave elastography probe. Mice were anaesthetised by inhaling a 2–4% isoflurane/oxygen gas mixture approximately 3 minutes before imaging and positioned prone on the scanning bed. A warm hydrogel (37 °C) was applied to the tumour, and an automated 3D mapping scan was performed with the electronic moving bed. For elastography, the probe was rotated 90° and the shear wave probe was positioned perpendicular to the scan area. Imaging analysis was conducted by using Prospect software, and 3D models were generated by using 3D Slicer (open source software: https://www.slicer.org).

Biodistribution studies (*ex vivo*)

Biodistribution studies were conducted at 72 h post-radiotracer administration to evaluate the radiotracer uptake and specificity in subcutaneous tumour-bearing mice. At the specified time point, animals (*n* = 5 mice / group) were anaesthetised individually by isoflurane and euthanised by isoflurane asphyxiation followed by terminal exsanguination. A total of 15 tissues (including the tumour) were removed, rinsed in water, dried in air for approx. 2 min., weighed and counted on a calibrated gamma counter for accumulation of activity. The mass of radiotracer formulation injected into each animal was measured and used to determine the total number of counts per minute (cpm) injected into each mouse by comparison to a standard syringe of known activity and mass. Count data were background- and decay-corrected, and the tissue uptake for each sample (determined in units of percentage injected dose per gram [%ID g^{-1}]) was calculated by normalisation to the total amount of activity injected for each individual animal.

Therapeutic studies

Animals were randomised and divided in two groups (*n* = 10 per group), of which the normal group was administered a high molar activity dose of the radioactive antibody (3.17 – 3.68 MBq, mean activity =

3.50±0.134 MBq, equivalent to 112.9 MBq mg^{-1}, 28.1 – 32.6 µg of protein, 0.283 – 0.328 nmol in 150 µL sterile PBS; A_m(normal) = ~11.2 MBq nmol^{-1}, onartuzumab MW = 99.16 kDa). The blocking group received a 2-fold lower molar activity dose (3.65 – 3.78 MBq, 3.69±0.04 MBq, equivalent to 3.58±0.04 MBq mg^{-1}, 1032.3 – 1033.4 µg of protein, 10.41 – 10.42 nmol in 150 µL sterile PBS). Note that samples of the radiotracer were drawn from the same production run (details on the photoradiosynthesis are given below).

The tumour volume (V / mm^3) was estimated by external vernier calliper measurements of the longest axis, a / mm, and an axis perpendicular to the longest axis, b / mm. The tumours were assumed to be ellipsoidal, and the volume was calculated in accordance with Equation 1. Tumour volume was monitored over the course of the experiment for up to 28 days post-radiotracer administration. As a humane end point, animals with tumour volume >1500 mm^3 were euthanised by exsanguination under anaesthesia and tissue samples removed for biodistribution studies and tissue staining.

$$V = \frac{4\pi}{3} \cdot \left(\frac{a}{2}\right)^2 \cdot \frac{b}{2} \quad \text{(Equation 1)}$$

Effective half-life measurements

The effective half-life $t_{1/2}$(eff) / h in the animal model was calculated from the measurement of total internal radioactivity *versus* over time by using a dose calibrator.

Image analysis

Images were processed by using the program package ImageJ (Version 1.51) and images were generated by using Python implemented in Jupyter Notebook (https://jupyter.org). All images were corrected for the physical decay and the activity per pixel to obtain activities as injected dose per cm^2 [ID cm^{-2}]. For image quantification, region-of-interest (ROI) analysis was performed by drawing ROIs around the tumour, and background subtraction was performed by using ROIs drawn in blank regions in each image.

Tissue dosimetry

Tissue dosimetry was estimated by using the open-source online tool *DoseItRight*© (https://doseitright.streamlit.app).[9] The two half-lives associated with tissue uptake and washout ($t_{1/2}$(in) and $t_{1/2}$(out), respectively), and the peak tissue uptake (ϕ_{max}, %ID g^{-1}) for the pharmacokinetic profile were estimated based on the experimental biodistribution and imaging analysis data available from this study. Dose delivery profiles were calculated for ^{161}Tb as nuclide and initial activities of 3.50 MBq for the normal and 3.70 MBq for the block group, representing the mean administered doses during the therapeutic study.

Haematology analysis

Blood analysis was performed directly after euthanasia on blood drawn from the heart on an InSight V5 Haematology Analyzer (Woodley Veterinary Diagnostics, Bolton, Lancashire, United Kingdom).

Tissue processing & staining

Tissues for staining were removed and directly submerged in neutral buffered formalin (phosphate buffer) for fixation. After 24 h, tissues were dehydrated, cleared and infiltrated with histological wax by using an automatic enclosed tissue processor (Donatello Series 3, Diapath S.p.A., Martinengo, Italy). Tissues were subsequently put into wax blocks and sliced on a microtome. Tissue slices (5 μm) were subsequently put on microscopic slides, dewaxed, stained with Haematoxylin and Eosin and cover slipped with Organo/Limonene by using the procedure stated in the table below. Pictures were acquired at the Center for Microscopy and Image Analysis (University of Zurich, Switzerland), and images were analysed by using the Zeiss ZEN lite software package.

Solution	Time (min)	Volume	Step
Xylene	2	100 mL	Dewax
Xylene	2	100 mL	Dewax
100% ethanol	2	100 mL	Dewax
100% ethanol	2	100 mL	Dewax
95% ethanol	2	100 mL	Dewax
Water	2	100 mL	Hydration
Haematoxylin	3	100 mL	Stain
Water*	1	100 mL	Remove excess stain
Differentiator**	1	100 mL	Remove excess stain
Water	1	100 mL	Remove excess stain
Bluing	1	100 mL	Complete stain
Water	1	100 mL	Remove excess stain
95% ethanol	1	100 mL	Remove excess stain
Eosin	0.75	100 mL	Counter stain
95% ethanol	1	100 mL	Remove excess stain
100% ethanol	1	100 mL	Remove excess stain
100% ethanol		100 mL	Remove excess stain
Xylene	2	100 mL	Dehydrate
Xylene	2	100 mL	Dehydrate
Organo/Limonene	-	-	Coverslip

* Water wash was exchanged after every Haematoxylin staining

** Differentiator solution: 1 mL HCl (32%) in 400 mL 70% ethanol

Statistical analysis

Where appropriate, data were analysed by the unpaired, two-tailed Student's *t*-test. Differences at the 95% confidence level, (*) *P*-value <0.05, were considered statistically significant. Note: (**) *P*-value <0.01; (***) *P*-value <0.001. Data from haematology studies on blood samples were analysed by using Welch's *t*-test implemented in GraphPad Prism (version 10.0.0 for Mac, Boston, Massachusetts USA).

Onartuzumab protein sequence

Data retrieved from https://gsrs.ncats.nih.gov/ginas/app/ui/substances/MS1J9720WC (accessed 31/05/2025). For onartuzumab, there are 66 lysine residues in total distributed over three protein subunits (highlighted in yellow).

**Subunit 1(31 lysine residues)**	**Amino acid number**
E V Q L V E S G G G	10
L V Q P G G S L R L	20
S C A A S G Y T F T	30
S Y W L H W V R Q A	40
P G K G L E W V G M	50
I D P S N S D T R F	60
N P N F K D R F T I	70
S A D T S K N T A Y	80
L Q M N S L R A E D	90
T A V Y Y C A T Y R	100
S Y V T P L D Y W G	110
Q G T L V T V S S A	120
S T K G P S V F P L	130
A P S S K S T S G G	140
T A A L G C L V K D	150
Y F P E P V T V S W	160
N S G A L T S G V H	170
T F P A V L Q S S G	180
L Y S L S S V V T V	190
P S S S L G T Q T Y	200
I C N V N H K P S N	210
T K V D K K V E P K	220
S C D K T H T C P P	230
C P A P E L L G G P	240
S V F L F P P K P K	250
D T L M I S R T P E	260
V T C V V V D V S H	270
E D P E V K F N W Y	280
V D G V E V H N A K	290
T K P R E E Q Y N S	300
T Y R V V S V L T V	310
L H Q D W L N G K E	320
Y K C K V S N K A L	330
P A P I E K T I S K	340
A K G Q P R E P Q V	350
Y T L P P S R E E M	360
T K N Q V S L S C A	370
V K G F Y P S D I A	380
V E W E S N G Q P E	390
N N Y K T T P P V L	400
D S D G S F F L V S	410
K L T V D K S R W Q	420
Q G N V F S C S V M	430
H E A L H N H Y T Q	440
K S L S L S P G K	449
**Subunit 2 (20 lysine residues)**	**Amino acid number**
D K T H T C P P C P	10
A P E L L G G P S V	20
F L F P P K P K D T	30
L M I S R T P E V T	40
C V V V D V S H E D	50
P E V K F N W Y V D	60

G V E V H N A K T K	70
P R E E Q Y N S T Y	80
R V V S V L T V L H	90
Q D W L N G K E Y K	100
C K V S N K A L P A	110
P I E K T I S K A K	120
G Q P R E P Q V Y T	130
L P P S R E E M T K	140
N Q V S L W C L V K	150
G F Y P S D I A V E	160
W E S N G Q P E N N	170
Y K T T P P V L D S	180
D G S F F L Y S K L	190
T V D K S R W Q Q G	200
N V F S C S V M H E	210
A L H N H Y T Q K S	220
L S L S P G K	227
**Subunit 3 (15 lysine residues)**	**Amino acid number**
D I Q M T Q S P S S	10
L S A S V G D R V T	20
I T C K S S Q S L L	30
Y T S S Q K N Y L A	40
W Y Q Q K P G K A P	50
K L L I Y W A S T R	60
E S G V P S R F S G	70
S G S G T D F T L T	80
I S S L Q P E D F A	90
T Y Y C Q Q Y Y A Y	100
P W T F G Q G T K V	110
E I K R T V A A P S	120
V F I F P P S D E Q	130
L K S G T A S V V C	140
L L N N F Y P R E A	150
K V Q W K V D N A L	160
Q S G N S Q E S V T	170
E Q D S K D S T Y S	180
L S S T L T L S K A	190
D Y E K H K V Y A C	200
E V T H Q G L S S P	210
V T K S F N R G E C	220

Synthesis and Characterisation

Experimental details on the synthesis and characterisation data related to the structures shown in Scheme 1 in the main text.

Synthesis of compound 2

In a round-bottom flask (250 mL) attached to a reflux condenser, hexamethylene tetramine (27.1 g, 193 mmol, 1.0 eq.), 3-pentanone (24.6 mL, 233 mmol, 1.2 eq.) and glacial acetic acid (24.5 mL, 424 mmol, 2.2 eq.) were dissolved in *n*-BuOH (80 mL). The mixture was stirred under reflux for 2.25 h, after which the solvent was removed under reduced pressure. Solid-phase extraction was performed on the remaining solid by sonicating in cyclohexane (200 mL) for 10 minutes and decanting the liquid. This process was repeated 2 more times. To the combined organic phase, aluminium oxide (20 g) was added, which was stirred at 80 °C for 30 minutes. After removing the aluminium oxide by filtration, the solvent was removed under reduced pressure. The residue was redissolved in CH_2Cl_2 (200 mL) and washed with H_2O (2 × 200 mL). The organic phase was dried over Na_2SO_4, filtrated and the solvent removed under reduced pressure to give product **2** as a peach-coloured solid (19.6 g, 109 mmol) in 56% yield.

^{1}H-NMR ($CDCl_3$, 400 MHz, 298 K): 4.09 (s, 2H), 3.24 (d, J = 12, 4H), 3.01 (d, J = 16, 4H), 0.86 (s, 6 H).

^{13}C{^{1}H}-NMR ($CDCl_3$, 101 MHz, 298 K): 211.7, 73.1, 65.9, 46.7, 16.4.

HR-ESI-MS: 181.1339 (100%, $C_{10}H_{17}N_2O^+$; $[M+H]^+$; calc. 181.1335).

Synthesis of compound 3

In a round-bottom flask (250 mL) compound **2** (11.3 g, 62.8 mmol, 1.0 eq.) was dissolved in acetic anhydride (59.0 mL, 628 mmol, 10.0 eq.) and stirred at room temperature for 2.5 h. After the addition of H_2O (30 mL), the mixture was stirred for another 10 minutes and the solvent removed under reduced pressure. To the residue, H_2O (36 mL) was added, and the precipitate which formed was filtered off and washed with H_2O. Redissolving the solid in $CHCl_3$ (200 mL), drying over Na_2SO_4 and removal of the solvent under reduced pressure gave compound **3** (8.78 g, 34.8 mmol) in 55% yield.

^{1}H-NMR ($CDCl_3$, 400 MHz, 298 K): 5.05 (dd, *J* = 8, 2, 2H), 4.08 (dd, *J* = 8, 2, 2H), 3.29 (dd, *J* = 8, 2, 2H), 2.76 (dd, *J* = 8, 2, 2H), 2.16 (s, 6H), 1.04 (s, 6H).

^{13}C{^{1}H}-NMR ($CDCl_3$, 101 MHz, 298 K): 211.9, 170.2, 58.0, 53.4, 46.1, 21.7, 16.7.

HR-ESI-MS: 253.1546 (100%, $C_{14}H_{21}N_2O_3^+$; $[M+H]^+$; calc. 253.1547).

Synthesis of compound 4

In a round-bottom flask (250 mL) equipped with a reflux condenser, compound **3** (6.57 g, 26.0 mmol, 1.0 eq.) was dissolved in HCl(aq.) (5 M, 45 mL, 12.5 eq.) and heated under reflux for 14 h. After cooling to room temperature, NaOH(aq.) (12 M, approx. 20 mL) was added to the mixture until the pH measured 14. Extraction with $CHCl_3$ (5 × 100 mL) yielded, after drying and removal of solvent under reduced pressure gave compound **4** (4.00 g, 23.8 mmol) as a white solid in 93% yield.

^{1}H-NMR ($CDCl_3$, 400 MHz, 298 K): 3.40 (d, *J* = 12, 4H), 2.96 (d, *J* = 8, 4H), 0.89 (s, 6H).

$^{13}C\{^1H\}$-NMR ($CDCl_3$, 101 MHz, 298 K): 61.5, 49.2, 17.3.
HR-ESI-MS: 169.1341 (100%, $C_9H_{17}N_2O^+$; $[M+H]^+$; calc. 169.1341).

Synthesis of compound 5

In a round-bottom flask (250 mL) **4** (4.00 g, 23.8 mmol, 1.0 eq.) was dissolved in dry EtOH (100 mL). $NaBH_4$ (1.08 g, 28.5 mmol, 1.2 eq.) was added in multiple portions and the resulting mixture was stirred at room temperature for 4 h. After removal of the solvent in vacuo, H_2O was added and again removed in vacuo. Solid-phase extraction was performed on the residue, by suspending it in refluxing $CHCl_3$, and filtering it while hot. Removal of solvent yielded the product (5, 3.71 g, 21.8 mmol) as a white solid in 92% yield. The product always contained impurities and was used for subsequent reactions without further purification.
^1H-NMR ($CDCl_3$, 400 MHz, 298 K): 3.44 (d, J = 12, 2H), 3.16 (d, J = 12, 2H), 2.99 (d, J = 12,), 2.82 (d, J = 12, 2H);0.97 (s, 6H).
$^{13}C\{^1H\}$-NMR ($CDCl_3$, 101 MHz, 298 K): 60.7, 49.9, 36.0, 21.2.
HR-ESI-MS: 171.1489 (100%, $C_9H_{19}N_2O^+$; $[M+H]^+$; calc. 171.1492).

Synthesis of compound 6

In a two-necked round-bottom flask (250 mL) equipped with a reflux condenser, compound **5** (3.65 g, 21.4 mmol, 1.0 eq.) and Cs_2CO_3 (41.9 g, 129 mmol, 6.0 eq.) was suspended in MeCN (80 mL) and heated to 50 °C. After addition of 2-(Boc-amino)ethyl bromide (10.6 g, 47.1 mmol, 2.2 eq.) the reaction mixture was stirred under reflux for 3 h. The solids were filtered off and the solvent removed under reduced pressure. The residue was dissolved in CH_2Cl_2 (150 mL) and filtered again. After removal of the solvent under reduced pressure, the colourless viscous liquid was purified by reverse-phase column chromatography to give compound **6** (6, 7.17 g, 15.7 mmol) as a colourless liquid in 73% yield.
^1H-NMR (CD_3CN, 400 MHz, 298 K): 6.52 (d, J = 48, 2H), 3.40 (q, J = 4, 2H), 3.24 (q, J = 4, 2H), 3.18 (s, 1 H), 2.92 (dd, J = 48, 6, 8H), 2.48 (t, J = 6, 2H), 2.27 (d, J = 12, 2H), 1.41 (d, J = 4, 18H), 0.87 (s, 6H).
$^{13}C\{^1H\}$-NMR (CD_3CN, 101 MHz, 298 K): 167.0, 80.2, 74.8, 64.3, 57.2, 38.9, 37.2, 28.7, 20.4.
HR-ESI-MS: 457.3369 (100%, $C_{23}H_{45}N_4O_5^+$; $[M+H]^+$; calc. 457.3385).

Synthesis of compound 8

In a round-bottom flask (500 mL) compound **6** (6.46 g, 14.1 mmol, 1.0 eq.) was dissolved in dry MeCN (250 mL). Under a constant nitrogen flow, potassium *tert*-butoxide (1 M in THF, 28.3 mL, 28.3 mmol, 2.0 eq.) was added slowly at 0 °C *via* syringe. The reaction mixture was stirred at 0 °C for 1 h, after which bromoacetic acid methyl ester (2.94 mL, 31.1 mmol, 2.2 eq.) and sodium iodide (45.0 mg, 2 mol%) were added. The solution was then allowed to warm to room temperature and stirred for 18 h. The reaction was then quenched by the addition of H_2O and extracted with CH_2Cl_2 (3 × 100 mL). After drying the combined organic phase and removal of solvent under reduced pressure, the crude product was

purified by reverse-phase column chromatography to give compound **7** (1.66 g, 3.15 mmol) as a white solid. The product still contained impurities of starting the material and was used for the next reaction without further purification. In a round-bottom flask (250 mL) compound **7** (1.56 g, 2.95 mmol, 1.0 eq) was dissolved in THF (80 mL) and MeOH (40 mL). Sodium hydroxide (1.42 g, 35.40 mmol, 12.0 eq.) was dissolved in H_2O (20 mL) and added to the reaction mixture. After stirring at room temperature for 14 h, the pH of the mixture was adjusted to 6 by the addition of HCl(aq.) (5 M). The solvent was removed under reduced pressure and the crude product purified by reverse-phase column chromatography to give compound **8** (562 mg, 1.09 mmol) as a white solid in 37% yield over two steps.

^{1}H-NMR ($CDCl_3$, 400 MHz, 298 K): 6.70 (s, 1H); 6.43 (s, 1H); 4.18 (s, 2H); 3.59 (dd, J = 12, 6, 2H); 3.33-3.30 (m, 8H); 3.10 (d, J = 12, 2H); 2.94 (s, 1H); 2.53 (m, 2H); 2.19 (d, J = 8, 2H); 1.43 (s, 18H); 0.97 (s, 6H).

^{13}C{^{1}H}-NMR ($CDCl_3$, 101 MHz, 298 K): 174.2, 162.6, 156.7, 84.5, 79.5, 78.8, 75.2, 63.9, 56.3, 55.6, 55.3, 38.5, 37.2, 36.5, 35.8, 28.7, 28.5, 20.4.

HR-ESI-MS: 515.3469 (100%, $C_{25}H_{47}N_4O_7^+$; $[M+H]^+$; calc. 515.3439).

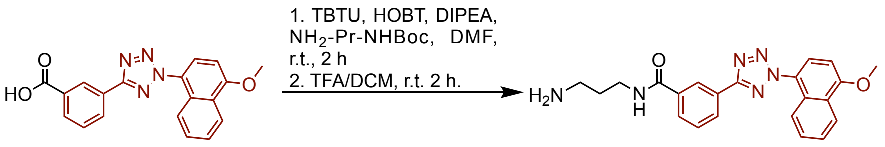

Figure S1. Synthesis of Tetrazole-propyl-NH_2 (**9**) from Tz-CO_2H.

Synthesis of compound 9

The tetrazole-carboxylate (Tz-CO_2H) starting material was synthesised in accordance with previously reported methods.[10] In a round bottom flask, tetrazole-carboxylate (191 mg, 547 µmol. 1.0 eq.) was dissolved in DMF (20 mL). 2-(1*H*-Benzotriazole-1-yl)-1,1,3,3-tetramethylaminium tetrafluoroborate (TBTU, 67 mg, 520 µmol, 0.95 eq.) and 1-Hydroxybenzotriazol (HOBT, 70.2 mg, 520 µmol, 0.95 eq.) were added and the reaction basified with DIPEA (189 µL, 1.09 mmol, 2.0 eq.). After stirring for 10 min at r.t., the reaction mixture was added to *N*-Boc-1,3-propanediamine (2.72 mL, 32.5 mmol, 2 eq.) during 1 h and the reaction stirred for another 2 h. The solvent was removed under reduced pressure, dichloromethane (30 mL) added, and the organic phase washed with water (2 × 50 mL). The organic phase was collected, dried over Na_2SO_4, solids filtrated, and the volume concentrated to 20 mL. Trifluoroacetic acid (5 mL) was added and the reaction stirred for 2 h at r.t. Then, the solvent was removed under reduced pressure and the crude product purified by using column chromatography (C_{18}-SiO_2, gradient from 100% H_2O (0.1% FA) to 100% MeCN). Tz-propyl-NH_2 **9** was obtained as yellow oil in 55% yield (121.6 mg, 302 µmol).

^{1}H-NMR (CD_3CN, 400 MHz, 298 K): δ 8.64 (td, *J* = 1.8, 0.6 Hz, 1H), 8.38 – 8.32 (m, 2H), 8.00 – 7.97 (m, 2H), 7.81 – 7.79 (m, 2H), 7.65 – 7.60 (m, 3H), 7.05 (d, *J* = 8.3 Hz, 1H), 4.08 (s, 3H), 3.51 (dt, *J* = 7.4, 6.2 Hz, 2H), 3.04 (t, *J* = 6.6 Hz, 2H), 1.98 (dd, *J* = 19.2, 6.6 Hz, 2H).

^{13}C{^{1}H}-NMR (CD_3CN, 101 MHz, 298 K): 168.9, 165.3, 158.5, 135.8, 130.7, 130.5, 130. 2, 129. 6, 129.2, 128.7, 127.6, 127.2, 126.5, 126.5, 125.8, 123.3, 123.1, 104.2, 56.9, 37.9, 36.8, 28.0.

HR-ESI-MS: 403.1885 (100%, $C_{22}H_{23}N_6O_2^+$; $[M+H]^+$; calc. 403.1877).

Synthesis of compound 10

In a round bottom flask, compound **8** (140 mg, 272 µmol, 1.1 eq.) was dissolved in DMF (40 mL). EDC HCl (47.3 mg, 247 µmol, 1.0 eq.) and HOBt (33.4 mg, 247 µmol, 1.2 eq.) were added and the reaction basified with DIPEA (95 µL, 544 µmol, 2.0 eq.). After stirring for 10 min at r.t., Tetrazole-amine **9** (99 mg, 247 µmol, 1.0 eq.) was added and the reaction stirred for another 24 h. After successful coupling was confirmed by means of LC-MS, the solvents were removed under reduced pressure. The crude product was dissolved in dichloromethane (20 mL), trifluoroacetic acid (5 mL) added, and the reaction mixture heated to 50 °C. After stirring for 2 h, the solvent was evaporated and the crude product purified by means of reverse phase column chromatography (C_{18}-SiO_2, gradient from 100% H_2O (+ 0.1% FA) to 100% MeCN). Compound **10** was obtained as white solid in 10% yield (17.6 mg, 24.7 µmol).

^{1}H-NMR (D$_2$O, 400 MHz, 298 K): δ 8.01 (s, 1H), 7.65 – 7.61 (m, 4H), 7.35 (d, *J* = 7.7 Hz, 1H), 7.20 (d, *J* = 8.6 Hz, 1H), 7.12 (d, *J* = 7.9 Hz, 1H), 6.99 (s, 3H), 6.19 (s, 1H), 4.07 (s, 2H), 3.57 – 3.55 (m, 4H), 3.34 – 2.97 (m, 15H), 2.67 (t, *J* = 6.4 Hz, 2H), 2.22 (d, *J* = 11.9 Hz, 2H), 1.77 (s, 3H), 1.28 (t, *J* = 7.4 Hz, 4H), 0.82 (s, 6H).

^{13}C{^{1}H}-NMR (D$_2$O, 101 MHz, 298 K): δ 171.1, 170.7, 168.0, 163.30, 163.2, 159.0, 156.5, 133.9, 129.2, 128.9, 126.6, 126.1, 125.0, 124.6, 121.8, 121.4, 117.8, 84. 0, 83.2, 72.7, 62.4, 56.1, 55.5, 53.5, 46.7, 36.3, 35.3, 34.1, 28.4, 18.9, 8.2.

HR-ESI-MS: compound (Boc)$_2$-10: 899.5143 (100%, $C_{47}H_{67}N_{10}O_8^+$; $[M+H]^+$; calc. 899.5138).

HR-ESI-MS: compound **10**, 699.4083 (100%, $C_{37}H_{51}N_{10}O_4^+$; $[M+H]^+$; calc. 699.4089).

Synthesis of compound 11

To a solution of compound **10** (17.9 mg, 25.6 µmol, 1.0 eq.) in 20 mL MeCN was added 65.8 mg of 1,8-*bis*(dimethylamino)naphthalene (Proton Sponge™, 307 µmol, 12 eq.) and the reaction was stirred at 50 °C for 30 min. Then, bromoacetic acid methyl ester (78.3 µL, 512 µmol, 20 eq.) was added and the reaction was stirred under reflux for 16 h. The solvent was evaporated, the crude mixture dissolved in dichloromethane (20 mL) and the organic phase washed with water (3 × 20 mL). The organic phase was then dried over Na_2SO_4, filtrated and the solvent evaporated. The crude product was purified using reverse phase column chromatography (C_{18}-SiO_2, gradient from 100% H_2O (0.1% FA) to 100% MeCN). Compound **11** was obtained as yellow oil in 46% yield (11.5 mg, 11.7 µmol).

^{1}H-NMR (DMSO-d6, 400 MHz, 298 K): 8.69 (dd, *J* = 4.1, 2.1 Hz, 1H), 8.37 – 8.35 (m, 2H), 8.08 (d, *J* = 7.9 Hz, 1H), 8.00 (d, *J* = 8.3 Hz, 1H), 7.99 (m, 1H), 7.75 – 7.70 (m, 4H), 7.69 (t, *J* = 6.0 Hz, 2H), 7.23 (d, *J* = 8.4 Hz, 2H), 4.12 (s, 4H), 4.03 – 4.00 (m, 2H), 3.73 (t, *J* = 6.4 Hz, 2H), 3.60 – 3.46 (m, 6H), 3.36 (dq, *J* = 12.3, 6.3 Hz, 5H), 3.33 – 3.15 (m, 9H), 2.85 (dq, *J* = 20.6, 6.6, 4.8 Hz, 2H), 2.84 – 2.71 (m, 4H), 2.70 (dt, *J* = 10.4, 2.4 Hz, 2H), 2.46 – 2.13 (m, 4H), 2.00 (dtd, *J* = 29.3, 11.3, 3.2 Hz, 2H), 1.70 (p, *J* = 6.2 Hz, 2H), 1.37 (s, 3H), 1.06 (d, *J* = 14.1 Hz, 2H), 0.95 (m, 3H).

^{13}C{^{1}H}-NMR (DMSO-d6, 101 MHz, 298 K): δ 172.0, 168.5, 165.6, 164.0, 157.0, 151.0, 145.6, 135.5, 129.5, 129.5, 129.2, 128.9, 128.0, 126.9, 126.8, 125.7, 125.4, 125.2, 124.9, 122.3, 122.2, 121.9, 121.8, 103.7, 88.6, 88.0, 77.6, 73.3, 65.34, 63.45, 56.4, 56.0, 55.8, 54.4, 45.2, 44.7, 44.1, 36.7, 36.5, 35.7, 35.2, 29.4, 29.0, 28.2, 21.1, 20.9, 18.6, 18.5, 18.2.

HR-ESI-MS: 987.4947 (100%, $C_{49}H_{67}N_{10}O_{12}^{+}$; $[M+H]^{+}$; calc. 987.4934).

Synthesis of compound 12

Deprotection to final compound **12** was achieved by treatment of compound **11** (16.8 mg, 17.0 µmol, 1.0 eq.) with sodium hydroxide (10 wt%, 65 µL, 80.8 µmol, 20 eq.) in MeOH/THF/water (2:1:1, 2 mL). After stirring at r.t. for 16 h, the pH was adjusted to pH3 using HCl(aq.) (1 M), the solvents were evaporated under reduced pressure and the crude product was purified by means of reverse phase column chromatography (C18-SiO_2, gradient from 100% H_2O (0.1% FA) to 100% MeCN). Bisp-Tz **12** was obtained as an off-white solid in 66% yield (6.2 mg, 10.1 µmol) and >95% purity, as confirmed by HPLC.

^{1}H-NMR (D_2O, 400 MHz, 298 K): δ 8.61 (s, 1H), 8.38 (s, 2H), 7.99 (s, 1H), 7.87 (s, 1H), 7.78 – 7.66 (m, 4H), 7.13 (s, 1H), 3.41 (s, 2H), 3.32 (s, 2H), 3.21 (s, 4H), 3.15 (m, 8H), 2.90 (s, 6H), 2.76 (s, 2H), 2.48 (s, 2H), 2.25 (s, 2H), 1.79 (s, 4H), 1.24 (s, 2H), 0.90 (s, 6H).

HR-ESI-MS: 931.4305 (100%, $C_{45}H_{59}N_{10}O_{12}^{+}$; $[M+H]^{+}$; calc. 931.4308).

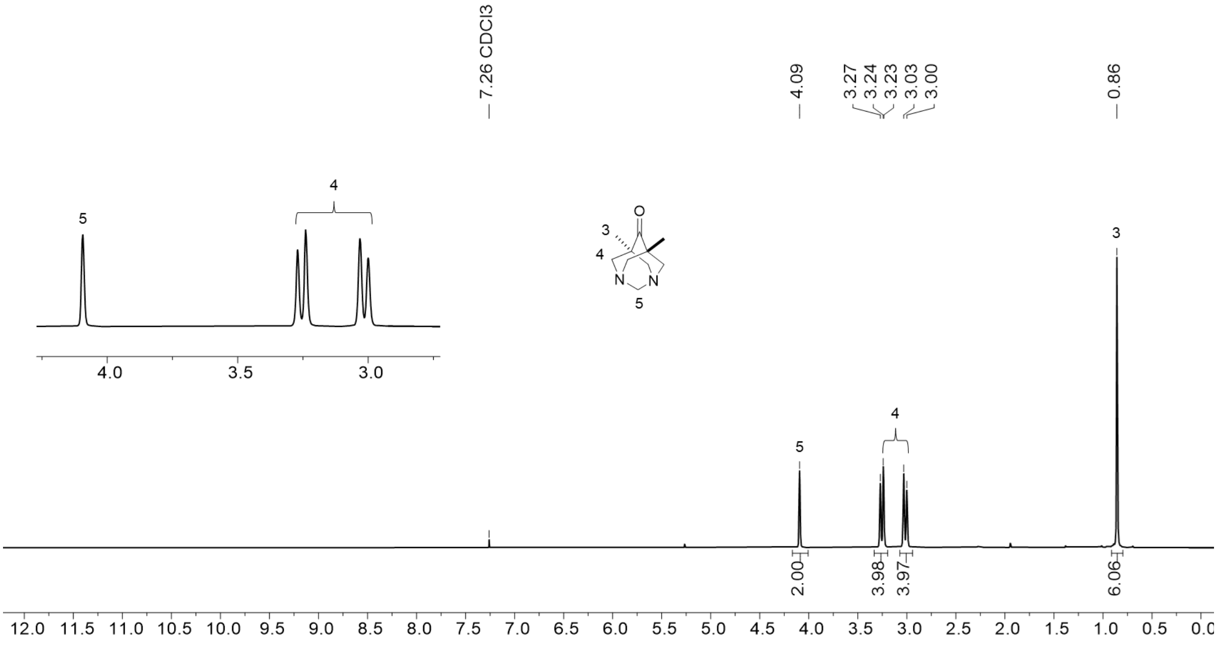

Figure S2. ^{1}H-NMR spectrum of compound **2** ($CDCl_3$, 400 MHz).

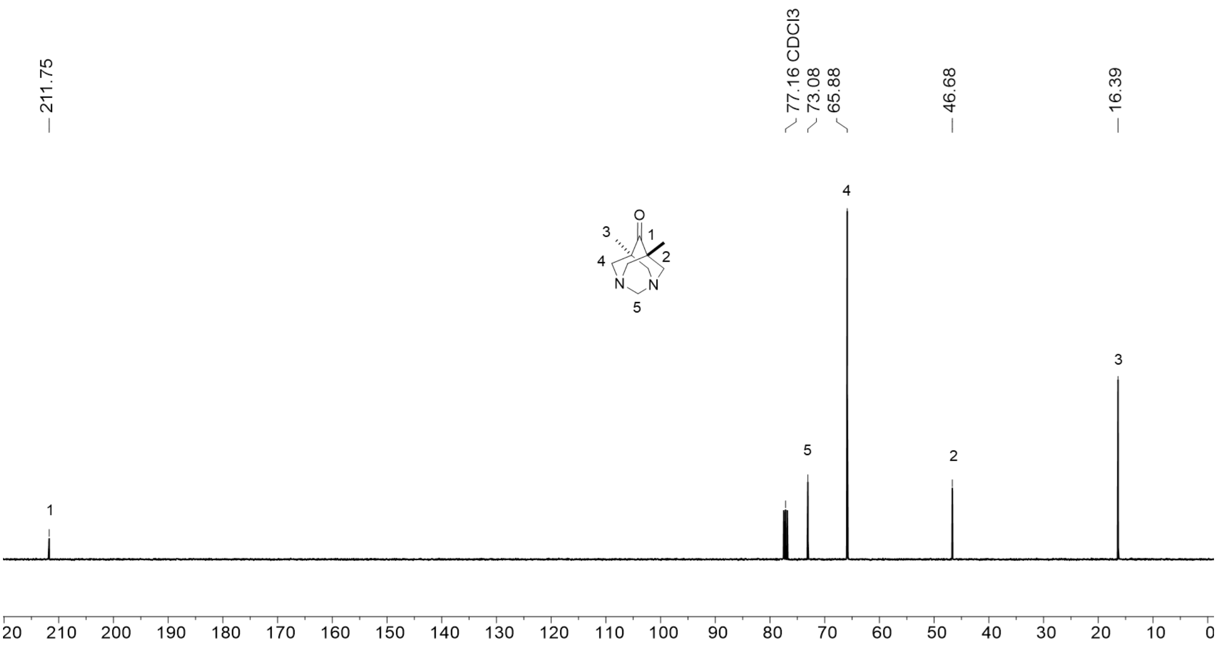

Figure S3. $^{13}C\{^{1}H\}$-NMR spectrum of compound **2** ($CDCl_3$, 101 MHz).

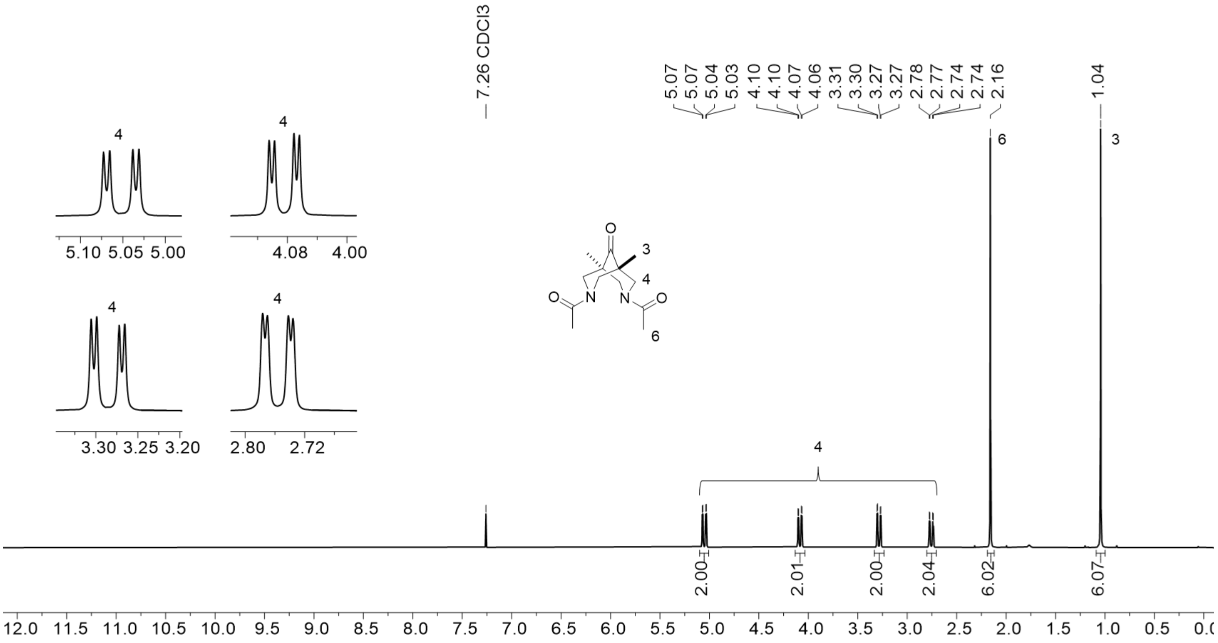

Figure S4. ^{1}H-NMR spectrum of compound **3** ($CDCl_3$, 400 MHz).

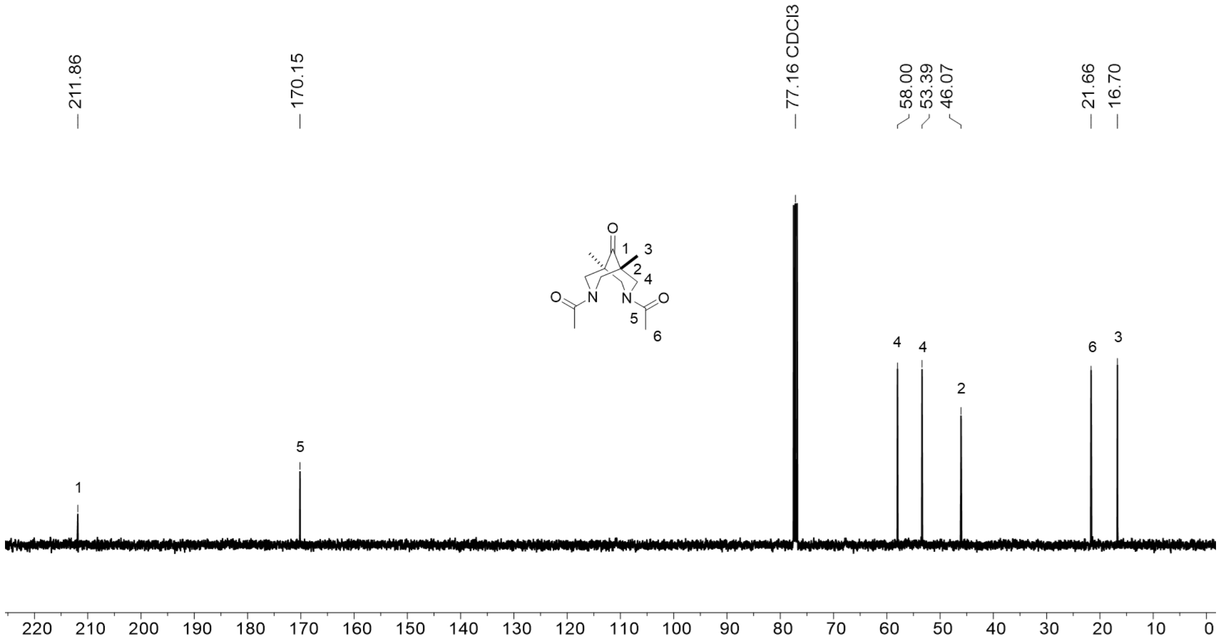

Figure S5. $^{13}C\{^{1}H\}$-NMR spectrum of compound **3** ($CDCl_3$, 101 MHz).

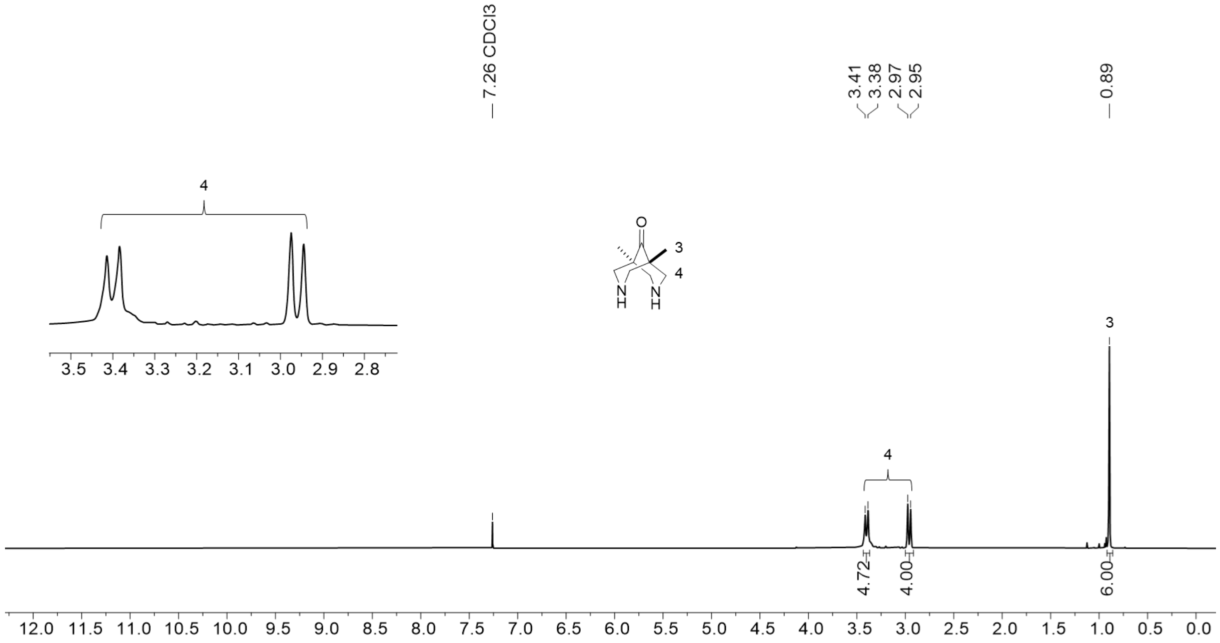

Figure S6. ^{1}H-NMR spectrum of compound **4** ($CDCl_3$, 400 MHz).

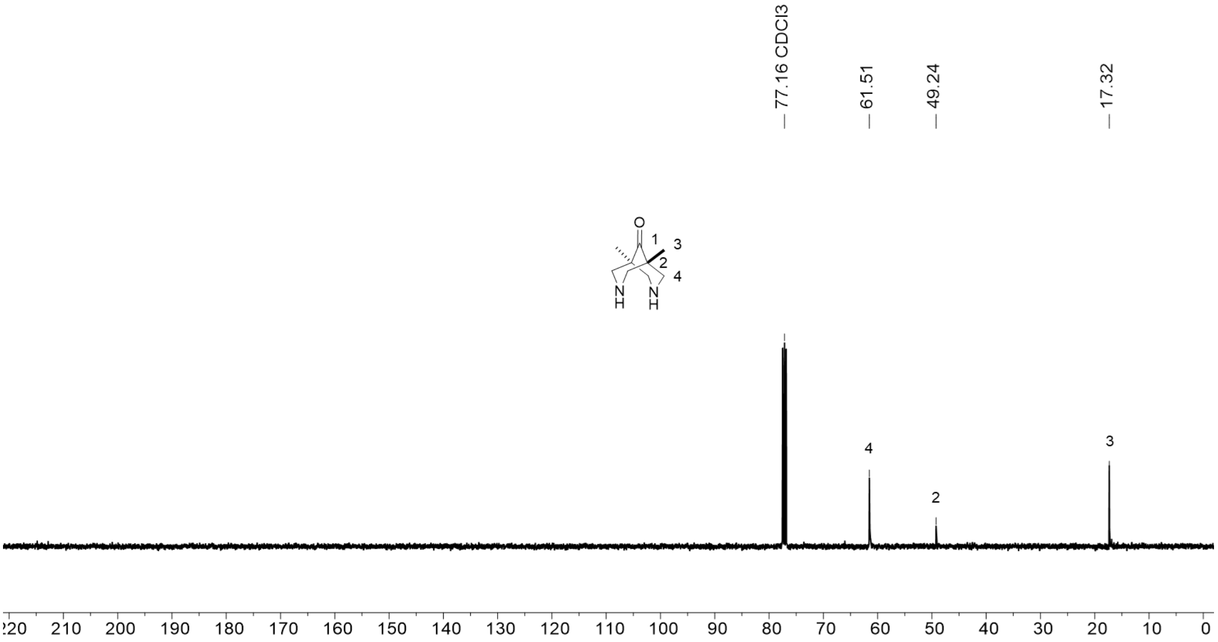

Figure S7. $^{13}C\{^{1}H\}$-NMR spectrum of compound **4** ($CDCl_3$, 101 MHz).

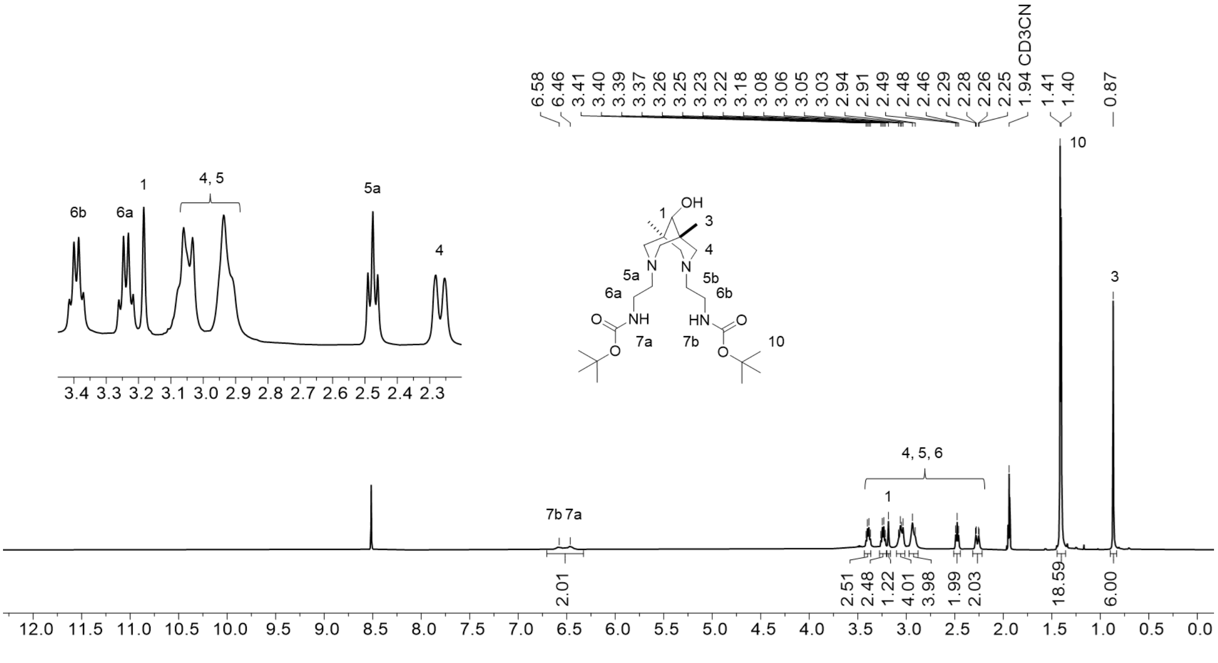

Figure S8. ^{1}H-NMR spectrum of compound **6** (CD_3CN, 400 MHz).

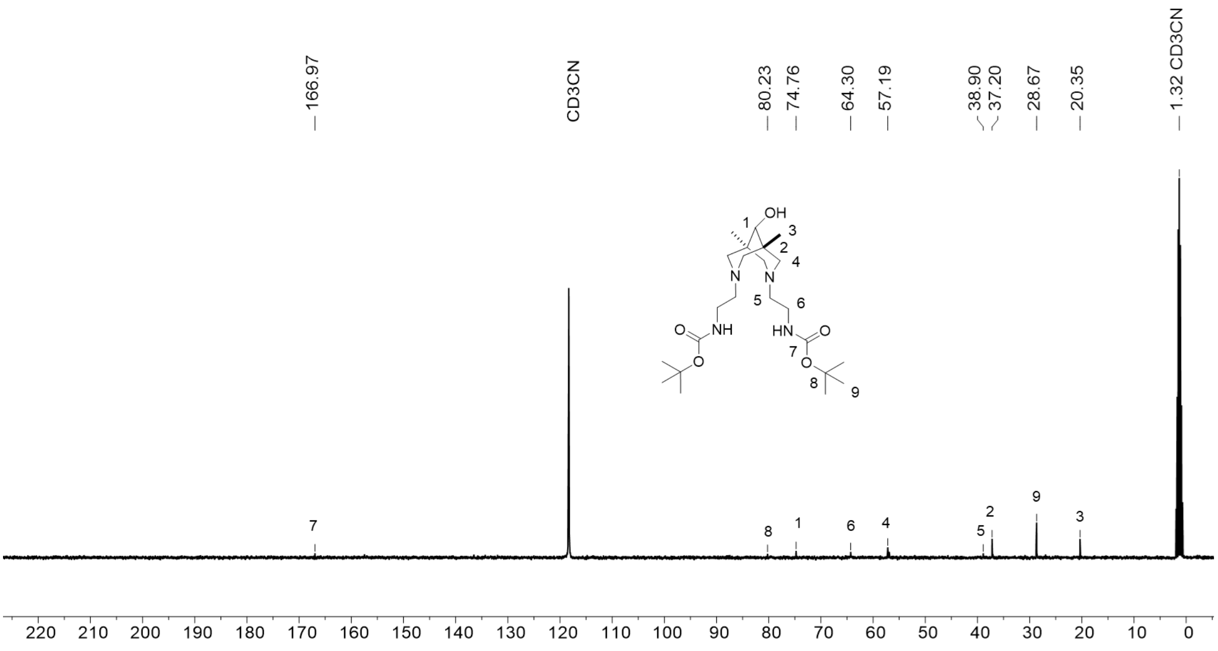

Figure S9. $^{13}C\{^1H\}$-NMR spectrum of compound **6** (CD_3CN, 101 MHz).

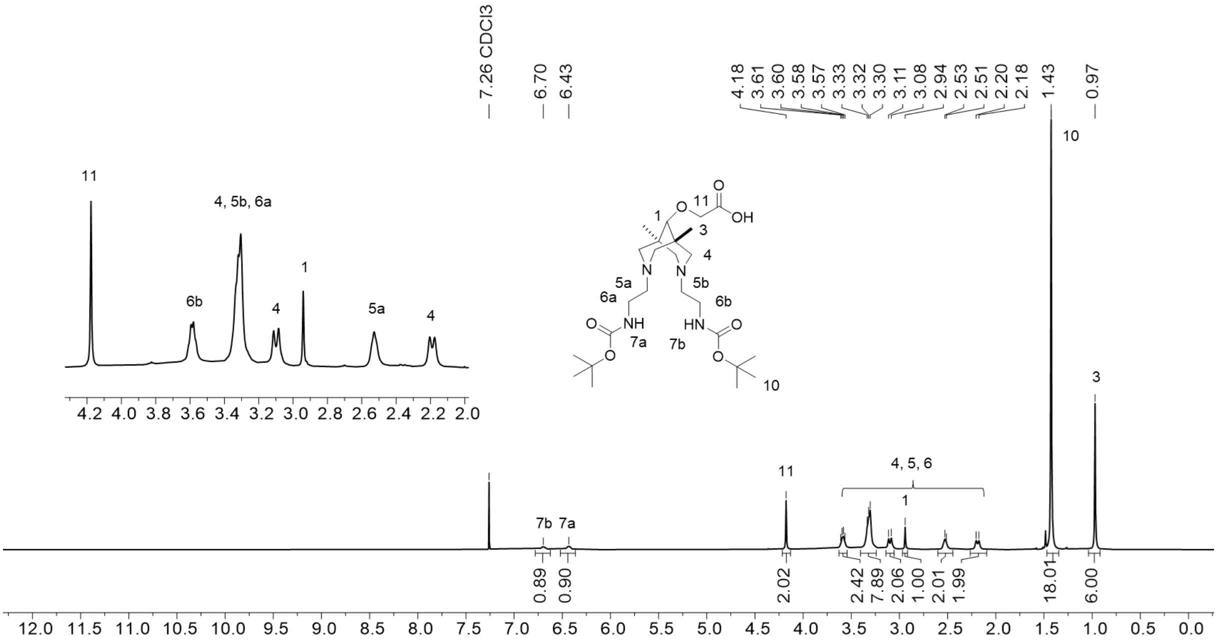

Figure S10. ^1H-NMR spectrum of compound **8** ($CDCl_3$, 400 MHz).

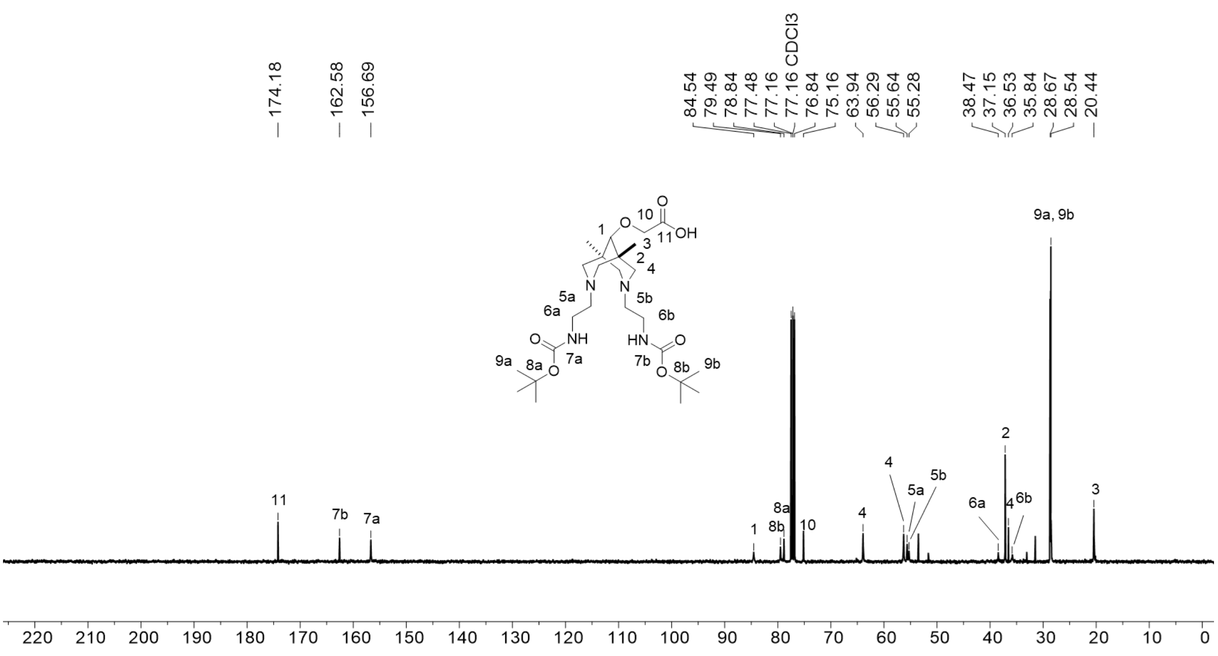

Figure S11. $^{13}C\{^1H\}$-NMR spectrum of compound **8** ($CDCl_3$, 101 MHz).

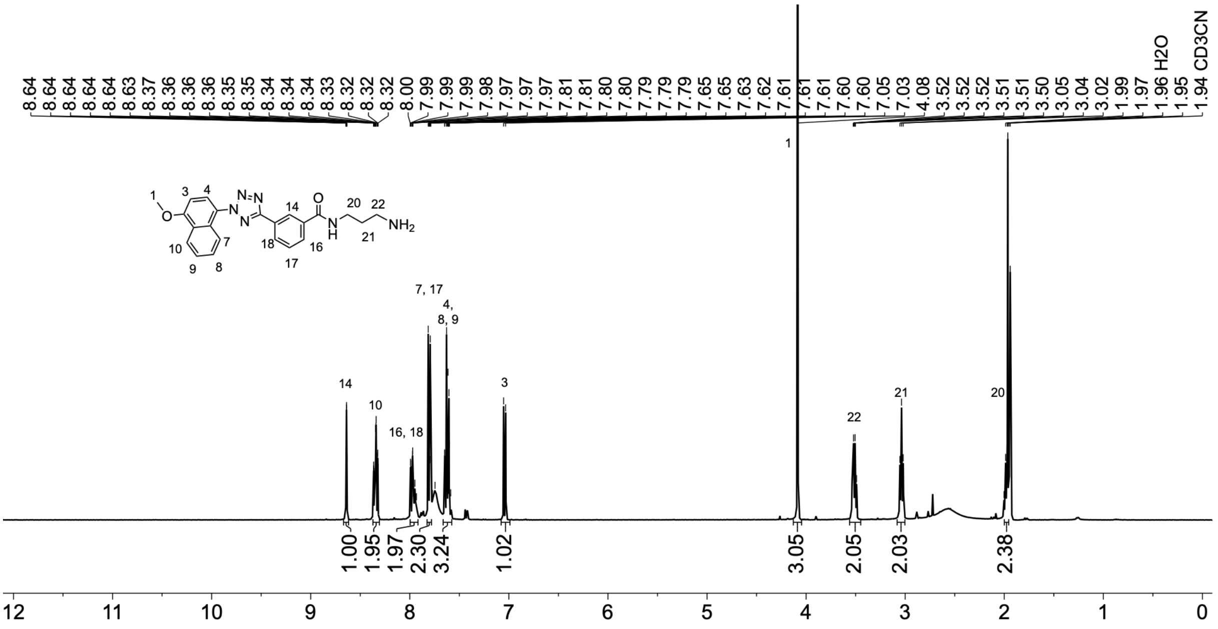

Figure S12. ^1H-NMR spectrum of compound **9** (CD_3CN, 400 MHz).

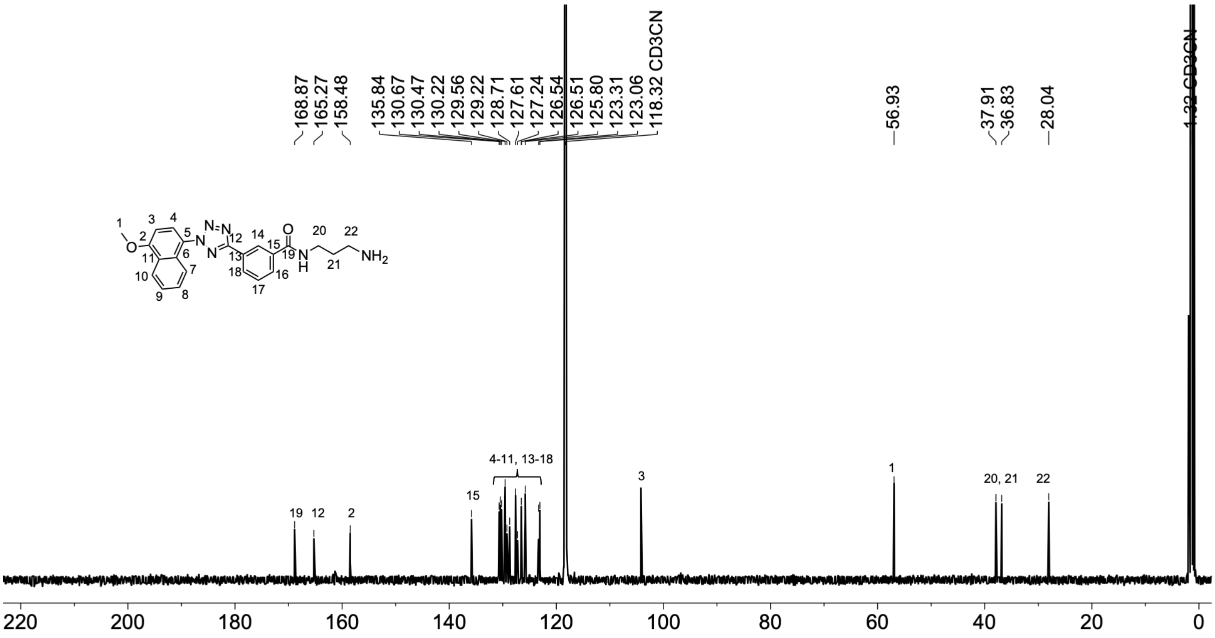

Figure S13. $^{13}C\{^{1}H\}$-NMR spectrum of compound **9** (CD_3CN, 101 MHz).

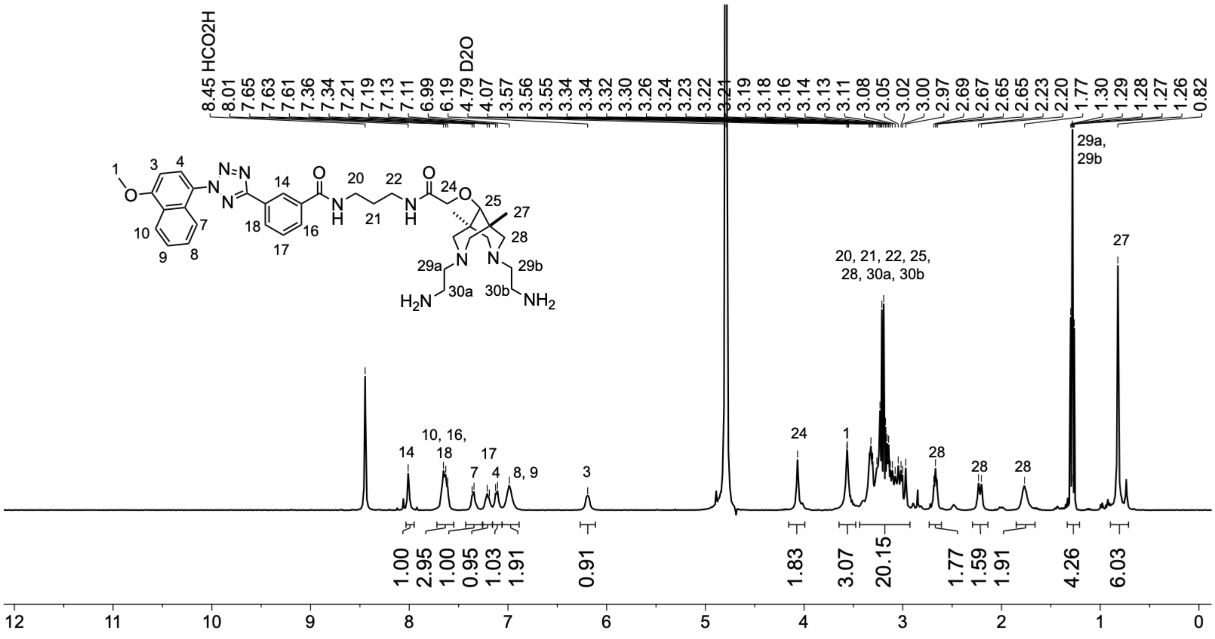

Figure S14. ^{1}H-NMR spectrum of compound **10** (D_2O, 400 MHz).

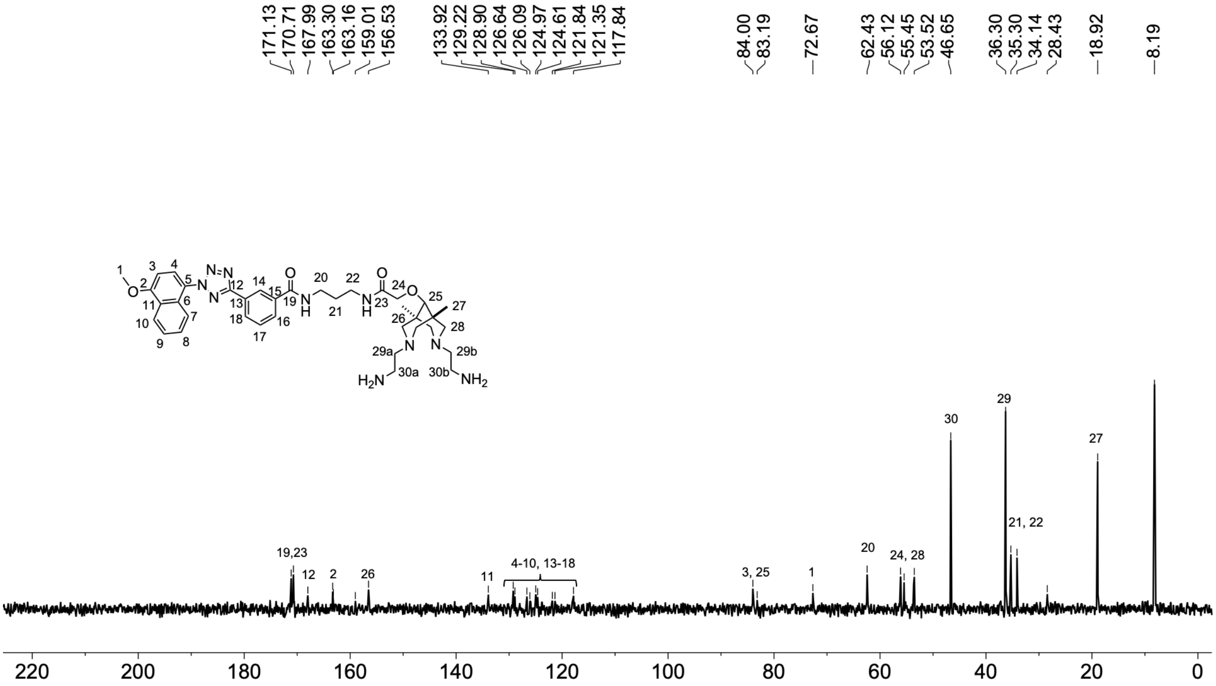

Figure S15. $^{13}C\{^{1}H\}$-NMR spectrum of compound **10** (D_2O, 101 MHz).

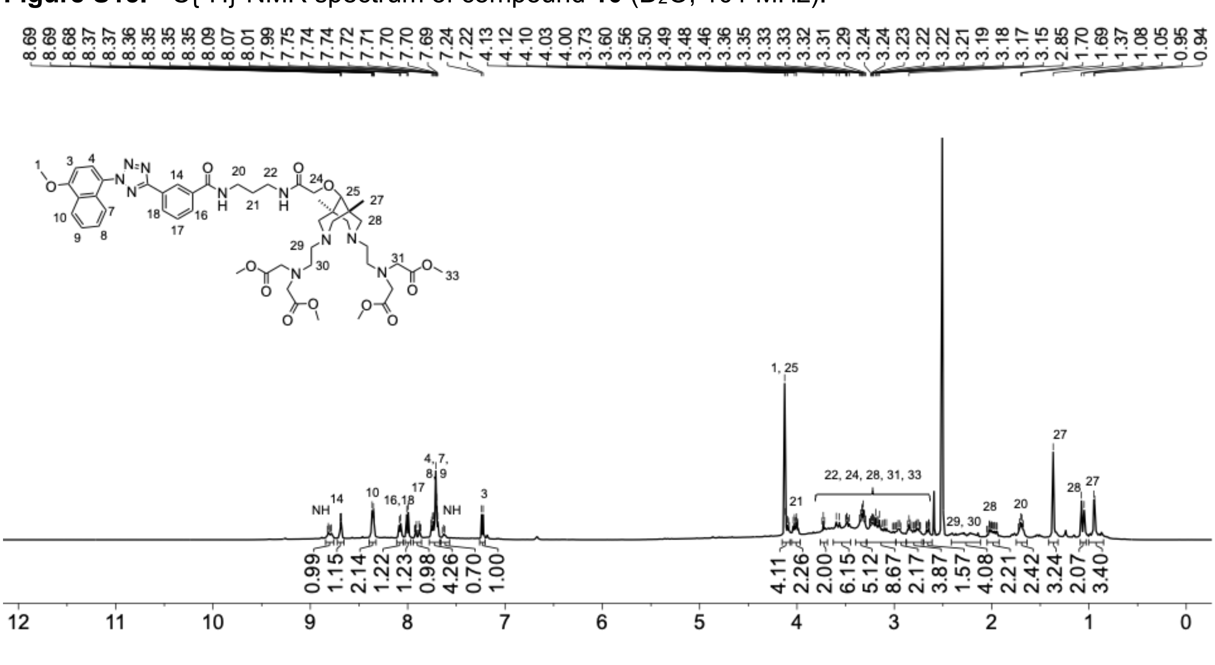

Figure S16. ^{1}H-NMR spectrum of compound **11** (DMSO-d6, 400 MHz).

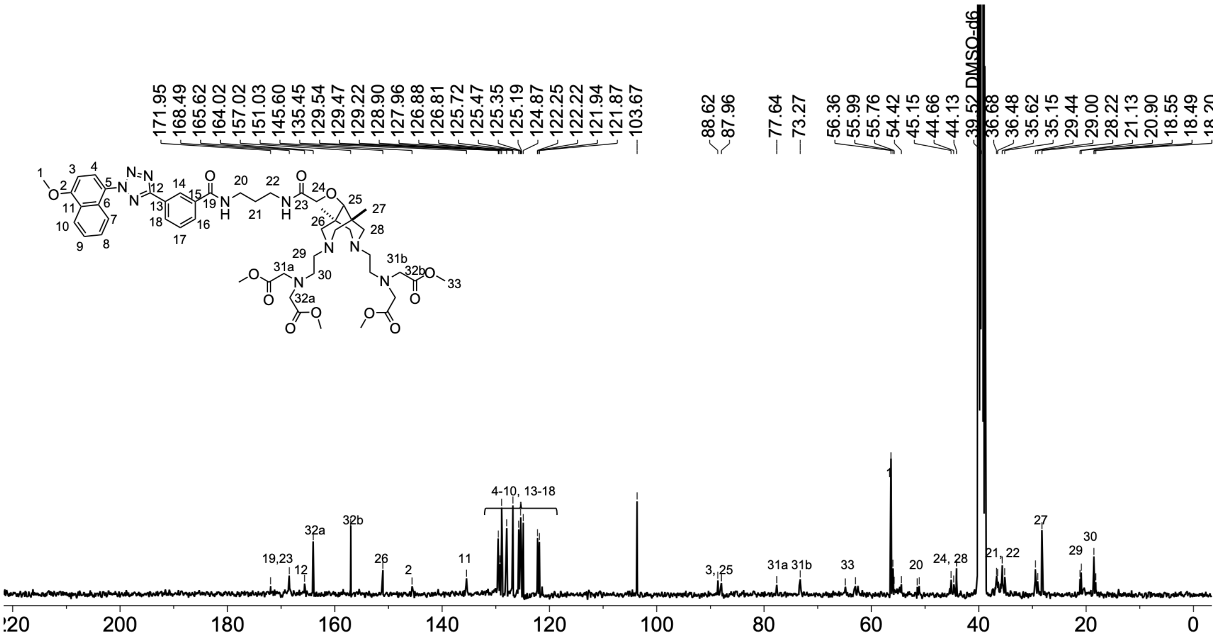

Figure S17. $^{13}C\{^{1}H\}$-NMR spectrum of compound **11** (DMSO-d6, 101 MHz).

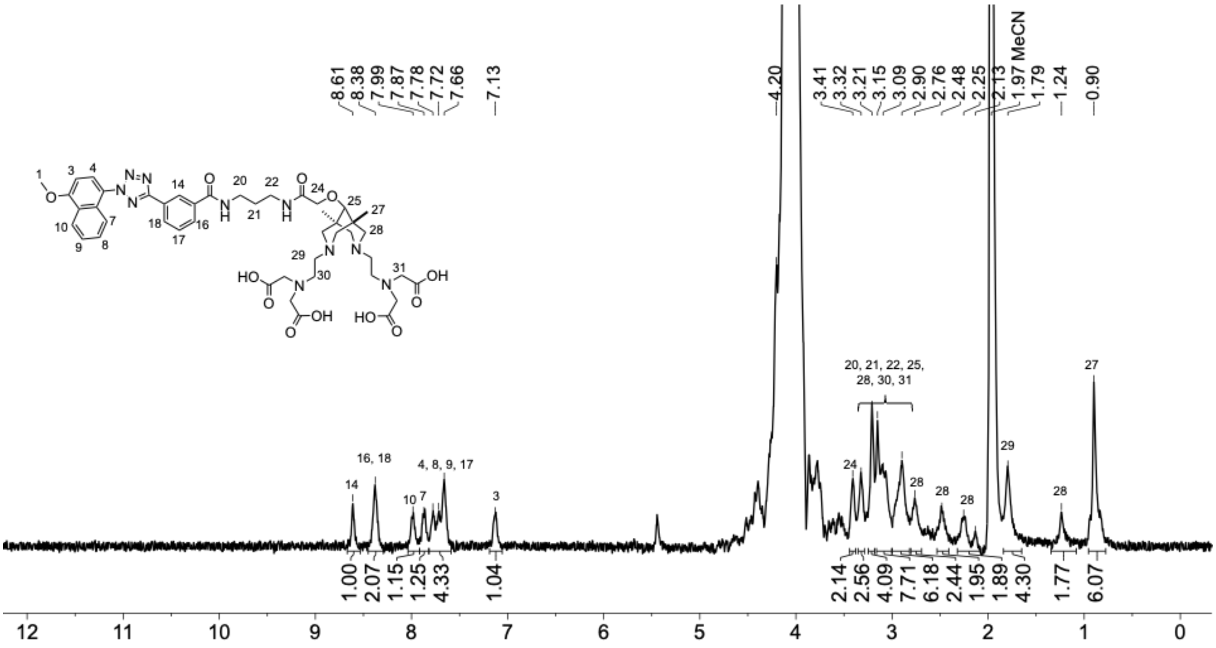

Figure S18. ^{1}H-NMR spectrum of compound **12** (D_2O, 400 MHz).

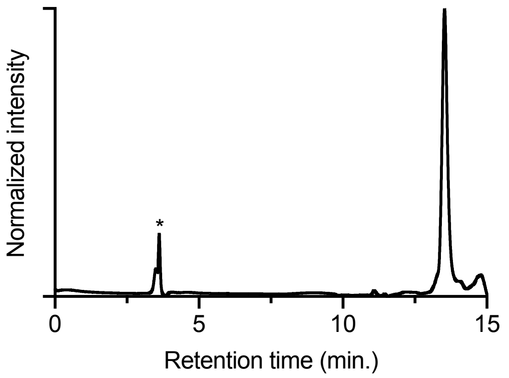

Figure S19. Reverse-phase analytical HPLC chromatogram of **12**.
The asterisk shows residual MeCN from the stock solution.

Synthesis and characterisation of ^{nat}Tb-12$^{–}$

To a solution of **12** (50 µL, 1 mM) in NaOAc (50 µL, 0.1 M, pH 5.5) was added a solution of $TbCl_3$ (1.2 µL, 10 mM, 1.2 eq) and the reaction was heated to 40 °C for 5 min. The resulting solution containing the complex ^{nat}Tb-**12**$^{–}$ was analysed directly by using ESI-MS and HPLC.

HR-ESI-MS (+): 1087.3331 (100, $C_{45}H_{56}N_{10}O_{12}Tb^{+}$; $[M+2H]^{+}$; calc. 1087.3327).

HR-ESI-MS (-): 1085.31724 (100, $C_{45}H_{54}N_{10}O_{12}Tb^{-}$; $[M]^{-}$; calc. 1085.31816).

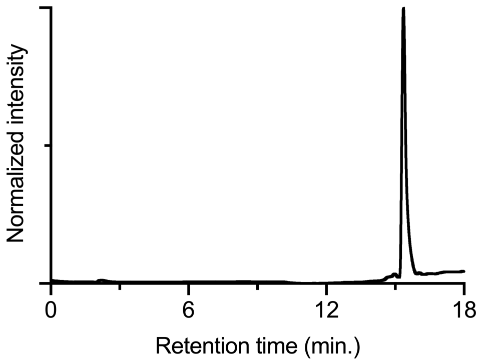

Figure S20. Reverse-phase analytical HPLC chromatogram of ^{nat}Tb-**12**$^{–}$.

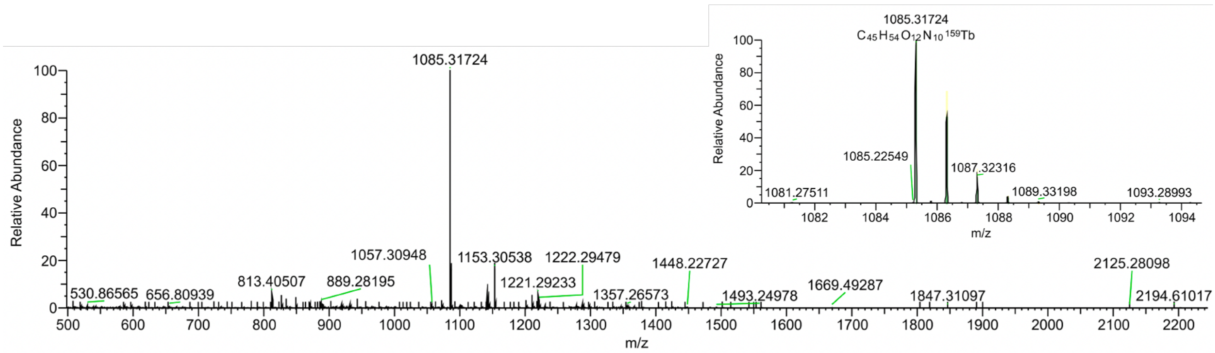

Figure S21. HRMS (ESI-) spectrum of ^{nat}Tb-**12**$^{–}$.

(Inset) Isotopic pattern of the base peak which matches the expected pattern.

Radiosynthesis of ^{161}Tb-12^{-}

Radiolabelling reactions to prepare ^{161}Tb-**12**$^{-}$ were accomplished by the addition of an aliquot of $^{161}TbCl_3$ stock solution (~1 MBq) to a solution of **12** (5 µL of 1 mM stock in H_2O) in NaOAc buffer (100 mM, pH5.5) with a total reaction volume of 100 µL. The reactions were monitored by radio-iTLC (100 mM aqueous Na_3(citrate) at pH 5.5) and complexation was found to be complete in less than 10 min. at 40 ºC giving a decay-corrected radiochemical conversion (RCC) >95% (R_f = 0.1 – 0.2). The product was characterised by analytical HPLC following the method described in the general methods section (*vide supra*). Note: the electronic absorption (UV/vis) detector and radioactivity detector were arranged serially with an offset time of approximately 0.10-0.30 min (depending on temperature). The identity of the ^{161}Tb-**12**$^{-}$ radiolabelled compound was confirmed by co-injection with an authenticated sample of non-radiolabelled complex ^{nat}Tb-**12**$^{-}$.

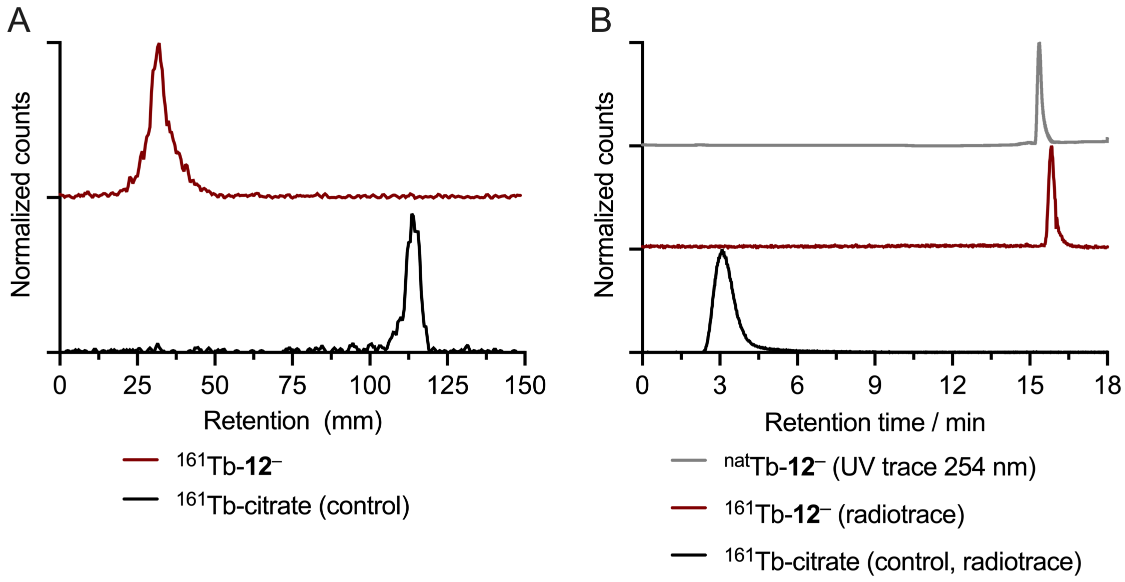

Figure S22. Chromatographic data on the characterisation of ^{161}Tb-**12**$^{-}$.
(A) Radio-TLC chromatograms of ^{161}Tb-**12**$^{-}$ (red) and ^{161}Tb(citrate) (black) eluting with aqueous disodium citrate(100 mM, pH5.5), and (B) HPLC chromatograms showing the radiotrace of ^{161}Tb-**12**$^{-}$ (red), the electronic absorption UV trace (254 nm) of the non-radioactive ^{nat}Tb-**12**$^{-}$ complex (grey), and the radiotrace of the control sample, ^{161}Tb(citrate) (black).

Photoradiosynthesis of ^{161}Tb-12-onartuzumab for biological experiments

To a vial containing NaOAc (2.5 M, pH5.5, 2 µL), the stock solution of compound **12** (10 µL, 1 mM, 10 nmol) and an aliquot of the stock solution of $^{161}TbCl_3$ (20.4 µL, 499.2 MBq, 0.05 M HCl) was added and the reaction stirred gently at 40 °C for 10 min. In parallel, Traut's reagent (2-iminothiolane, 2 µL, 10 mM in $NaHCO_3$ (100 mM, pH8), 20 nmol, 2.0 eq.) was added to an aliquot of the stock solution of onartuzumab (stock concentration = 60 mg mL^{-1}, MW(onartuzumab) = 99.16 kDa, volume added = 16.67 µL, protein mass = 1.00 mg, protein moles = 10 nmol) in 48.75 µL of $NaHCO_3$ buffer (100 mM, pH 8).

After 20 minutes an aliquot of the radioactive reaction was added to a solution of the antibody in $NaHCO_3$ buffer (0.1 M, pH8). The initial chelate-to-mAb ratio was 1-to-1. The reaction pH was measured and was between 7.8 – 8.1. The total reaction volume was ~100 µL giving a final [mAb] = [**12**] = 100 µM. The reaction was stirred gently at 23 °C and irradiated directly from the top of the vial for 5 min with 365 nm light. After the irradiation step, iodoacetamide (2 µL, 50 mM in NaHCO3 (100 mM, pH8), 100 nmol, 10.0 eq.) was added to the mixture to cap residual free thiols and the reaction stirred in the dark at r.t. for 30 min. Then, aliquots of the crude reaction mixtures were purified by preparative PD-10 SEC (collecting the 2.5 – 4.1 mL high molecular weight fraction using sterile PBS as an eluent). The combined fractions were transferred to a 30 kDa Millipore spin filtration falcon (5 mL) and concentrated at 4000 rpm for 25 min at 23 °C. Crude and purified aliquots were analysed by using analytical radio-iTLC, PD-10 SEC and SEC-HPLC. The isolated decay corrected radiochemical yield (RCY) of ^{161}Tb-**12**-onartuzumab was 23.5% and the lower limit of the molar activity of the product (estimated by assuming no protein losses) was ~11.2 MBq nmol^{-1} of protein, with an activity concentration of 362 MBq mL^{-1}. The radiochemical purity of the purified samples of ^{161}Tb-**12**-onartuzumab was estimated to be ~95% (measured by SEC-HPLC).

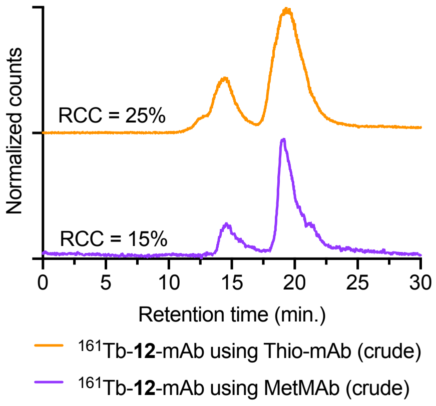

Figure S23. SEC-HPLC chromatograms showing the radiotrace of crude samples of ^{161}Tb-**12**$^{-}$ prepared by reaction with either fully formulated MetMAb (purple) or MetMAb previously treated with 2-iminothiolane (orange).

Animal studies and preparation of ^{161}Tb-12-onartuzumab doses for injection in mice

Dose for biodistribution studies

For the biodistribution study, stock solutions of the radiotracer were prepared by taking an aliquot of the purified and formulated sample of ^{161}Tb-**12**-onartuzumab (16.5 µL, 5.37 MBq, A_m = ~11.2 MBq nmol^{-1}) and added to a sterile vial before diluting with sterile PBS to a final volume of 1.0 mL, and drawing syringes (150 µL/syringe). The tail of each mouse was warmed gently by using a warm water bath (~37 °C) immediately before administering ^{161}Tb-**12**-onartuzumab. Two groups of animals were used whereby each mouse in the normal group (*n* = 5 mice) received a dose containing 0.736 – 0.851 MBq of activity (6.52 – 7.53 µg of protein, 0.066 – 0.076 nmol in 150 µL sterile PBS) *via* intravenous (i.v.) tail-vein

injection (t = 0 h). For the competitive inhibition (blocking) experiments, a separate aliquot of ^{161}Tb-**12**-onartuzumab (16.5 µL, 5.60 MBq) was added to a sterile vial. Then an aliquot of the stock solution of formulated onartuzumab (MetMAb, 60 mg mL^{-1}, 100 µL, 6.0 mg of protein), was added to reduce the molar activity (A_m(block) = ~0.092 MBq nmol^{-1}) and the mixture was diluted with sterile PBS to a final volume of 1.0 mL. Animals in the blocking group (n = 5 mice) each received an i.v. dose containing 0.841 – 0.951 MBq (906.5 – 1025.0 µg of protein/mouse, 9.14 – 10.34 nmol in 150 µL sterile PBS).

Doses for therapeutic studies

For the therapeutic study, an aliquot of the purified and formulated sample of ^{161}Tb-**12**-onartuzumab (121 µL, 43 MBq) was added to a sterile vial and diluted with sterile PBS to a final volume of 1.65 mL. The tail of each mouse was warmed gently by using a warm water bath (~37 °C) immediately before administering ^{161}Tb-**12**-onartuzumab. Two groups of animals were used whereby each mouse in the normal group (n = 10 mice) received a dose containing 3.17 – 3.68 MBq of activity (mean activity = 3.50±0.134 MBq, equivalent to 112.9 MBq mg^{-1}, 28.1 – 32.6 µg of protein, 0.283 – 0.328 nmol in 150 µL sterile PBS; A_m(normal) = ~11.2 MBq nmol^{-1}) *via* intravenous (i.v.) tail-vein injection (t = 0 h).

For the competitive inhibition (blocking) experiments, a separate aliquot of ^{161}Tb-**12**-onartuzumab (121 µL, 42.19 MBq) was added to a sterile vial. Then an aliquot of the stock solution of formulated onartuzumab (MetMAb, 60 mg mL^{-1}, 183 µL, 11.0 mg of protein) was added to reduce the molar activity (A_m(block) = ~0.355±0.004 MBq nmol^{-1}) and the mixture was diluted with sterile PBS to a final volume of 1.65 mL. Animals in the blocking group (n = 10 mice) each received an i.v. dose containing 3.65 – 3.78 MBq (mean activity = 3.69±0.04 MBq, equivalent to 3.58±0.04 MBq mg^{-1}, 1032.3 – 1033.4 µg of protein, 10.41 – 10.42 nmol in 150 µL sterile PBS). Note: administered doses are reported at the time of injection after decay-corrections and accounting for any residual activity in syringe after administration.

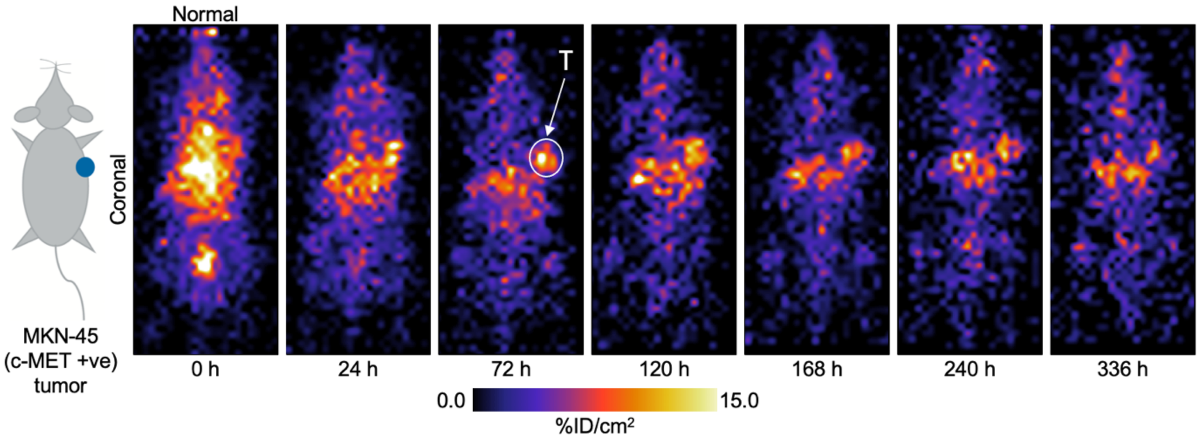

Figure S24. Decay-corrected γ-scintigraphy images of the normal therapeutic group receiving ^{161}Tb-**12**-onartuzumab acquired at different time-points.

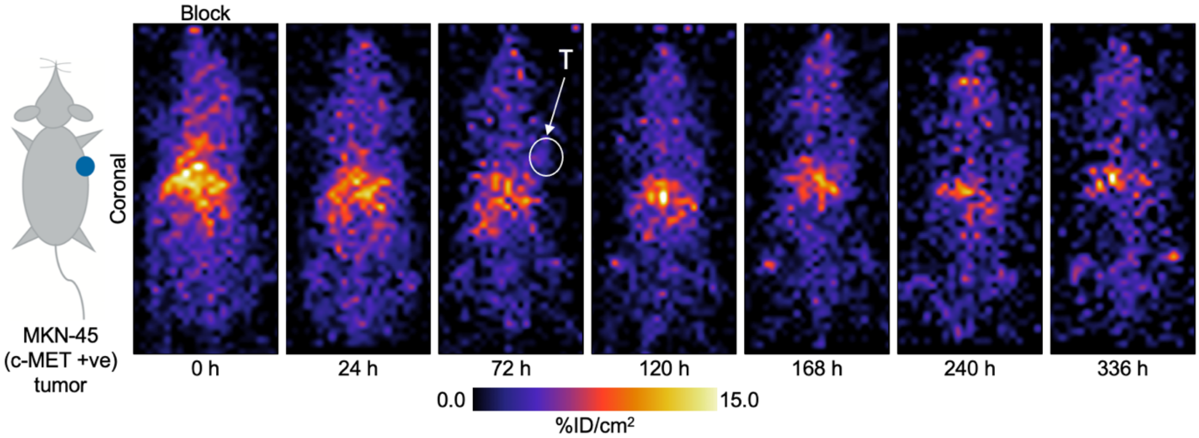

Figure S25. Decay corrected γ-scintigraphy images of the block therapeutic group receiving ^{161}Tb-**12**-onartuzumab acquired at different time-points.

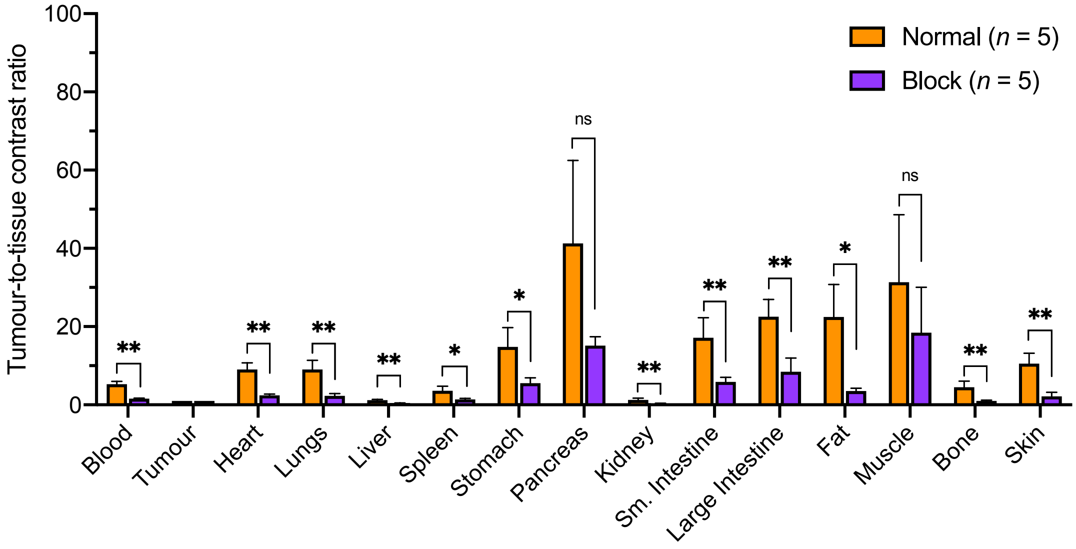

Figure S26. Tumour-to-tissue contrast ratios measured at 72 h post-radiotracer administration in MKN-45 tumour bearing mice for ^{161}Tb-**12**-onartuzumab in the normal (orange) and blocking groups (purple).

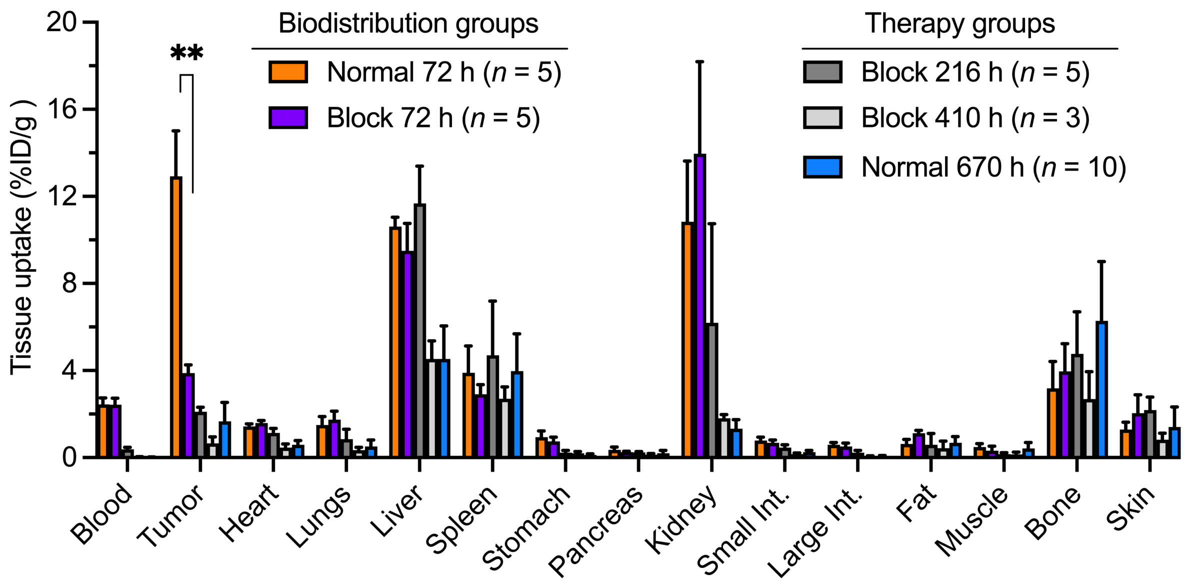

Figure S27. Plot of the tissue uptake (%ID/g) *versus* time from biodistribution studies performed at various time points on the therapy groups.

Table S1. *Ex vivo* biodistribution data measured after i.v. administration of ^{161}Tb-**12**-onartuzumab in female athymic nude mice bearing subcutaneous MKN-45 tumours.

Note: data presented correspond to normal and blocking groups of animals assigned to either the biodistribution only (BioD) experiment or to the therapeutic studies. Time points and the number of animals associated with each data set are specified in the table.

Tissue	^{161}Tb-**12**-onartuzumab, 72 h (BioD Normal group, *n* = 5)		^{161}Tb-**12**-onartuzumab, 72 h (BioD Block group, *n* = 5)		^{161}Tb-**12**-onartuzumab, 216 h (Therapy Block group, *n* = 5)		^{161}Tb-**12**-onartuzumab, 410 h (Therapy Block group, *n* = 3)		^{161}Tb-**12**-onartuzumab, 670 h (Therapy Normal group, *n* = 10)	
	Uptake / %ID g^{-1} ± S.D.[a]	Tumour-to-tissue contrast ratio ± S.D.[b]	Uptake / %ID g^{-1} ± S.D.[a]	Tumour-to-tissue contrast ratio ± S.D.[b]	Uptake / %ID g^{-1} ± S.D.[a]	Tumour-to-tissue contrast ratio ± S.D.[b]	Uptake / %ID g^{-1} ± S.D.[a]	Tumour-to-tissue contrast ratio ± S.D.[b]	Uptake / %ID g^{-1} ± S.D.[a]	Tumour-to-tissue contrast ratio ± S.D.[b]
Blood	2.44 ± 0.31	5.30 ± 0.69	2.44 ± 0.29	1.60 ± 0.09	0.38 ± 0.09	5.74 ± 1.30	0.14 ± 0.16	16.33 ± 13.91	0.02 ± 0.01	78.70 ± 31.57
Tumour	12.93 ± 2.09	1.00 ±	3.89 ± 0.37	1.00 ±	2.10 ± 0.21	1.00 ±	1.16 ± 0.72	1.00 ±	1.67 ± 0.87	1.00 ±
Heart	1.44 ± 0.12	9.03 ± 1.67	1.59 ± 0.12	2.45 ± 0.31	1.13 ± 0.22	1.88 ± 0.23	0.69 ± 0.34	1.80 ± 1.10	0.59 ± 0.19	3.44 ± 2.15
Lungs	1.49 ± 0.39	9.07 ± 2.33	1.74 ± 0.39	2.32 ± 0.57	0.84 ± 0.46	2.84 ± 0.82	0.46 ± 0.18	2.48 ± 1.08	0.51 ± 0.30	2.70 ± 4.05
Liver	10.62 ± 0.42	1.22 ± 0.20	9.50 ± 1.26	0.42 ± 0.07	11.68 ± 1.72	0.18 ± 0.02	6.77 ± 3.21	0.16 ± 0.05	4.53 ± 1.52	0.42 ± 0.26
Spleen	3.90 ± 1.23	3.57 ± 1.22	2.91 ± 0.45	1.37 ± 0.29	4.71 ± 2.49	0.58 ± 0.31	2.47 ± 0.51	0.52 ± 0.38	3.97 ± 1.71	0.36 ± 0.25
Stomach	0.94 ± 0.29	14.83 ± 4.92	0.74 ± 0.20	5.51 ± 1.39	0.22 ± 0.11	13.07 ± 11.13	0.20 ± 0.06	6.32 ± 3.99	0.12 ± 0.05	16.47 ± 10.93
Pancreas	0.36 ± 0.12	41.27 ± 21.19	0.26 ± 0.03	15.13 ± 2.29	0.24 ± 0.05	9.34 ± 2.42	0.19 ± 0.09	5.65 ± 1.15	0.20 ± 0.13	8.24 ± 4.64
Kidney	10.84 ± 2.78	1.27 ± 0.47	13.96 ± 4.23	0.31 ± 0.14	6.19 ± 4.55	0.43 ± 0.17	2.58 ± 1.17	0.43 ± 0.17	1.33 ± 0.42	1.27 ± 0.75
Sm. Int.	0.79 ± 0.16	17.15 ± 5.10	0.68 ± 0.12	5.85 ± 1.19	0.46 ± 0.12	4.81 ± 1.40	0.29 ± 0.21	4.52 ± 2.11	0.26 ± 0.07	7.31 ± 3.31
Large Int.	0.58 ± 0.10	22.51 ± 4.47	0.51 ± 0.16	8.47 ± 3.47	0.23 ± 0.09	10.57 ± 4.85	0.17 ± 0.14	8.68 ± 4.37	0.08 ± 0.02	23.42 ± 9.77
Fat	0.63 ± 0.21	22.48 ± 8.28	1.12 ± 0.12	3.52 ± 0.71	0.60 ± 0.51	6.93 ± 6.56	0.61 ± 0.51	2.60 ± 2.26	0.68 ± 0.29	3.56 ± 2.88
Muscle	0.48 ± 0.16	31.37 ± 17.21	0.31 ± 0.21	18.48 ± 11.61	0.16 ± 0.07	15.45 ± 5.76	0.14 ± 0.09	10.99 ± 8.14	0.42 ± 0.28	5.57 ± 11.63
Bone	3.19 ± 1.22	4.50 ± 1.54	3.97 ± 1.27	1.03 ± 0.19	4.76 ± 1.93	0.57 ± 0.41	4.14 ± 2.19	0.29 ± 0.13	6.28 ± 2.72	0.27 ± 0.14
Skin	1.29 ± 0.35	10.53 ± 2.66	2.04 ± 0.85	2.20 ± 1.00	2.18 ± 0.59	1.02 ± 0.29	1.29 ± 0.70	0.87 ± 0.17	1.40 ± 0.92	1.80 ± 1.09

[a] Uptake data are expressed as the mean %ID g^{-1} ± one standard deviation (S.D. / %ID g^{-1}).

[b] Errors for the tumour-to-tissue ratios are calculated as the standard deviations based on ratios from dependent pairs.

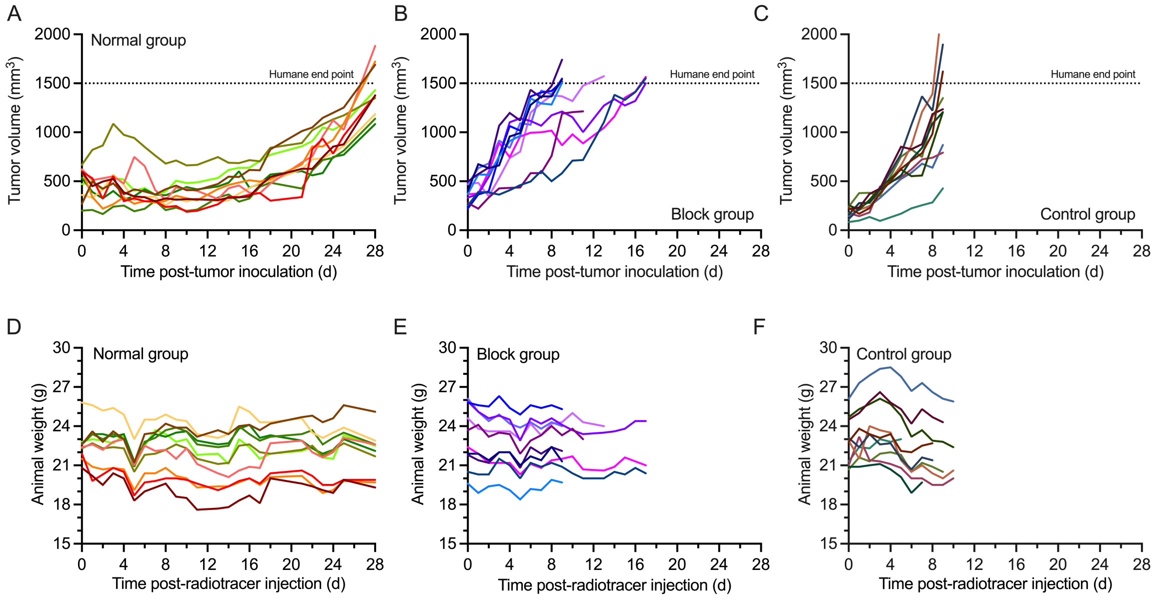

Figure S28. Individual tumour size as accessed per calliper measurement in (A) normal, (B) blocked, and (C) control cohort alongside the individual mouse weight in the (D) normal, (E) block, and (F) control group.

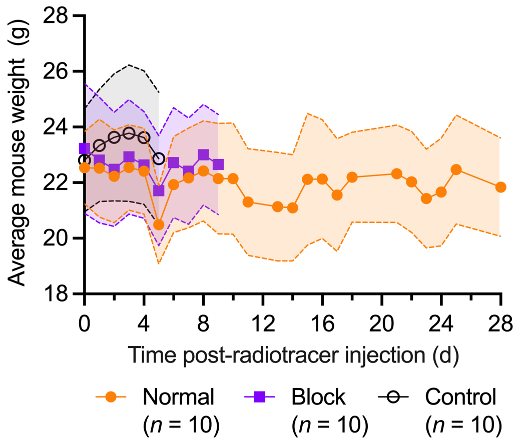

Figure S29. Mean mouse weight in the normal (orange), block (purple), and control (black) groups until end-point criteria were reached (n = 10).

Note: boundaries shown correspond to ±1 standard deviation (S.D.).

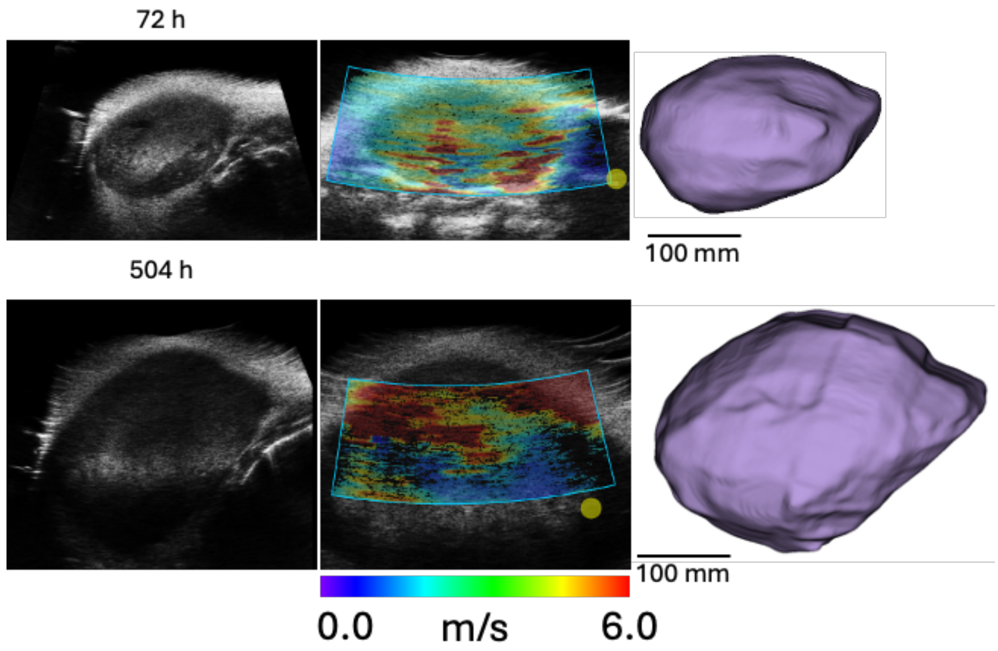

Figure S30. Representative ultrasound images of normal mice showing the tumour morphology at 72 h (top) and 504 h (bottom) time-points in B-mode (left), acoustic radiation force, ARF-mode (middle), and a 3D-render of the tumour (right).

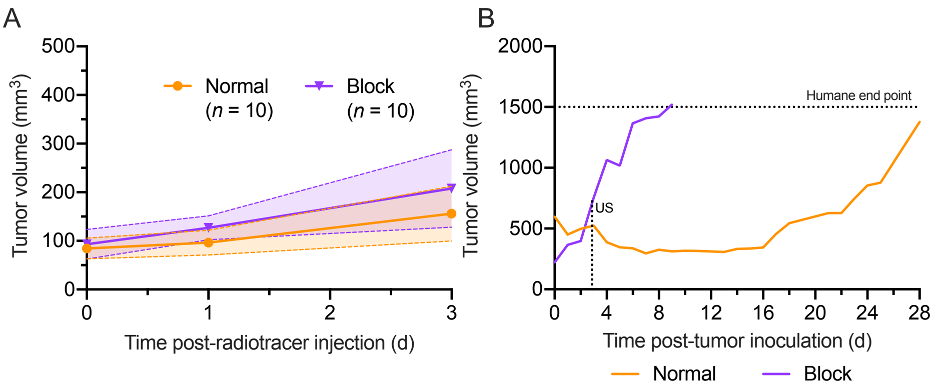

Figure S31. Ultrasound volumetric measurements on tumours up to 72 h post-radiotracer administration.

(A) Average tumour size as measured by ultrasound image analysis in the normal (orange) and block (purple) group in the first 72 h of the study (*n* = 10). (B) Tumour volume (calliper measurements in mm^3) of the selected mice for the US analysis shown in main text.

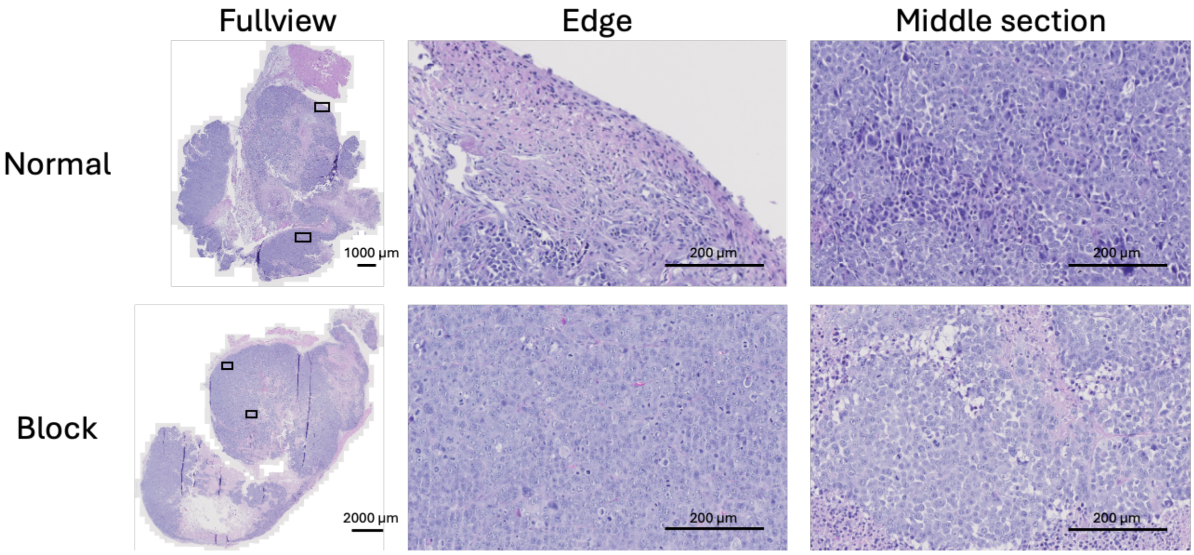

Figure S32. Confocal microscopy pictures of H&E stained tumours after reaching the end-point of the normal (top, 670 h) and block (bottom, 410 h) groups.

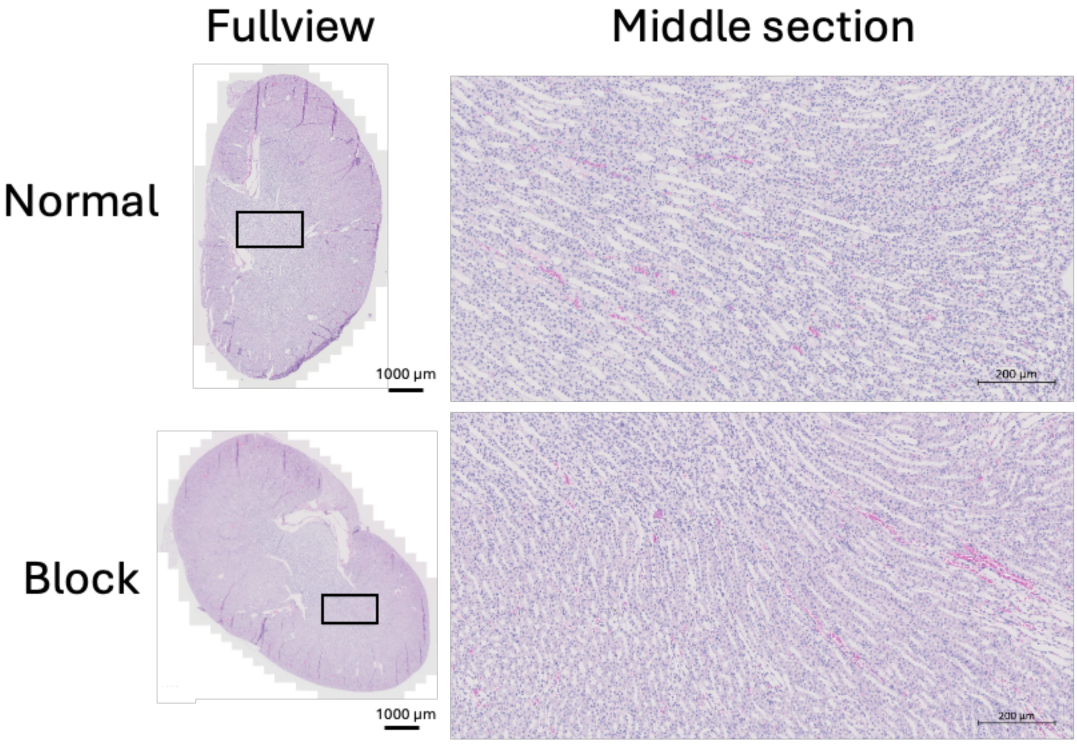

Figure S33. Confocal microscopy pictures of H&E stained kidneys after reaching the end-point of the normal (top, 670 h) and block (bottom, 410 h) groups.

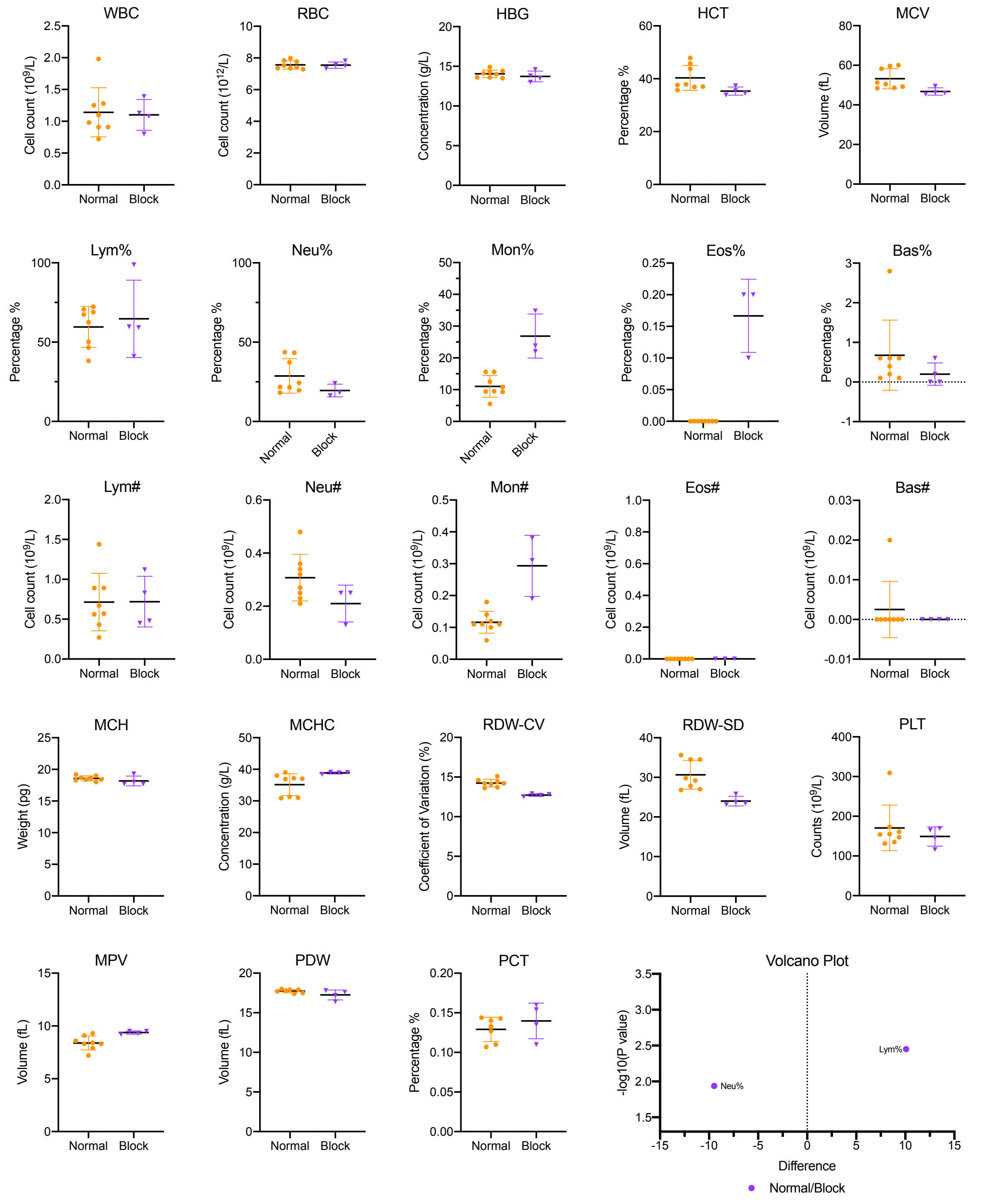

Figure S34. Blood analysis of the samples taken from the normal (orange, 670 h) and blocking (purple, 410 h) groups at the end-point of the respective groups.

Note: WBC = White Blood Cell count (10^9 cells / L); Bas% = Percentage of Basophils (%); Bas# = Number of Basophils (10^9 cells / L); Neu% = Percentage of Neutrophils (%); Neu# = Number of Neutrophils (10^9 cells / L); Lym% = Percentage of Lymphocytes (%); Lym# = Number of Lymphocytes (10^9 cells / L); Mon% = Percentage of Monocytes (%); Mon# = Number of Monocytes (10^9 cells / L); Eos% = Percentage of Eosinophils (%); Eos# = Number of Eosinophils (10^9 cells / L); RBC = Red Blood Cell count (10^{12} cells / L); HGB = Haemoglobin Concentration (g / L); MCV = Mean Corpuscular Volume

(fL); MCH = Mean Corpuscular Haemoglobin (pg); MCHC = Mean Corpuscular Haemoglobin Concentration (g / L); RDW-CV = Red Blood Cell Distribution Width - Coefficient of Variation (%); RDW-SD = Red Blood Cell Distribution Width - Standard Deviation (fL); HCT = Haematocrit (%); PLT = Platelet count (10^9 cells / L); MPV = Mean Platelet Volume (fL); PDW = Platelet Distribution Width (fL) PCT = Plateletcrit (%).

Blood analysis revealed no significant differences between the groups in 21 of 23 measured parameters. The only significant differences identified were in the percentages of neutrophils and lymphocytes. However, the corresponding total cell counts for of neutrophils and lymphocytes between the normal and blocking groups showed no significant differences. Given that the total cell counts are derived from these percentages, the significance of the percentage differences is questionable in the absence of significant variations in the cell numbers.

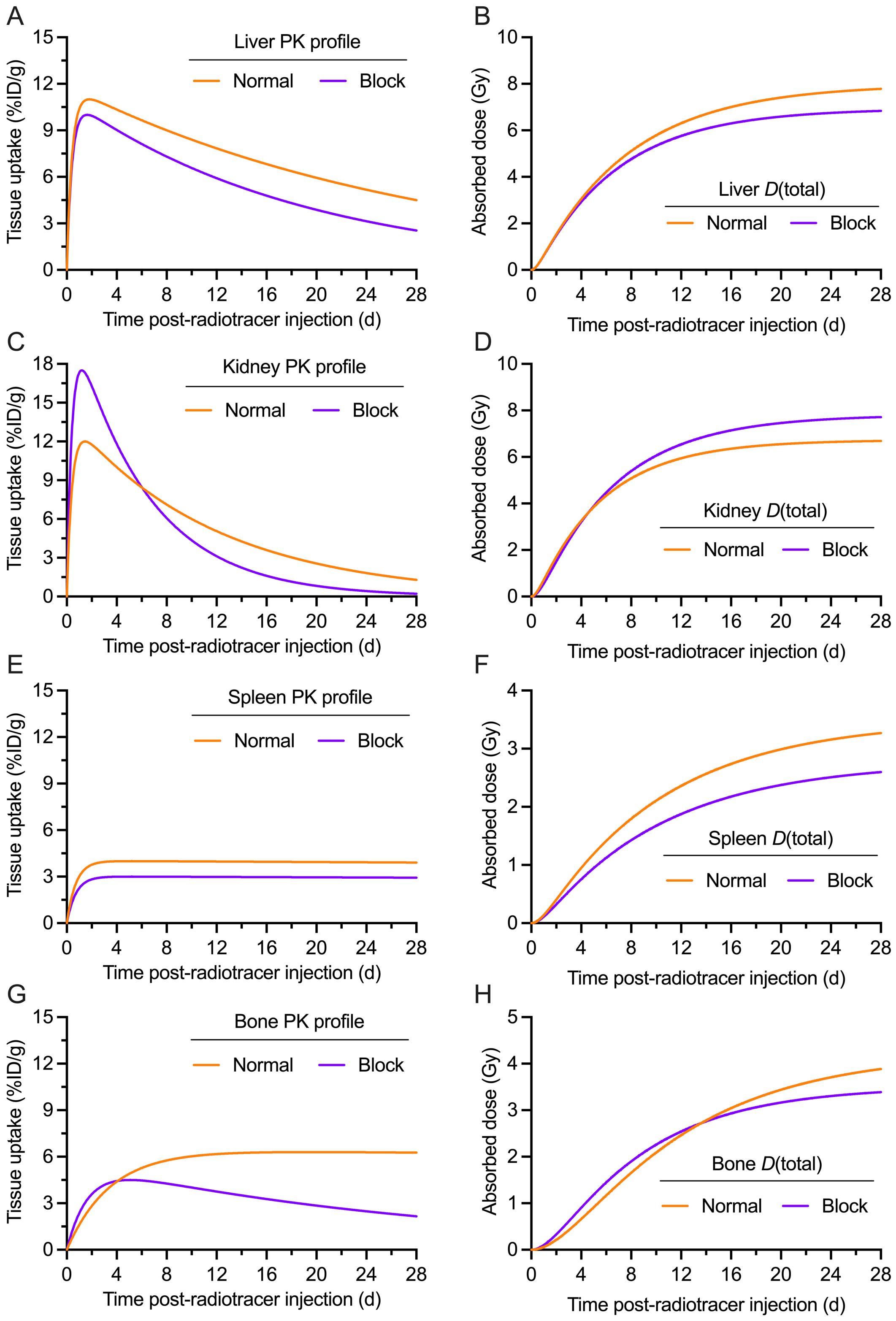

Figure S35. Calculated pharmacological kinetic profile and absorbed doses based on biodistribution and imaging data and for the normal (orange) and block (purple) group in different tissue.

Table S2. Fitting parameters for *DoseItRight*© and calculated absorbed doses in the normal and the blocking group in different tissue.

https://doseitright.streamlit.app

**Normal group (*n* = 10)** Tissue	Half-lives $t_{1/2}$(in) / h	$t_{1/2}$(out) / h	Peak uptake (%ID/g)	Absorbed dose (Gy) 96 h *D*(total)	410 h *D*(total)	670 h *D*(total)	$D(\beta^-)$	*D*(CE)	*D*(Auger)
Tumor	7	205	14	3.8	7.6	7.9	6.2	1.5	0.2
Liver	7	480	11	3.0	7	7.7	6.1	1.4	0.2
Kidney	7	195	12	3.2	6.4	6.7	5.3	1.2	0.2
Spleen	15	*20000	4	1.0	2.8	3.3	2.6	0.6	0.1
Bone	72	*20000	6.3	0.7	3.2	3.9	3.1	0.7	0.1

Mean administered activity, *A* = 3.50±0.13 MBq
Molar activity, A_m = 11.2 MBq/nmol (protein)
Mass, m(mAb) = 31.0±0.1 μg
Moles, n(mAb) = 0.312±0.011 nmol

* $t_{1/2}$(out) >20000 h implies that the radionuclide is sequestered in the tissue with no washout observed

**Block group (*n* = 10)** Tissue	Half-lives $t_{1/2}$(in) / h	$t_{1/2}$(out) / h	Peak uptake (%ID/g)	Absorbed dose (Gy) 96 h *D*(total)	410 h *D*(total)	670 h *D*(total)	$D(\beta^-)$	*D*(CE)	*D*(Auger)
Tumor	7	130	4.6	1.3	2.2	2.3	1.8	0.4	0.1
Liver	7	315	10	2.9	6.4	6.9	5.4	1.3	0.2
Kidney	7	100	17.5	4.8	7.7	7.8	6.1	1.5	0.2
Spleen	15	*20000	3	0.8	2.2	2.6	2.0	0.5	0.1
Bone	35	600	4.5	0.9	3.0	3.4	2.7	0.6	0.1

Mean administered activity, *A* = 3.69±0.04 MBq
Molar activity, A_m = 0.35 MBq/nmol (protein)
Mass, m(mAb) = 1032.7±0.3 μg
Moles, n(mAb) = 10.415±0.003 nmol

* $t_{1/2}$(out) >20000 h implies that the radionuclide is sequestered in the tissue with no washout observed

References

[1] M. J. Frisch, G. W. Trucks, H. B. Schlegel, G. E. Scuseria, M. A. Robb, J. R. Cheeseman, G. Scalmani, V. Barone, G. A. Petersson, H. Nakatsuji, X. Li, M. Caricato, A. V. Marenich, J. Bloino, B. G. Janesko, R. Gomperts, B. Mennucci, H. P. Hratchian, J. V. Ortiz, T. Henderson, D. Ranasinghe, V. G. Zakrzewski, J. Gao, N. Rega, G. Zheng, W. Liang, M. Hada, M. Ehara, K. Toyota, R. Fukuda, J. Hasegawa, M. Ishida, T. Nakajima, Y. Honda, O. Kitao, H. Nakai, T. Vreven, K. Throssell, J. A. Jr. Montgomery, J. E. Peralta, F. Ogliaro, M. J. Bearpark, J. J. Heyd, E. N. Brothers, K. N. Kudin, V. N. Staroverov, T. A. Keith, R. Kobayashi, J. Normand, K. Raghavachari, A. P. Rendell, J. C. Burant, S. S. Iyengar, J. Tomasi, M. Cossi, J. M. Millam, M. Klene, C. Adamo, R. Cammi, J. W. Ochterski, R. L. Martin, K. Morokuma, O. Farkas, J. B. Foresman, D. J. Fox, **2016**.

[2] J. Da Chai, M. Head-Gordon, *Physical Chemistry Chemical Physics* **2008**, *10*, 6615–6620.

[3] P. Pollak, F. Weigend, *J Chem Theory Comput* **2017**, *13*, 3696–3705.

[4] B. P. Pritchard, D. Altarawy, B. Didier, T. D. Gibson, T. L. Windus, *J Chem Inf Model* **2019**, *59*, 4814–4820.

[5] N. Gracheva, C. Müller, Z. Talip, S. Heinitz, U. Köster, J. R. Zeevaart, A. Vögele, R. Schibli, N. P. van der Meulen, *EJNMMI Radiopharm Chem* **2019**, *4*, 12.

[6] C. K. Riener, G. Kada, H. J. Gruber, *Anal Bioanal Chem* **2002**, *373*, 266–276.

[7] M. Georgiou, E. Fysikopoulos, K. Mikropoulos, E. Fragogeorgi, G. Loudos, *Mol Imaging Biol* **2017**, *19*, 398–407.

[8] M. H. Jeong, Y. Choi, Y. H. Chung, T. Y. Song, J. H. Jung, K. J. Hong, B. J. Min, Y. S. Choe, K. H. Lee, B. T. Kim, *Phys Med Biol* **2004**, *49*, 4961.

[9] C. Berton, S. Klingler, S. Prytuliak, J. P. Holland, *npj Imaging* **2024**, *2*, 23.

[10] R. Fay, J. P. Holland, *Chemistry – A European Journal* **2021**, *27*, 4893–4897.
